# Squishy matters – Corneal mechanobiology in health and disease

**DOI:** 10.1016/j.preteyeres.2023.101234

**Published:** 2024-01-02

**Authors:** Sara M. Thomasy, Brian C. Leonard, Mark A. Greiner, Jessica M. Skeie, Vijay Krishna Raghunathan

**Affiliations:** a Department of Surgical and Radiological Sciences, School of Veterinary Medicine, University of California - Davis, Davis, CA, United States; b Department of Ophthalmology & Vision Science, School of Medicine, University of California – Davis, Davis, CA, United States; c California National Primate Research Center, Davis, CA, United States; d Department of Ophthalmology and Visual Sciences, Carver College of Medicine, University of Iowa, Iowa City, IA, United States; e Iowa Lions Eye Bank, Coralville, IA, United States; f College of Optometry, University of Houston, Houston, TX, United States

**Keywords:** Cornea, Mechanobiology, Cell-matrix interactions, Corneal wound healing, Stiffness, Mechanotransduction

## Abstract

The cornea, as a dynamic and responsive tissue, constantly interacts with mechanical forces in order to maintain its structural integrity, barrier function, transparency and refractive power. Cells within the cornea sense and respond to various mechanical forces that fundamentally regulate their morphology and fate in development, homeostasis and pathophysiology. Corneal cells also dynamically regulate their extracellular matrix (ECM) with ensuing cell-ECM crosstalk as the matrix serves as a dynamic signaling reservoir providing biophysical and biochemical cues to corneal cells. Here we provide an overview of mechanotransduction signaling pathways then delve into the recent advances in corneal mechanobiology, focusing on the interplay between mechanical forces and responses of the corneal epithelial, stromal, and endothelial cells. We also identify species-specific differences in corneal biomechanics and mechanotransduction to facilitate identification of optimal animal models to study corneal wound healing, disease, and novel therapeutic interventions. Finally, we identify key knowledge gaps and therapeutic opportunities in corneal mechanobiology that are pressing for the research community to address especially pertinent within the domains of limbal stem cell deficiency, keratoconus and Fuchs’ endothelial corneal dystrophy. By furthering our understanding corneal mechanobiology, we can contextualize discoveries regarding corneal diseases as well as innovative treatments for them.

## 1. Introduction

The cornea is a dynamically responsive tissue, continually exposed to biomechanical stresses and strains, requiring adaptive strategies to maintain its structural integrity. Indeed, the precise structural architecture of the cornea is critical for maintaining its barrier function, transparency, and refractive power. Every cell and tissue in a biological organism are subject to distinct types and degrees of mechanical forces that fundamentally regulates their morphology and fate in development, homeostasis or pathophysiology. This is done through an orchestration of mechanosensation and eliciting an appropriate response through a process known as mechanotransduction, the principles of which are fundamental to studying mechanobiology, or the field of how physical forces affect cellular behavior. Investigations pertaining to corneal mechanobiology are essential to underlying mechanisms of various corneal diseases which may ultimately facilitate the development of novel therapeutic strategies.

The cornea experiences a wide range of mechanical forces from both external and internal sources. Externally, the cornea encounters forces from blinking, eye rubbing, exposure to contact lenses, trauma, and infection. These forces may be atmospheric pressure, compressive, shear, or tensile depending on the source, modality, and recipient cell or tissue type. Further, innate physicochemical properties at the ocular surface interface, such as osmolarity, pH, surface tension, and friction, may interact with underlying mechanical forces to modulate cellular responses. Within the cornea, various cell types may be exposed to different types of mechanical stresses. For example, cells in contact with basement membranes not only perceive the intrinsic mechanical property of the underlying membrane, but also static stretch and/or forces conferred via their structural organization or topography. Another type of force that cells within the stroma encounter is from the compressive nature of corneal lamellar organization and the subsequent dome strain it presents. Thus, the primary corneal cell types - epithelial, stromal, and endothelial cells - are subjected to different, specific mechanical cues and through mechanotransduction respond with alterations in their morphology, migration, adhesion, proliferation, differentiation, gene and protein expression, matrix production, and responsivity to cytoactive factors. Since the extracellular microenvironment is biochemically complex, in addition to the structural support, the ECM mediates receptor-ligand interactions between cells and the ECM at the nano-to micro-scale play critical roles in regulating mechanical and cellular homeostasis. Internally, the primary force imposed on the cornea is intraocular pressure, generated by the balanced production and egress of aqueous humor, and the continual flow of the aqueous humor applies a shear force onto the posterior surface of the corneal endothelium. There are additional biomechanical forces imparted by neighboring structures, including the sclera and lens.

Herein, we will review the recent and rapidly expanding literature on corneal mechanobiology, focusing on the interplay between mechanical forces and cellular responses of the epithelium, keratocytes, and endothelium. We will also explore species-specific differences in corneal biomechanics in order to provide context in the selection of appropriate animal models of corneal diseases. We will discuss the latest research findings, highlight the clinical implications, and shed light on potential avenues for therapeutic interventions in corneal diseases. By deepening our understanding of corneal mechanobiology, we aim to pave the way for novel treatments and improved outcomes for patients with corneal disorders.

## 2. An overview of mechanotransduction

Mechanotransduction is the process by which the environment of a cell or tissue can physically affect intracellular processes. More specifically, it is the cellular biochemical signaling response to extracellular, non-chemical, biophysical stimuli including changes in topography, compliance, shear, compression, extension, gravity, and movement. Understanding the interdependence of cell environment and cell responses through mechanical signaling brings to light another spectrum of details into this relationship that is often overlooked in cellular mechanism research.

Mechanotransduction not only plays a critical role in cell function and viability, but it is also highly impactful in disease states, where extracellular environments are altered and cell confluency within tissues is affected by cell dropout or deposition, thus altering local strains and stresses at the cell surface, and inducing specific cell signaling mechanisms. For example, a key mechanotransducer, transcriptional co-activator with PDZ-binding motif (TAZ), is localized to the nucleus and more highly expressed in corneal endothelial cells with Fuchs’ endothelial corneal dystrophy (FECD), specifically near the site of topographical changes in their ECM known as guttae (Leonard et al., 2023). We will delve more deeply into the importance of TAZ to corneal cells in the **3. Epithelium**, **4. Stroma** and **5. Endothelium**.

A thorough understanding of mechanotransduction in health gives us a more complete story of how cells function in their environment and the cascade of events that initiate the downfall of this relationship in disease. A cell cannot truly exist without its environment, but how integrated and dependent are they on it? What happens first in disease - environmental or cellular changes? A deep dive into the understanding of this interdependency will broaden our understanding of disease progression for the betterment of diagnostics and therapeutics.

### 2.1. Cells are intimately connected via intercellular junctions

**Fig. 1.**
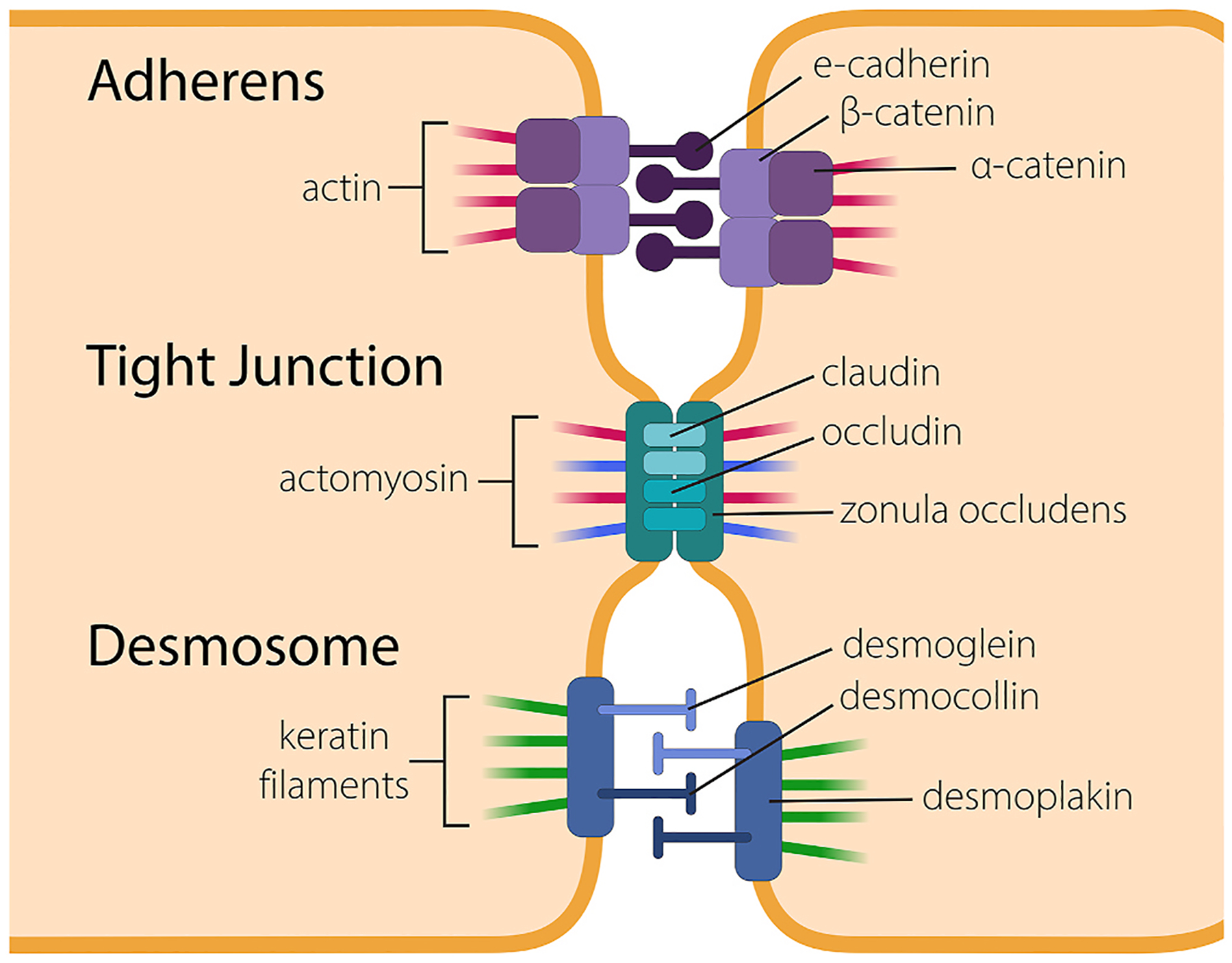
Cells maintain intimate connections via cell-to-cell junctions. The three most common junctions between two cells are the adherens, tight, and desmosome junctions. Cell-to-cell junctions connect two cells but also join the two cells to the cytoskeleton via anchoring proteins on the cellular side of the cell membrane. Junction loss due to disruptions in the cellular monolayer will potentiate changes in signaling through these intracellular cytoskeletal networks.

Junctions between cells are necessary for a layer of cells to become a barrier or tissue. Specifically, monolayers of cells that function as osmotic or physical barriers have a dense organization with respect to each other to provide these functions. Cell-to-cell connections also serve to communicate a tissue’s need for cell repopulation during development or in regenerative tissues. Cells are connected to one another via three main types of junctions including tight junctions, adherens junctions, and desmosomes. *Adherens junctions* are large cell-to-cell contact junctions composed of transmembrane proteins (E-cadherin or VE-cadherin) and intracellular catenins (Bazzoni and Dejana, 2004). Also localized to adherens junctions are the PDZ-domain containing proteins, such as Scribble (Scrib) and Discs large (Dlg), which are important for cellular apicobasal polarity (Nguyen et al., 2005). Intracellular connections to the adherens junctions include actin binding proteins which can transfer signals from the adherens junctions if conformational changes occur to the cytoplasm through these actin binding proteins (Mitten and Baffy, 2022). *Tight junctions* are composed of either claudin or occludin transmembrane proteins in the paracellular space and tethered in the cytosol by zonula occludens proteins 1,2, or 3 (Citi, 2019). Tight junctions are associated with cytoskeletal F-actin, myosin, and cortactin proteins (Bazzoni and Dejana, 2004; Capaldo et al., 2014). Tight junctions are semipermeable to ions and solutes and play a role in the polarity of corneal epithelial and endothelial cells (He et al., 2016; Yoshida et al., 2009). Adherens and tight junctions are critical for the barrier function that the corneal endothelium provides as reviewed here (Srinivas, 2012). For a deeper understanding of tight junction proteins in the corneal epithelium, we direct the reader to this review by Leong and co-authors (Leong and Tong, 2015). Finally, *desmosomes* connect two cells to one another through desmocollins and desmogleins in the intercellular space, which are anchored to plakophilin, plakoglobin, and desmoplakin proteins within the cell near the cell membrane. Desmosomes are connected to the cytoskeleton by desmoplakin interacting with keratin filaments (McMillan and Shimizu, 2001). All three cell junction types have extracellular regions connected to cytoskeletal proteins, creating a direct network from the environment to intracellular signaling; examples of each are shown in Fig. 1 (Zihni et al., 2016).

Cell-to-cell junctions are altered in various ways in a multitude of different scenarios. Indeed, intermittent shear stress dramatically altered corneal epithelial cell morphology as well as mRNA and protein expression of their junctional proteins (Hampel et al., 2018). Hydrodynamic forces modulate transcription of intercellular junction associated genes in the corneal endothelium (Anney et al., 2021). Trauma can cause a wound to the layer(s) of cells, resulting in a disruption to the tissue. For example, healthy, proliferative corneal epithelial cells often divide and migrate to close this disruption, setting up new cell-to-cell junctions as they once again form a confluent layer of cells. During the healing phase, junctions between cells at the boundaries of the trauma are incomplete, thus altering cell-to-cell matrix networking and initiating intracellular signaling cascades, as detailed in 2.3.2 YAP/TAZ and Hippo signaling. Non-proliferative layers of cells migrate and expand to fill the wound, rather than proliferate as exemplified by the corneal endothelium (Ljubimov and Saghizadeh, 2015). Tissue curvature also affects how cells pack in the layers at different radii of curvature and concavity, which is often determined by the curvature dependent deformation of the nucleus (Alias and Buenzli, 2017; Callens et al., 2020). In non-ocular systems, Chen et al. demonstrate that cells distinguish between cell-sized positive and negative curvatures in their physical environment by forming protrusions at positive ones and actin cables at negative ones by as yet unknown molecular mechanisms (Chen et al., 2019). Specifically, they demonstrate that concave edges promote anterograde flow and contractility to induce a tension anisotropy gradient via polarized actin structures with actin flow directed towards the cell edge. This dynamic reorganization enables forward cell migration over non-adherent regions of the substrate. Indeed, in a wounded cornea, corneal epithelial cell migration and re-epithelialization precede reformation of the basement membrane, although an absence of the basement membrane impairs stromal wound healing. More recently, Sonam et al. presented evidence that weakening of cell-cell junctions by cell division events or local cellular stretching promotes high tensile stress resulting in the disruption of the cellular monolayer, which is at least partially regulated by substratum stiffness (Sonam et al., 2023). In an *in vivo* wound, a re-established basement membrane is the new substrate, and its mechanical properties and geometry may significantly regulate cell-cell tension, cell-matrix adhesion and thus barrier integrity. An understanding of how corneal cells sense changes in curvature, geometry, and stiffness of the basement membrane during wounding and ocular surface disorders is lacking and an important topic of future study.

### 2.2. Cells and their ECM create a complex microenvironment

The extracellular microenvironment, or matrix, is a highly developed network of >300 different proteins, comprising ~20% of a person’s body weight, and unique to its surrounding cells and tissues (Mitten and Baffy, 2022). The major constituents include collagens, elastin, glycoproteins, laminin, fibronectin, and proteoglycans, which integrate into a stable structure with distinct mechanical properties (Yue, 2014). *Collagens* are composed of three polypeptide chains twisted into a right-handed helical structure (Xu et al., 2019). Vertebrates have 28 distinct collagen types, including fibril- and network-forming subtypes. Collagens form a stable matrix structure by covalent crosslinks formed by lysl oxidases, glycosylation, and transglutaminases (Xu et al., 2019). *Elastin* is an abundant ECM protein that has the mechanical ability to extend and retract, imparting connective tissues and matrices the capacity to stretch (Skeie and Mullins, 2008). Elastin is most abundant in tissues with frequent stretching/contractile movements, such as blood vessels and lungs (Skeie and Mullins, 2008). *Glycoproteins* are proteins with carbohydrates attached to the chain of polypeptides. Glycosylation of amino acids occurs during posttranslational modification processes of proteins, or even at the time of translation (cotranslational glycosylation). Heavily glycosylated membrane associated mucins are also prevalent at the surface of cells, forming a barrier known as the glycocalyx (Ablamowicz and Nichols, 2016). *Laminins* consist of 11 distinct chains in humans. They are glycoproteins interwoven with ECM proteins at the N-terminus and cell membrane proteins (integrins) at the C-terminus, forming a web structure connecting the ECM and cell surface (Aumailley, 2013). Laminins are most commonly found in basal laminae, which are known as the foundation of cells. *Fibronectin* is also a glycoprotein, and is known as the foundation of the ECM (Garrison and Schwarzbauer, 2021). It has a high molecular weight and adheres cells to the ECM through its binding to cellular transmembrane proteins known as integrins. Fibronectin plays many roles in cell adhesion, cell motility, and cell signaling. *Proteoglycans* are a type of glycoprotein that is heavily glycosylated with carbohydrate chains known as glycosaminoglycans (GAGs). The GAGs are long polysaccharides comprised of repeating disaccharides. Proteoglycans have a protein core with a single or several linear, covalently bound, GAGs branching outward (Wei et al., 2020). Typically, GAGs make up the bulk of proteoglycans (>90%) forming a large “sticky” network which is well suited for connective tissues. While collagens will be extensively discussed for each cell subtype, we will specifically focus on elastin further in 3.4.1. Limbal stem cell homeostasis. We will discuss laminin as a substrate for endothelial cell culture in 5.3 The corneal endothelium and DM are intimately connected, and the critical roles that fibronectin and proteoglycans play in 4.3 Stromal wound healing.

**Fig. 2.**
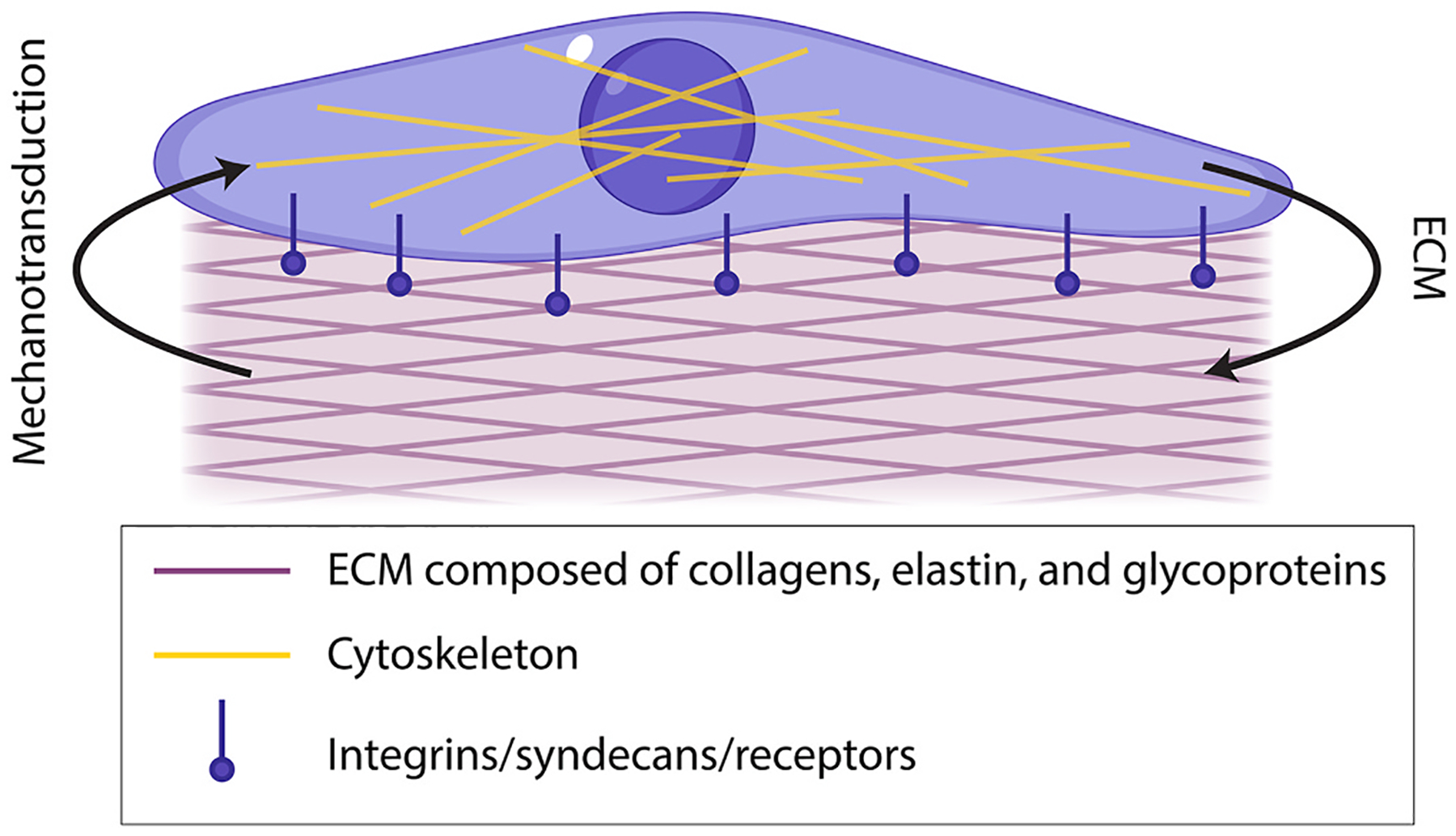
Dynamic reciprocity exists between cells and their ECM. Cells synthesize ECM proteins and deposit them into the extracellular space. The proteins assembled in the matrix present a rich set of topographic and stiffness cues to the cells. Integrins, syndecans, and other receptors mechanotransduce biophysical cues into the cells. Signaling molecules and the cytoskeleton convey these signals to the nucleus, where they influence cell behavior in many ways, including modulating changes in the expression of ECM genes and proteins. Adapted from Ali, M., Raghunathan V., Li, J.Y., Murphy, C.J., Thomasy, S.M., 2016. Biomechanical relationships between the corneal endothelium and Descemet’s membrane. Exp Eye Res 152, 57–70.

Many cells, including corneal cells, exist in bidirectional relationships with their matrices. This means that cells rely on cues and stability from their matrices, while the cells themselves are responsible for producing and depositing these matrices. This type of relationship is known as dynamic reciprocity (Fig. 2) and occurs in a myriad of cell-matrix environments (Bissell and Aggeler, 1987; Bissell et al., 1982). If the balance between cell and matrix is altered, as is often seen in disease, a vicious cycle often occurs. In this cycle, changes in the matrix stimulate altered matrix transcription and deposition by the cell, which in turn further alters the matrix, and so on. A relevant example of this can be observed in FECD as we detail in 5.4. Fuchs’ endothelial corneal dystrophy, an exemplar of primary ECM pathology.

### 2.3. Intracellular mechanotransduction signaling mechanisms

Primary cilia, surface and transmembrane receptors, and junctional proteins transmit and propagate environmental mechanical forces into cells. Examples of these proteins are integrins, syndecans, cadherins, catenins, primary cilia, myosin, ion channels, surface receptors, and zonulae occludens. Integrins have long been studied as fundamental mechanosensors including in the corneal epithelium and endothelium as reviewed here (McKay et al., 2020b). For a comprehensive review of how integrins and receptor-mediated mechanosensing translate into both biochemical and biomechanical cues, we direct the reader to these comprehensive reviews (Chen et al., 2017; Kechagia et al., 2019). For example, Förster resonance energy transfer (FRET)-based tension sensors have demonstrated that cadherin proteins are under tension in cell-cell junctions (Angulo-Urarte et al., 2020). Conversely, cell transmembrane ion channel Piezo1 sits in the cell membrane closed when no membrane tension is present, but it opens under membrane tension. Further, transient receptor potential (TRP) ion channels are a superfamily of proteins that have been identified in sensing and transmitting a wide range of stimuli including osmotic changes, cell membrane curvature, and mechanical stress to regulate volume regulation, calcium signaling and migration (Canales et al., 2019; Nilius and Owsianik, 2011; Pedersen and Nilius, 2007; Yin and Kuebler, 2010). Indeed, TRP channels have been implicated in the pathophysiology of corneal pain (Fakih et al., 2022), dry eye disease (Fakih et al., 2022), inflammatory fibrosis (Okada et al., 2021), and FECD (Cai et al., 2023). As mechanosensors changes their conformation, they induce mechanical shifts that propagate a signaling cascade through interacting linker proteins such as vinculin, talin, and kindlin onto effector proteins like RhoA, Rho associated coiled-coil kinase (ROCK), and actin (Astudillo, 2020). The results of these signaling cascades are transcriptional changes that mediate processes including proliferation, differentiation, cytoskeletal contractility, ECM deposition, and even apoptosis.

Mechanotransduction occurs in every tissue, but the mechanisms can dramatically differ between cell types including in the cornea. Alterations to the extracellular environment, for instance, occur for different reasons, including matrix depositions, cell dropout, and wounds. All of these changes could result in similar intracellular responses, but they may be sensed differently by the cell, or the cellular response may differ. The most conserved intracellular signaling cascades include the Hippo, YAP/TAZ, transforming growth factor-β (TGFβ), protein kinase B (AKT), Wnt, Ras homolog family member A (RhoA), and Piezo1 signaling pathways. A brief description of each pathway is given as well as an overview of crosstalk between the pathways. For a detailed review of cell mechanosignaling particularly for sensing and transducing matrix rigidity, we direct the reader here (Young and Reinhart-King, 2023).

#### 2.3.1. Hippo signaling

The Hippo pathway is highly conserved and its canonical pathway plays multiple roles in cell proliferation, cell apoptosis, cell differentiation, and stem cell regeneration (Ma et al., 2019). The Hippo signaling cascade was named as such because the knockout demonstrated oversized organs, eyes, wings, and limbs, mimicking the appearance of a hippopotamus thus establishing its role in tissue growth and organ size. Dysregulated Hippo signaling has also been implicated in tumorigenesis (Han, 2019). Additionally, non-canonical Hippo signaling has several other functions, including mechanotransduction. Hippo signaling is transmitted through mammalian sterile 20-like 1/2 (MST1/2) and large tumor suppressor 1/2 (LATS1/2) kinases in the cell cytoplasm, which phosphorylate two similar transcription factors, Yes-associated protein (YAP) and TAZ. We note that while there are two major isoforms of YAP (of 8 reported isoforms) derived by differential splicing (Komuro et al., 2003; Sudol, 1994, 2013; Sudol et al., 1995), the most widely studied YAP isoform in canonical and non-canonical Hippo signaling in mammalian cells is YAP1. Further, in humans, the mammalian gene *YAP1* encodes several protein isoforms whose specific functions are poorly understood (Vrbský et al., 2021). When phosphorylated, YAP1 and TAZ are localized in the cytoplasm and often targeted for degradation, although this is context specific and may also interact with other signaling pathways. When dephosphorylated, YAP1 and TAZ are transferred to the nucleus. Henceforth, the YAP1 isoform will be referred to as YAP to remain consistent with the published manuscripts as it pertains to its role in mechanotransduction and in relation to crosstalk with other signaling pathways.

#### 2.3.2. YAP/TAZ and hippo signaling

**Fig. 3.**
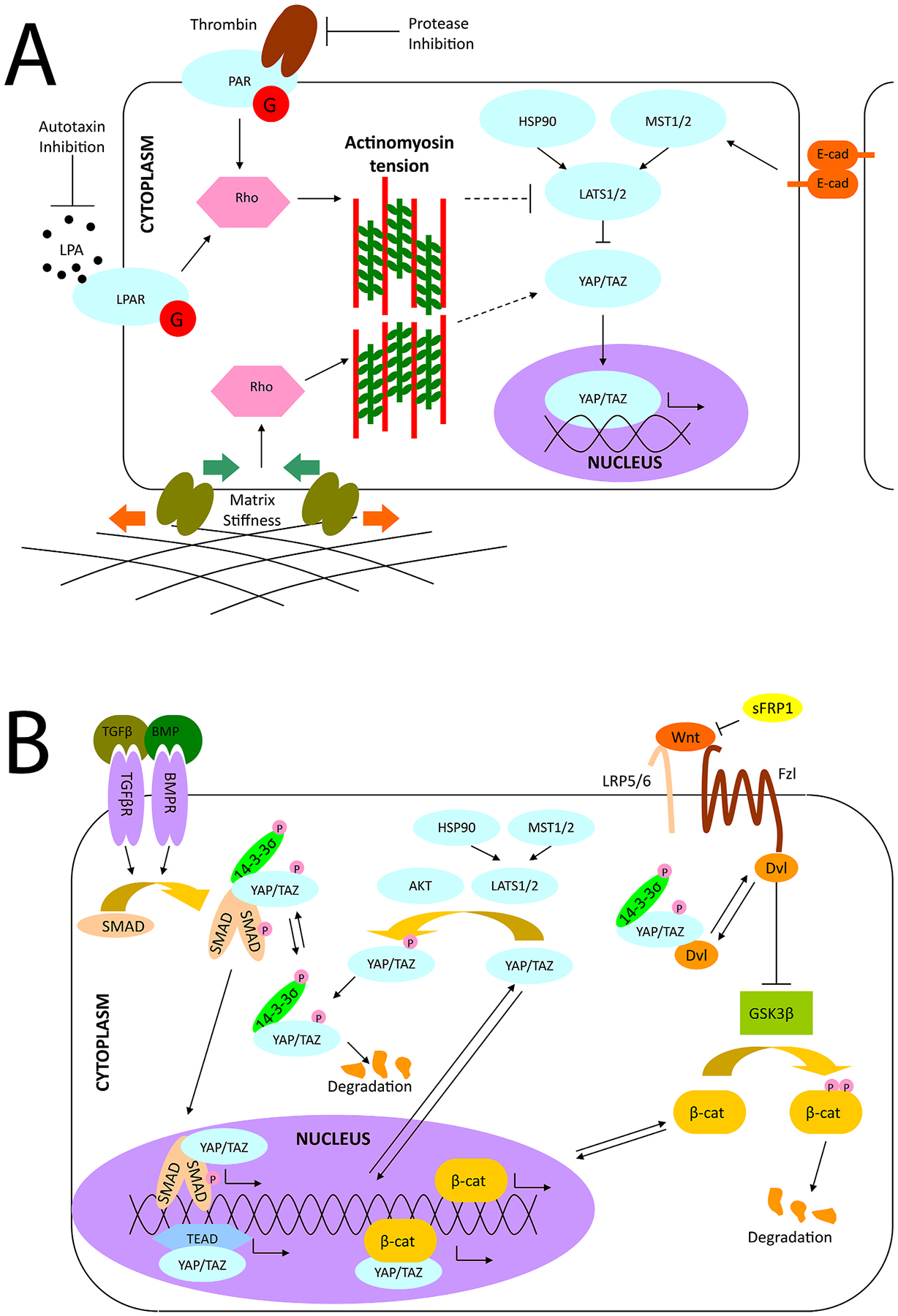
YAP/TAZ are the master conductors of mechanotransduction signaling. **(A)** Mechanical regulation of the Hippo pathway. Hippo is regulated by multiple signals generated by the biophysical (e.g., matrix stiffness) and biochemical (e.g., LPA, thrombin) environment. Importantly, many of these signals are modulated by tension in the actinomyosin cytoskeleton. Modulation of cytoskeletal mechanics through G-protein coupled receptors and matrix biophysics can likewise inhibit YAP/TAZ directly at the nuclear translocation stage or through activation of Hippo components. Note: This schematic is simplified to clarify the major components in the mechanical regulation of YAP/TAZ signaling. **(B)** Crosstalk between YAP/TAZ and TGFβ, and between YAP/TAZ and Wnt. TGFβ superfamily signaling is initiated by the binding of an extracellular ligand (e.g., TGFβ and BMP), which leads to the phosphorylation of SMADs and the formation of a complex with a Co-SMAD and 14-3-3s. After translocation to the nucleus, these complexes initiate the TGFβ/BMP transcriptional program. The SMAD complex also interacts with YAP/TAZ to initiate different transcriptional programs in the nucleus. Canonical Wnt is initiated by the binding of a Wnt ligand to the Fzd/LRP receptor complex. This induces the inhibitory behavior of Dvl on the Axin/APC/GSK3b complex, freeing β-catenin to translocate to the nucleus and initiate the Wnt transcriptional program. YAP/TAZ can inhibit Wnt signaling through inhibition of Dvl in the cytoplasm (TAZ) or in the nucleus (YAP) or cytoplasmic sequestration of β-catenin (YAP). Alternatively, YAP can encourage the transcriptional activity of β-catenin. Note: This schematic is simplified to clarify the intersections of YAP/TAZ and Wnt signaling. Reproduced from Ali, M., Raghunathan V., Li, J.Y., Murphy, C.J., Thomasy, S.M., 2016. Biomechanical relationships between the corneal endothelium and Descemet’s membrane. Exp Eye Res 152, 57–70.

Numerous studies have implicated YAP and TAZ as the nuclear conduits relaying mechanical signals sensed by cells (Dupont et al., 2011; Halder et al., 2012; Plouffe et al., 2018; Reggiani et al., 2021; Scott et al., 2021; Tang et al., 2018). Substrate stiffness and cell-cell junctions both play important roles regarding the cellular localization of YAP/TAZ proteins (Fig. 3). On stiffer substrates or among cells without proper tight junctions and confluent layers, YAP/TAZ is localized to the nucleus, where it is active in gene transcription by pairing with transcriptional enhanced associate domain (TEAD) family DNA-binding factors (Pocaterra et al., 2020). By contrast, on soft substrates or among cells in tight monolayers, with proper cell-cell junctions formed, YAP/TAZ is phosphorylated, localizes to the cytoplasm, and can be degraded. Nevertheless, even though YAP/TAZ are often referred to singularly and considered to be functionally redundant, increasing evidence suggests this may not be the case and their function is context and cell type dependent (Muppala et al., 2019), warranting further exploration in corneal homeostasis and disorders.

#### 2.3.3. TGFβ signaling

TGFβ is a cytokine that is released upon mechanical stress (Narimatsu et al., 2015; Piersma et al., 2015a; Varelas et al., 2008) binds to one of its cell surface receptors and activates the phosphorylation of SMAD proteins. Phosphorylated SMAD proteins can bind to YAP/TAZ proteins and facilitate nuclear translocation and initiation of gene transcription as observed in Fig. 3 (Zhang, 2018). TGFβ is a master regulator of corneal wound healing as recently reviewed by Wilson and colleagues (Wilson, 2021). We will extensively discuss the role of TGFβ in the mechanobiology of the **4. Stroma** and **5. Endothelium**.

#### 2.3.4. AKT signaling

The protein kinase B (AKT) signaling cascade is associated with cellular proliferation and survival. Signal initiation occurs through cell surface protein phosphatidylinositol 3-kinase (PI3K) by extracellular signals including matrix interactions, growth factors, cytokines, and hormones. The kinase, AKT, is activated downstream of PI3K and later interacts with mTOR, FOXO, proapoptotic Bcl-2 family protein (BAD), or even YAP/TAZ (Prudowsky and Yustein, 2020; Zhang and Zhang, 2019). Specifically, AKT localizes YAP/TAZ to the cytoplasm through the phosphorylation at serine 127 which facilitates binding to 14-3-3, resulting in cytosolic localization and possible degradation, as shown in Fig. 3 (Basu et al., 2003). Several studies have implicated AKT in corneal endothelial proliferation (Kim et al., 2016; Lee and Kay, 2011; Sabater et al., 2017; Yoon et al., 2020), but its role in mechanotransduction remains understudied.

#### 2.3.5. Wnt signaling

This pathway is initiated through cell membrane receptors, where it then stabilizes intracellular β-catenin, preventing its degradation and allowing for its transcriptional regulation in the nucleus. Alternatively, if Wnt is blocked, β-catenin is degraded, and transcription does not occur as mediated by β-catenin. In canonical Wnt signaling, the Wnt scaffolding protein Dishevelled (Dvl) associates with frizzled protein to free β-catenin from cytosolic degradation and facilitate its nuclear localization (Fig. 3). YAP/TAZ proteins can play both positive and negative roles in Wnt signaling. In their seminal work, Azzolin and colleagues demonstrated that YAP/TAZ are components of the β-catenin destruction complex and act as β-catenin inhibitors in the Wnt OFF state while they can act as β-catenin activators in the Wnt ON state (Azzolin et al., 2014). Further, Dvl plays a critical role in both the cytoplasmic translocation of phosphorylated YAP and in the nuclear localization of YAP induced by E-cadherin, adenosine monophosphate-activated protein kinase, and α-catenin (Lee et al., 2018; Piersma et al., 2015b). Thus YAP/TAZ can signal both upstream and downstream in regulating cell fate decisions (Totaro et al., 2018). YAP/TAZ proteins also stabilize β-catenin, providing another mechanism by which β-catenin transcription can occur (Piersma et al., 2015a). The importance of Wnt in the corneal stroma will be reviewed as it relates to the pathogenesis of keratoconus 4.6 Keratocytes respond to mechanical stretch as well as its role in 4.8 Keratocyte mechanotransduction. We will also review how Wnt signaling impacts corneal endothelial to mesenchymal transition (EnMT) in 5.3. The corneal endothelium and DM are intimately connected.

#### 2.3.6. RhoA signaling

RhoA is a critical link between mechanical stimuli and the cytoskeletal protein actin (Burridge et al., 2019). RhoA is a GTPase, constantly in a cycle of GDP and GTP states via the counteractive activities of guanine nucleotide exchange factors (GEFs; GDP to GTP) and GTPase-activating proteins (GAPs; GTP to GDP) as well as guanine nucleotide dissociation inhibitors (GDIs; increase GTPases in the cytosol). RhoA-GTP stimulates ROCK which phosphorylates myosin light chain to enhance the assembly of myosin filaments, its ATPase activity, and contractile forces on actin filaments (Burridge et al., 2019). Formation of stress fibers and activation of RhoA signaling can also in turn dephosphorylate YAP/TAZ to activate its mechanotransductive role (Esposito et al., 2022; Ohgushi et al., 2015; Scott et al., 2021; van Soldt and Cardoso, 2020); a possible role for nuclear shape alterations to increase nuclear import of YAP/TAZ as a result of actin-nucleus interactions has also been postulated (Scott et al., 2021). ROCK inhibition as a therapeutic strategy for FECD will be examined in 5.6 Therapeutics that modulate endothelial-DM interactions.

#### 2.3.7. Piezo1 signaling

Piezo1 is a nonselective cation ion channel and is expressed in various tissues (Qin et al., 2021). It is known as the largest pore-like ion channel at ~300 kDa and also as a stretch activated ion channel (SAC) (Zheng et al., 2023). Piezo1 is found integrated into cell membranes or ‘membrane blebs’, sometimes where integrins and ECM form adhesion sites (Lai et al., 2022). The conformational structure of the ion channel alone indicates its role as a mechanosensory protein, as it is closed when the cell membrane is flexible and when the membrane is under force, the channel opens (Qin et al., 2021). Mechanical shear, matrix rigidity, and membrane stretching can open Piezo1 channels. When open, influx of cations (often Ca^2+^) leads to the initiation of intracellular signaling via Yap/Wnt, AKT, or CamKII signaling. Activation of Piezo1 results in two main downstream signaling effects either the release of adenosine triphosphate (ATP) or the activation of Ca^2+^-dependent calpain enzymes, which are cysteine proteases (Lai et al., 2022). Piezo2-Piezo1 crosstalk has been implicated in aberrant neural regeneration activity in systemic lupus erythematosus patients with concurrent dry eye (Sonkodi et al., 2023a, 2023b). However, its role in other corneal neurodegenerative diseases is understudied.

#### 2.3.8. Calcium-mediated signaling

Calcium can regulate several cell processes including cell cycle, cytoskeletal remodeling, muscle tissue repair, and apoptosis (Benavides Damm and Egli, 2014). Calcium fluctuations occur through ion channels at the cell surface, such as Piezo1, or releases from intracellular stores, such as the endoplasmic reticulum (ER). Calcium binds to calmodulin (CaM), which in turn activates calcineurin, a phosphatase that facilitates the nuclear translocation of nuclear factor of activated T cells (NFAT) via its dephosphorylation. Downstream NFAT signaling has been implicated in pathological matrix deposition (Wang et al., 2010). We discuss calcium signaling further in 4.8 Keratocyte mechanotransduction.

### 2.4. An overview of mechanotransduction in disease

**Fig. 4.**
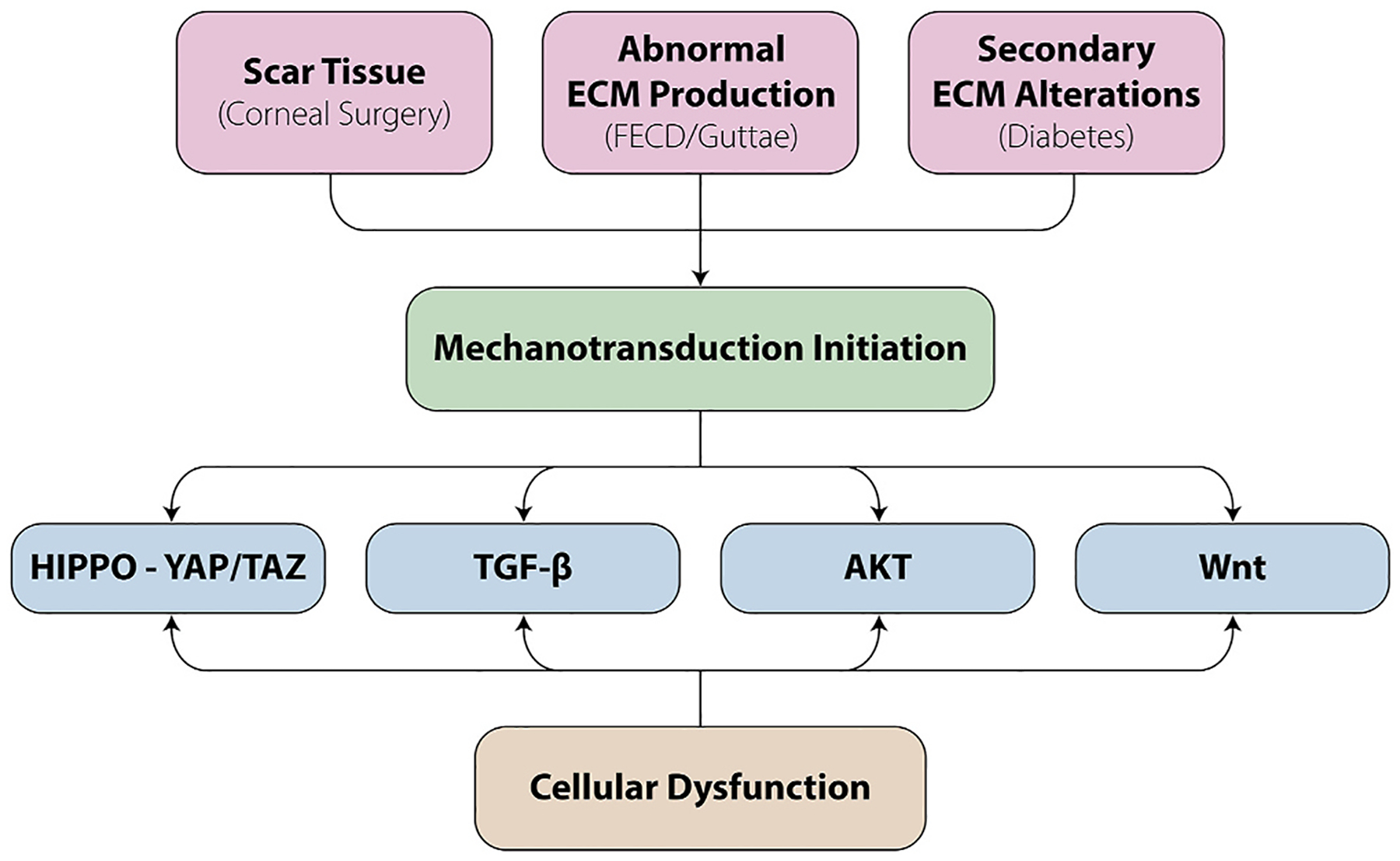
Mechanotransduction feedback mechanisms can drive disease. Mechanotransduction can be initiated in disease states through many different cell or matrix alterations including the deposition of new matrix, change to ECM composition, cell-to-cell junction alterations, cell-to-cell junction loss via cell dropout, and tissue fibrosis/scarring. Downstream propagation on intracellular signaling can occur through many different mechanisms, often converging into pathways mediated by YAP/TAZ, Wnt, TGFβ, and/or AKT. These signaling cascades ultimately yield alterations in gene transcription that can propagate cellular dysfunction.

Disease alters both the cell and its extracellular environment but in different ways in various tissues. By means of dynamic reciprocity, these alterations are often found to be in a vicious cycle of change, with an indiscernible initiation point. We commonly hear the question in life: “Which came first, the chicken or the egg?” In mechanobiology research that often becomes rephrased as: “Which came first, the matrix alterations or the intracellular signaling?” Although pinpointing the exact origin of dysfunction in disease remains a challenge, determining the details within the revolution of this altered cycle can help scientists optimize when and what to target for treatment. Corneal diseases where mechanobiology is critical to the pathophysiology include keratoconus, corneal fibrosis, FECD, and diabetes mellitus while it remains understudied in other disease states such as limbal stem cell deficiency. Disease impacts the cell-cell junctions and cell-matrix relationships in different ways, while initiating mechanotransduction at the cell surface, with important downstream effects (Fig. 4). The study of mechanotransduction in corneal disease is in its infancy, and is far less characterized than in the oncology (Broders-Bondon et al., 2018; Chin et al., 2016), dermal fibrosis (Duscher et al., 2014; Kuehlmann et al., 2020; Yin et al., 2022), and atherosclerosis literature (Conway and Schwartz, 2013; Dessalles et al., 2021; Hahn and Schwartz, 2009; Hamrangsekachaee et al., 2023; Leclech et al., 2020; Mehta et al., 2021; Yamashiro and Yanagisawa, 2020). Ongoing research demonstrates the key roles of the mechanotransducers, YAP and TAZ, in corneal epithelial, stromal, and endothelial cells. In this review, we discuss new discoveries in corneal mechanotransduction, highlighting what is currently known about mechanobiology in the corneal epithelial cells, the stroma, and the endothelial cells while also highlighting key understudied areas.

## 3. Corneal epithelium

### 3.1. Anatomy and physiology of the corneal epithelium

**Fig. 5.**
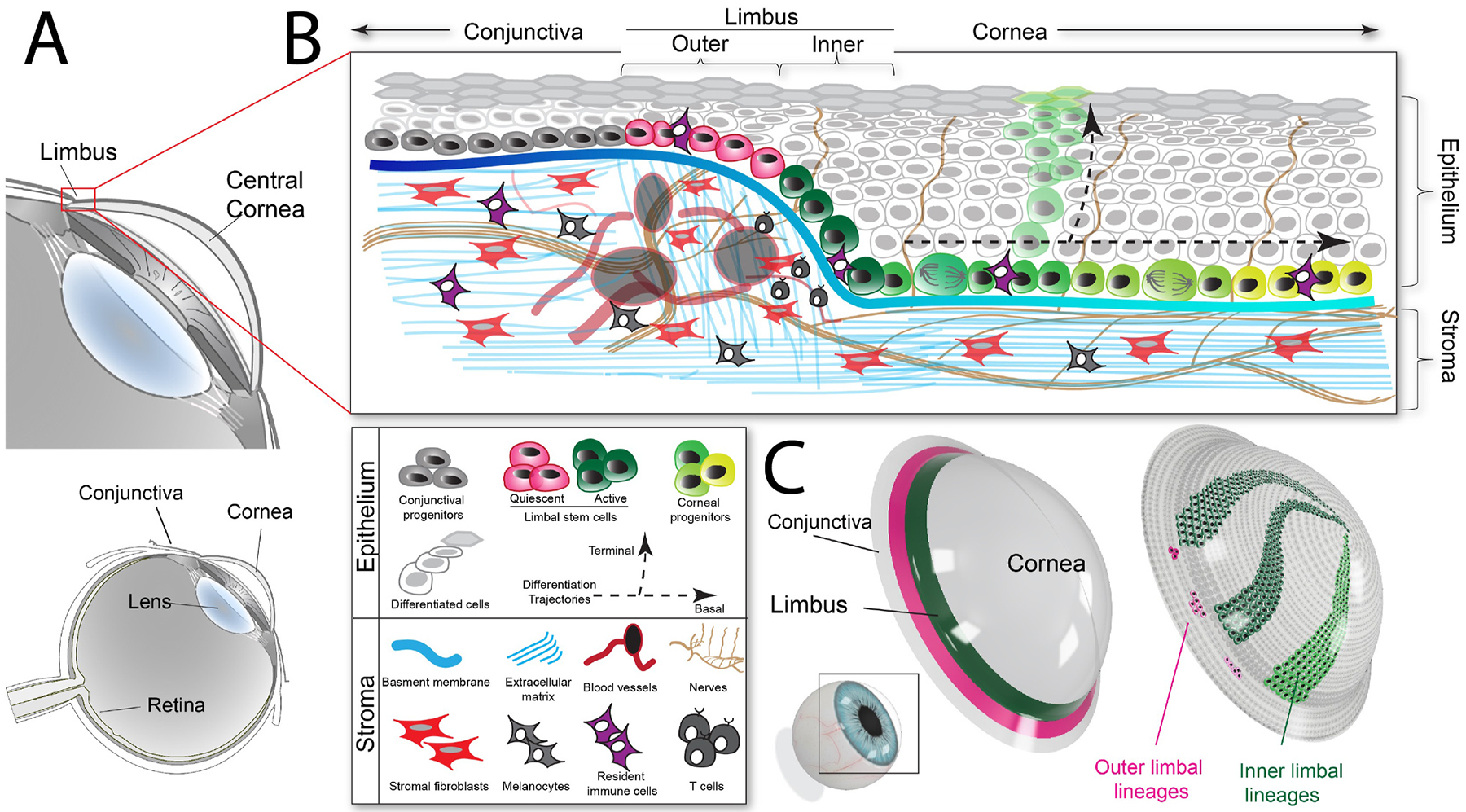
Compartmentalized organization of stem cells in the ocular-surface epithelium. (A) Schematic of the anatomy of the mammalian eye. The inset shows a magnification of the ocular anterior segment and the location of the cornea and limbus. **(B)** Cross-section diagram of the mouse cornea illustrating the components of the limbal stem cell niche. The limbus is located at the intersection between the conjunctival and corneal epithelia, which share a common surface. Stem cells and progenitors are located in the basal layer of the stratified epithelium. At least two distinct stem cell populations exist in the limbal niche. Mostly quiescent stem cells are located in the outer limbus. These stem cells do not contribute directly to the homeostasis of either the conjunctiva or the cornea but can be activated after large-scale injury to the corneal epithelium. Stem cells in the inner limbus are active and undergo mostly symmetric cell divisions to generate transient-amplified progenitors. These exit the limbus and drift centripetally while continuing to proliferate. Cells in the basal layer can commit to terminal differentiation by intrinsic or extrinsic cues, to replenish the cells that are shed from the epithelial surface. The probability of basal progenitors committing to terminal differentiation increases toward the center of the cornea. The stroma consists of cellular and noncellular components that can regulate and influence the activity and fate of stem cells. **(C)** A 3D model of the cornea showing the organization of the limbal compartments and the clonal behavior of the stem cells within. Reprinted with permission from Lee, V., Rompolas, P., 2022. Corneal regeneration: insights in epithelial stem cell heterogeneity and dynamics. Curr Opin Genetic Dev 77, 101981.

The corneal epithelium lines the external surface of the cornea and consequently, it is exposed to multiple shear stresses, injuries and diseases that alter cellular biomechanics. These changes in turn potently influence cell function. It consists of 5–7 layers of stratified squamous cells, with the deepest basal layer serving as the local progenitor cells that differentiate into wing cells in the middle layers, and eventually giving rise to superficial cells that cover the corneal surface. Additionally, the circumferential cornea is designated as the limbus, harboring the key progenitor stem cells that slowly divide and centripetally migrate to the peripheral, paraxial and axial cornea to replenish the continually shed cells. These various corneal epithelial cells niches are defined by differences in their proliferative capacity, morphology, and gene expression (Fig. 5). This section of the review will focus on the impact of corneal epithelial cell mechanotransduction in homeostasis, disease, and development of therapeutics.

### 3.2. Components of corneal epithelial mechanotransduction

As discussed in **2. An overview of mechanotransduction**, there are multiple signaling proteins involved in cellular mechanotransduction. The corneal epithelium, tear film, external environment, and continual dynamic movements of the eyelids comprise a unique interface that requires differential sensing mechanisms and responses to insults. This section highlights the specific molecules involved in mechanotransduction by the corneal epithelium.

#### 3.2.1. Glycocalyx

The glycocalyx, a pericellular membrane-bound macromolecule/glycoprotein layer formed above the corneal epithelium, serves as the mechanical interface between the apical epithelial cells and eyelid. The mucins within this layer act as a selective permeability barrier based on size and charge while simultaneously offering protection, controlling tissue hydration and preventing edema formation. For a comprehensive review of the physicochemical interactions between the tear film and epithelium in health and disease, see this manuscript by Yanez-Soto and coauthors (Yañez-Soto et al., 2014). Much of our understanding of the role of glycocalyx in mechanosensation and transduction stems from vascular endothelial cells. A comprehensive review positing the role between this vital glycoprotein layer and force transmission is described elsewhere (Chighizola et al., 2022; Moran et al., 2022). Within the context of the cornea, much of our understanding of the glycocalyx is built upon its roles in lubrication, wettability, and barrier functions (Uchino, 2018). While it is well recognized that they offer a pad for lubrication, there is a continued paucity in our understanding of their role in mechanotransduction per se. Nevertheless, there is precedent in non-ocular systems that the glycocalyx layer, in addition to its role as a buffer/cushion to ease frictional forces, partakes directly in mechanotransduction. For example, in cancer cells the glycocalyx is demonstrated to play critical roles in driving integrin clustering and focal adhesion assembly (Paszek et al., 2014). Degradation of endothelial cell glycocalyx significantly reduces its ability to produce nitric oxide in response to shear stress (Bartosch et al., 2017; Dragovich et al., 2016), and this is partially affected by TRP channels. Together these suggest a strong interaction between the glycocalyx layer and other mechanosensors.

**Fig. 6.**
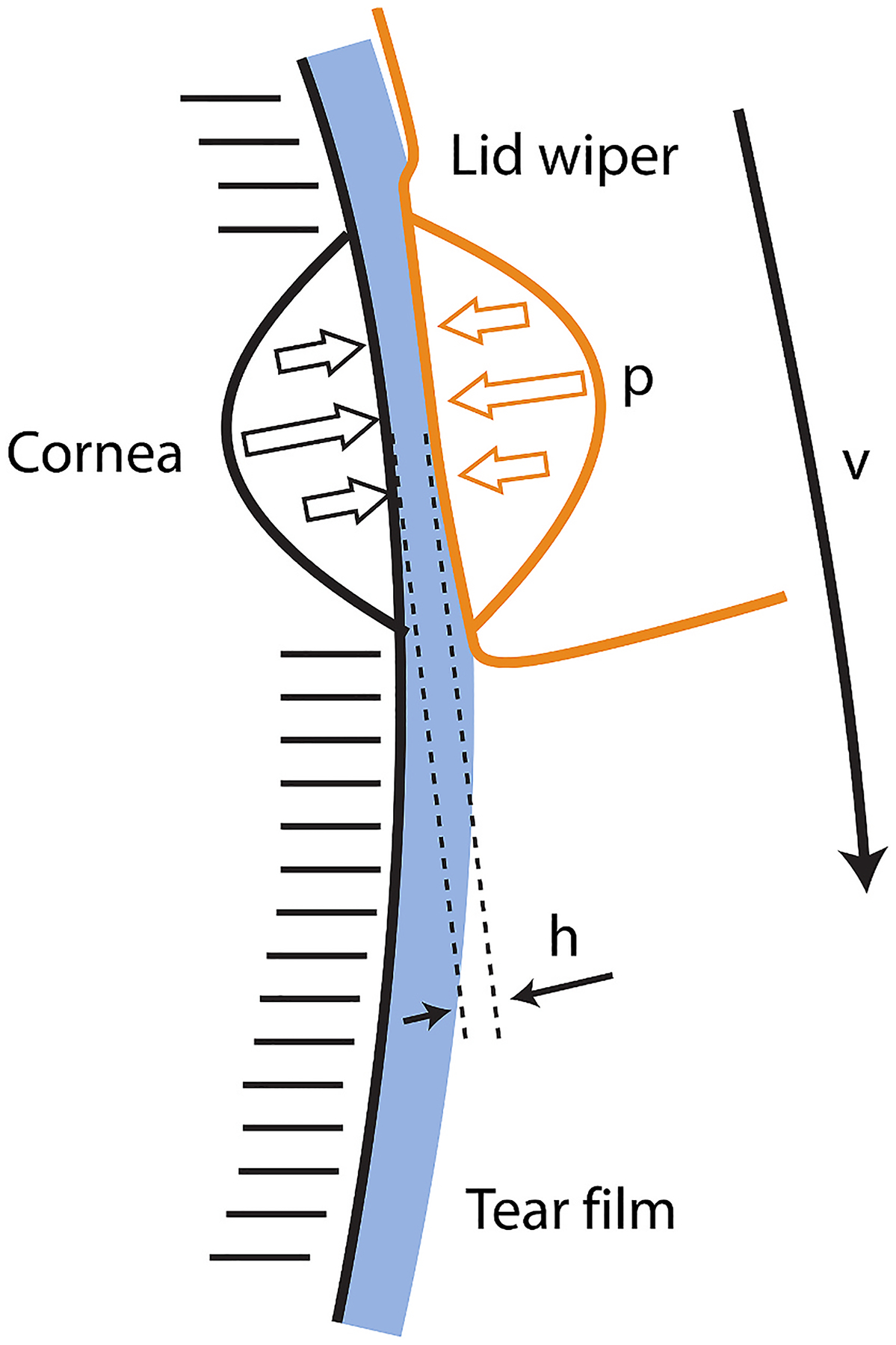
Schematic model of lid – cornea interaction with lid velocity (v), lid pressure against the cornea (p), and tear film thickness (h). Figure and figure legend adapted from Pult, H., Tosatti, S.G., Spencer, N.D., Asfour, J.M., Ebenhoch, M., Murphy, P.J., 2015. Spontaneous Blinking from a Tribological Viewpoint. Ocul Surf 13, 236–249.

Tribology, the science that unites the forces of friction with lubrication to determine the ability of two surfaces to slide over each other, is an important aspect of the ocular surface defined by the interactions of the eyelids and globe (Fig. 6) (Pult et al., 2015). Importantly, the presence of the conjunctival glycocalyx on the inner aspect of the eyelids and the corneal epithelial glycocalyx extending from the apical surface of these cells interact to reduce friction, permitting the movement of the eyelids over the cornea with minimal friction and wear (Lievens and Rayborn, 2022). Alterations in the composition of the glycocalyx, seen with dry eye disease, or the use of contact lenses that prevent these interactions, can lead to increased friction and generate symptoms of ocular surface disease (Lievens and Rayborn, 2022). Thus, a more thorough understanding of tribology and these forces in disease will be important in the design of therapeutics that re-establish these interactions to promote ocular surface health.

#### 3.2.2. Integrins

Multiple integrins, including α6β4 and α3β1, are expressed by corneal epithelial cells and play important roles in corneal development, wound healing and maintenance in the stem cell niche (McKay et al., 2020a). In murine knockout models of either α6 or β4 integrin subunits, mice shed their skin soon after birth due to an inability of keratinocytes to form hemidesmosomes connecting the cells to their basement membrane (Dowling et al., 1996; Georges-Labouesse et al., 1996). The integrin α6β4 is highly expressed in corneal epithelial cells, particularly those found in the limbal stem cell niche (Pajoohesh-Ganji and Stepp, 2005; Polisetti et al., 2016). Importantly, the basement membrane composition of the cornea varies from the limbus to central cornea (Ljubimov et al., 1995, 1998), therefore higher expression of α6β4 may be required to optimize the integrin to ECM connection, thus promoting the maintenance of stem cell progenitors at the limbus. These important findings have led to the fabrication of ECM composite substrates that maintain corneal limbal stem cell phenotypes for transplantation in patients with limbal stem cell deficiency (Polisetti et al., 2017).

#### 3.2.3. Focal adhesions and the epithelial cytoskeleton

Focal adhesions form the linkages between integrin receptors and the cytoskeleton of the cell. Upon activation through the clustering of integrins, multiple proteins are recruited to focal adhesions, including focal adhesion kinase (FAK). Activated FAK recruits additional signaling molecules including vinculin (Brown et al., 1996), paxillin (Hildebrand et al., 1995; Turner and Miller, 1994), talin (Chen et al., 1995), p130Cas (Cary et al., 1998), and Src family kinases (Cobb et al., 1994), to influence cell cycle, survival and proliferation (Lu and Rounds, 2012; Owen et al., 2011; Zhao et al., 1998, 2000). The recruitment of these signaling proteins to the focal adhesions further transmits mechanical information via the cytoskeleton, including F-actin and intermediate filaments, towards the nucleus. The link between the cytoskeleton and the nucleus is further mediated by a network of nuclear membrane proteins, including lamins, emerins and nuclear envelope spectrin repeat proteins (nesprins) (Alam et al., 2014). The significance of FAK and the expression of nuclear proteins will be further discussed in the context of corneal epithelial cell responses in 3.3.1 Effect of topographic cues. Extracellular signal regulated kinase (ERK) is an important mediator of cell migration to promote wound closure and ERK has been shown to from complexes with FAK and paxillin to facilitate this closure. Using an *in vitro* scratch assay, wounding of the human corneal epithelial cells resulted in formation of an ERK-FAK-paxillin complex to promote epithelial wound healing (Teranishi et al., 2009). Conversely, inhibition of ERK signaling with PD98059 inhibited corneal epithelial wound healing (Teranishi et al., 2009). These results identify the importance of ERK activation and subsequent formation of the ERK-FAK-paxillin complex to enhance corneal epithelial wound healing. In a murine model of herpesvirus keratitis, FAK, PI3K and AKT expression was significantly increased over baseline conditions and resulted in the upregulation of matrix metalloproteinase (MMP)2 and MMP9. These results suggest that activation of FAK and the formation of the FAK/PI3K/AKT signaling pathway may play an important role *in vivo*, particularly in the context of chronic herpesvirus keratitis (Ke et al., 2019). We highlight that feline herpesvirus type 1 induces a similar keratitis in cats as that observed with herpes simplex virus type 1 in humans and is thus an additional model to consider for studying therapeutics that modulate FAK and related pathways (Pennington et al., 2017; Thomasy and Maggs, 2016).

#### 3.2.4. Stretch-activated ion channels

The TRP proteins comprise a group of cation channels, serving as molecular sensors for environmental cues (Liu and Montell, 2015). A wide variety of stimuli have the potential to activate TRP gating including vision (Hardie and Minke, 1992; Montell and Rubin, 1989; Xue et al., 2011), taste (Kim et al., 2010; Zhang et al., 2003), smell (Colbert and Bargmann, 1995; Kwon et al., 2010; Liman et al., 1999), hearing (Kamikouchi et al., 2009; Sidi et al., 2003), proprioception (Li et al., 2011; Walker et al., 2000; Yan et al., 2013), and thermosensation (Caterina et al., 1997; McKemy et al., 2002). More recently, studies have suggested that TRP channels can be activated by mechanical forces, directly through TRP channels or regional alterations in the lipid bilayer, which opens the TRP with an influx of cations. This model is supported by the presence of a cluster of ankyrin repeats in the N-terminus of some TRP channel sequences that serve as a “gating spring”, opening with external forces (Hill and Schaefer, 2007; Howard and Bechstedt, 2004; Li et al., 2006; Sotomayor et al., 2005; Story et al., 2003; Walker et al., 2000). The corneal epithelium has been shown to express TRP vanilloid 1 (TRPV1)-TRPV4, TRP canonical 4 (TRPC4) and TRP ankyrin 1 (TRPA1) (Mergler et al., 2011; Yamada et al., 2010; Yang et al., 2003, 2013; Zhang et al., 2007), suggesting that stretch-activated TRP channels may play a role in corneal mechanotransduction. For example, TRPC4 is thought to control cell proliferation and migration through interactions with MAPK and EGFR signaling in the corneal epithelium (Yang et al., 2005). In a rat corneal organ culture with epithelium debridement, activation of the TRPV1 signaling pathways led to enhanced reepithelialization of the corneal surface and inhibition of TRPV1 signaling slowed this healing process (Sumioka et al., 2014). A similar finding was seen *in vivo* as mice lacking TRPV1 demonstrated impaired re-epithelization with reduced proliferation and migration (Sumioka et al., 2014). Collectively, these findings demonstrate the importance of TRP channels in corneal epithelial wound healing and suggest that modulation of their activity may serve as a novel therapeutic approach for recurrent nonhealing erosions.

### 3.3. Impact of biomechanics on corneal epithelial cell behavior

The study of mechanotransduction is particularly critical to the epithelium given that it is continually challenged by a combination of extrinsic forces including eyelid blinking, contact lens wear, eye rubbing, and intrinsic forces from the epithelial basement membrane and underlying stroma. Increased evidence suggests that mechanotransduction is critical to corneal epithelial cell homeostasis, including the maintenance of the limbal stem cell population, the proliferation of daughter cells and the maturation of corneal epithelial cells transiting to the ocular surface (Masterton and Ahearne, 2018). Additionally, we are developing a better understanding of the influence of biomechanical alterations in ocular surface diseases on corneal epithelial cell behavior and the implications on therapeutic intervention.

#### 3.3.1. Effect of substrate stiffness

**Fig. 7.**
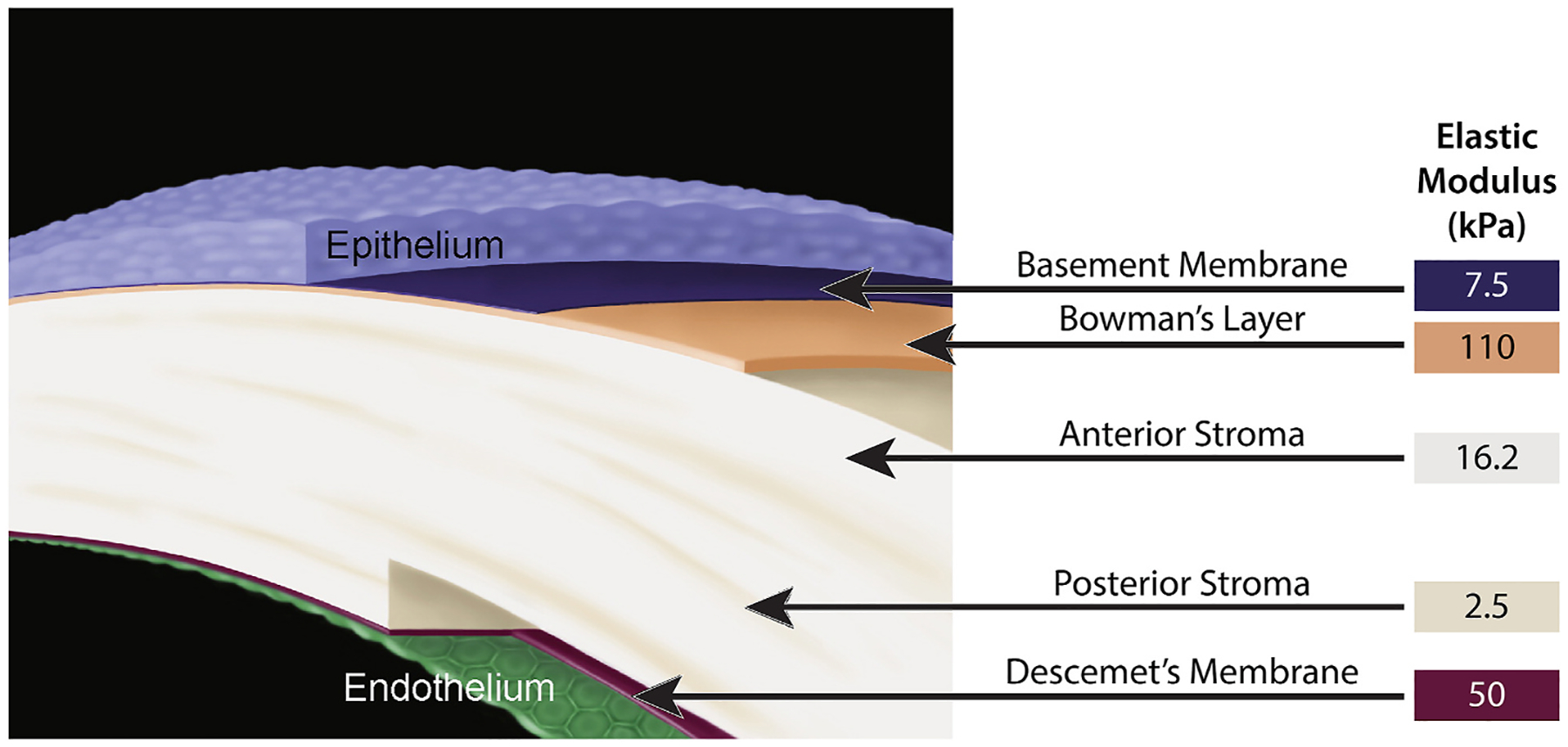
A schematic depicting the layers of the human cornea and the corresponding elastic modulus values obtained from atomic force microscopy: Epithelium, anterior basement membrane (7.5 kPa), Bowman’s layer (110 kPa), anterior stroma (16 kPa), posterior stroma (2.5 kPa), Descemet’s membrane (50 kPa) and the endothelium. Data from (Last et al., 2012; Leonard et al., 2019a). Figure and figure legend adapted from Last, J.A., Thomasy, S.M., Croasdale, C.R., Russell, P., Murphy, C.J., 2012. Compliance profile of the human cornea as measured by atomic force microscopy. Micron 43, 1293–1298.

It has been well-established that cell behavior is directly influenced by both the stiffness of the substrates upon which they reside; however, relatively few studies have focused on the corneal epithelium (Alauzun et al., 2010; Kauppila et al., 2023; Klenkler et al., 2010). A recent study demonstrated that corneal epithelial cells cultured on stiff substrates (~1.0 MPa) composed of polydimethylsiloxane (PDMS) demonstrated higher proliferative indices (phosphorylated ERK, Ki67) and higher expression of cytokeratin 3, a marker of epithelial cell maturity (Masterton and Ahearne, 2019). Conversely, corneal epithelial cells cultured on softer substrates (10–105 kPa) had lower proliferative behavior and higher expression of cytokeratin 14, a marker of basal epithelial cells (Masterton and Ahearne, 2019). Additionally, cells on softer matrices have been shown to expression higher levels of focal adhesion molecules and intermediate filaments (Masterton and Ahearne, 2019). Overall, these findings suggest that culturing of corneal epithelial cells on substrates of varying stiffness influences cell morphology and behavior, and the use of softer substrates may retain the stem cell-like characteristics of the limbal stem cell population. For comparison, the average anterior basement membrane elastic modulus in humans is 7.5 ± 4.2 kPa (Last et al., 2009) and rabbits is 4.5 ± 1.2 kPa (Thomasy et al., 2014), and is markedly softer in comparison to typical cell culture conditions (Fig. 7). Given that proliferation, morphology and differentiation are all profoundly influenced by substrate stiffness in other corneal cell types (Petroll et al., 2020; Zhang et al., 2023b), studying the impact of stiffness on the influence on corneal epithelial cell behavior is critical to understanding corneal wound healing and tissue engineering. Additionally, multiple cell culture substrates with physiologically relevant stiffness and functionalized with ECM proteins have been created to augment corneal epithelial cell adhesion, proliferation and migration (Alauzun et al., 2010; Klenkler et al., 2005, 2008, 2010; Klenkler and Sheardown, 2006; Merrett et al., 2003).

#### 3.3.2. Effect of topographic cues

**Fig. 8.**
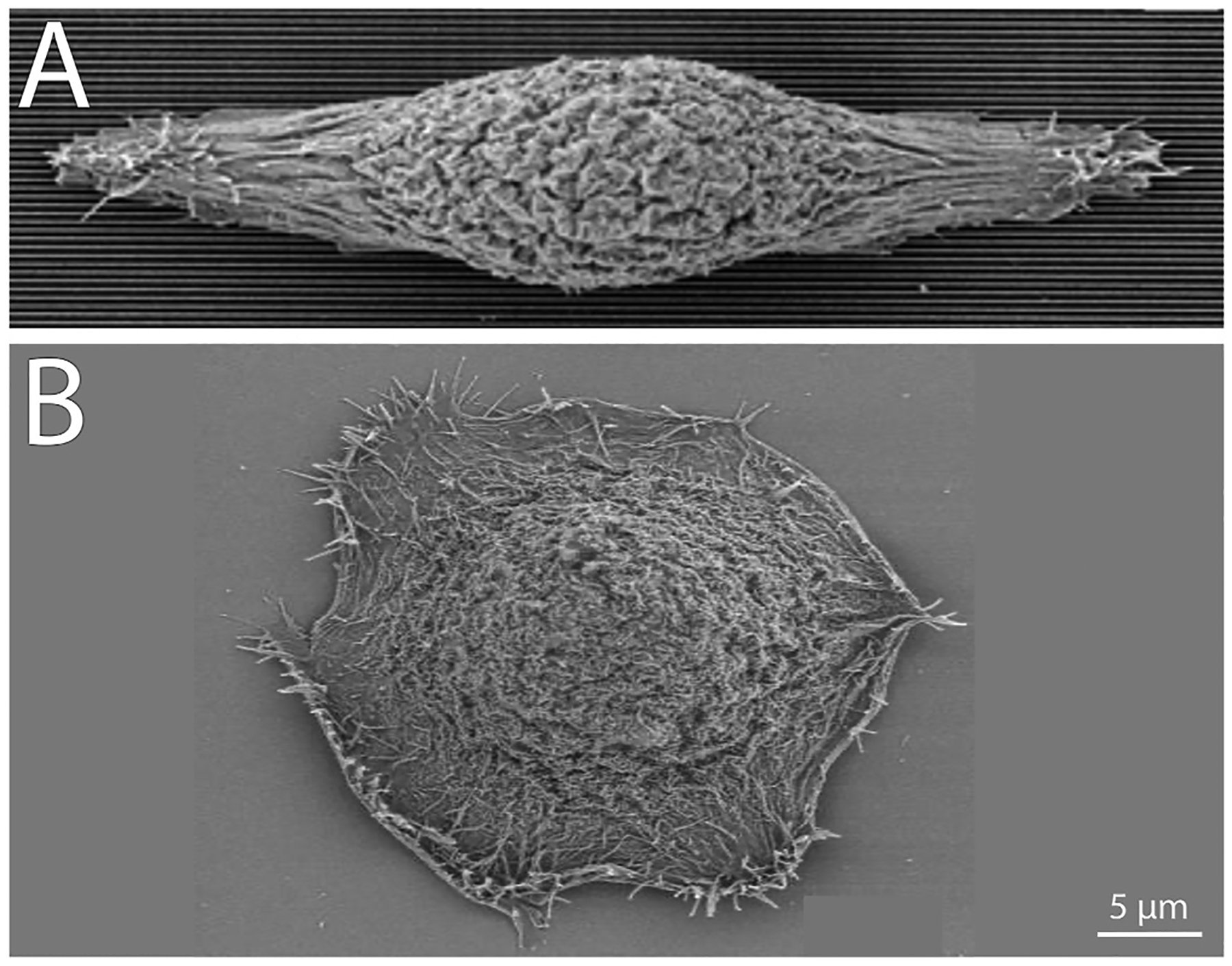
Scanning electron microscopy images of human corneal epithelial cells. (A) Cell cultured on a silicon oxide substrate patterned with 70 nm wide ridges, on a 400 nm pitch. The groove depth was 600 nm. **(B)** Cell on a smooth silicon oxide substrate. Reprinted with permission from Teixeira, A.I., Abrams, G.A., Bertics, P.J., Murphy, C.J., Nealey, P.F., 2003. Epithelial contact guidance on well-defined micro- and nanostructured substrates. J Cell Sci 116, 1881–1892.

In addition to ECM stiffness, the behavior of corneal epithelial cells is influenced by the structure and patterning of the substrate upon which they reside (Diehl et al., 2005; Raghunathan et al., 2013b). The corneal epithelial basement membrane has a rich topography, almost felt-like, with features that range in size from 20 to 200 nm (Abrams et al., 2000a, 2000b, 2002), and is reviewed in detail here (Medeiros et al., 2018). On fabricated anistropic substrates, corneal epithelial cells elongated and aligned themselves along grooves and ridges, whereas they remained mostly round on smooth surfaces (Fig. 8) (Teixeira et al., 2003). The micro- and nanostructured substrates influence the presence of actin microfilaments and focal adhesions (Teixeira et al., 2003). Interestingly, the pitch dimension, overall spacing between ridges, has an impact on corneal epithelial cell behavior. When the pitch dimension was increased from 400 to 4000 nm, there was a decrease in corneal epithelial cell adhesion when a fluid shear force was applied (Karuri et al., 2004). Varying ECM composition and distance between ridges on silk films had differential effects on growth and wound recovery of corneal epithelial cells (Luo et al., 2021). When grown on type I collagen and an 800 nm ridge width, corneal epithelial cells demonstrated enhanced proliferation and substrate adherence that was associated with increased focal adhesion density (Luo et al., 2021). In the absence of fibronectin, corneal epithelial cells were shown to align with anisometropic substrates, however, these cells were unable migrate (Raghunathan et al., 2013a). These results demonstrate the dependence of corneal epithelial cells on ECM proteins for migration and, by extension, corneal epithelial wound healing. On isotropic substrates mimicking the topography of the basement membrane, when cultured as a confluent sheet, electric fields induced collective cell migration compared with dissociated single-cell migration on planar surfaces demonstrating that topographic cues and local electric fields synergistically regulated directional migration of human corneal epithelial cells (Gao et al., 2015). These findings have direct implications on the design of scaffolds for corneal epithelial transplantation.

A previous study sought to determine the specific influence of topography on corneal epithelial cell behavior through the modulation of YAP and TAZ expression and localization (Raghunathan et al., 2014). It demonstrated that YAP was predominantly responsible for contact guidance in corneal epithelial cells and that YAP modulated the expression of a key ECM regulatory protein, connective tissue growth factor. Additionally, when YAP was forced to translocate to the nucleus using a heat shock protein inhibitor (HSP90), there was increased cell-cell junction formation and co-localization of E-cadherin and β-catenin to adherens junctions, a critical feature of cell polarization (Raghunathan et al., 2014).

Focal adhesion complexes are known to mediate the transduction of mechanical stimuli from cell surface receptors to the cytoskeleton. To determine the role of focal adhesions in the mechanosensing of topography in corneal epithelial cells, FAK expression was inhibited using silencing RNA (Dreier et al., 2012). Contrary to expectations, loss of FAK and focal adhesions enhanced the ability of the corneal epithelial cells to align with patterned surfaces (Dreier et al., 2012). However, knockdown of FAK led to the alteration of nuclear membrane proteins nesprins 1 and 2 expression patterns (Dreier et al., 2012). These data suggest that expression of FAK and the presence of focal adhesions affect the expression of proteins involved in transmitting biomechanical information from the cytoplasm to the nucleus. Collectively, these findings highlight the need to understand the impact of biomechanics on cell behavior, particularly in the context of substrate design for tissue transplantation in the cornea.

### 3.4. Role of mechanotransduction in limbal stem cells

Limbal epithelial stem cells serve as progenitor cells for all cell types that populate the corneal ocular surface. There have been considerable advancements in our understanding of the biophysical properties that define the limbal microenvironment and provide critical input to maintain the limbal stem cell population. Additionally, we are starting to uncover the functional impact of biomechanical changes of disease on the limbal epithelial stem cells.

#### 3.4.1. Limbal stem cell homeostasis

Some of the most recent literature has focused on the role of biomechanics and the maintenance of the corneal limbal stem cell population, demonstrating the importance of YAP in these cells. A study in bovine cornea identified cytoplasmic localization of YAP in limbal stem cells (positive for nuclear p63 expression); however, basal corneal epithelial cells in the central cornea had nuclear localization of YAP (Foster et al., 2014). Additional *in vitro* experiments confirmed that YAP localized to the cytoplasm in bovine corneal epithelial cells cultured on soft substrates (Foster et al., 2014). YAP was also found to be localized in the cytoplasm of limbal epithelial cells from human corneal donors, yet there was nearly absent expression in the axial cornea (Raghunathan et al., 2014). This same study found TAZ to have a predominantly nuclear localization in both limbal and axial corneal epithelium (Raghunathan et al., 2014). The localization of YAP appeared to follow the stiffness of the ECM, with a softer matrix in the limbus (8 kPa) leading to cytoplasmic YAP and a stiffer matrix in the central cornea (20 kPa) leading to nuclear YAP (Eberwein et al., 2014).

**Fig. 9.**
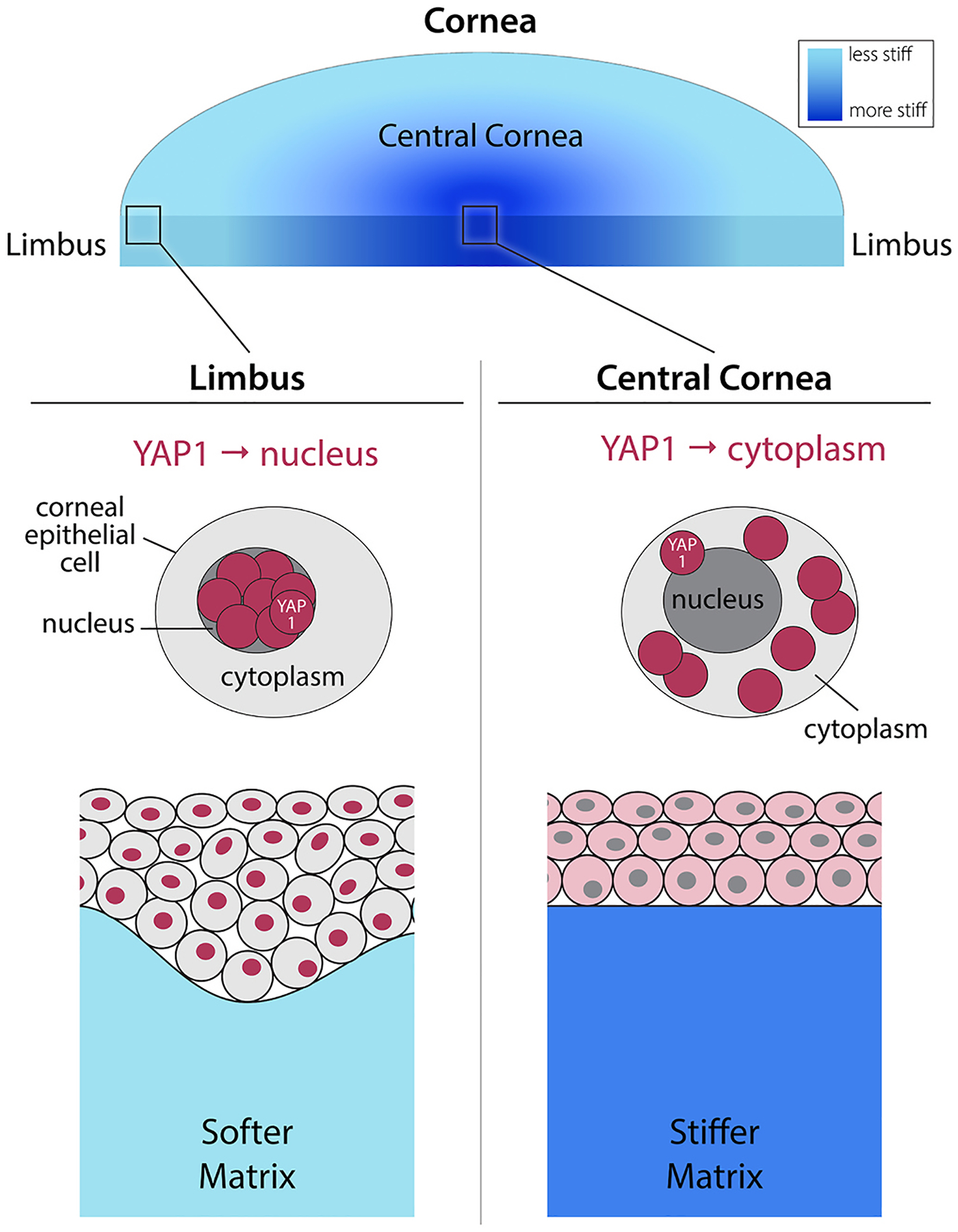
Biomechanical properties of the corneal matrix maintain stem cell populations at the limbus and promote differentiation towards the central cornea. At the limbus, the extracellular matrix (ECM) is softer (light blue) and YAP1 (red circles) is localized to the nucleus of the limbal epithelial stem cells. When moving from the limbal biomechanical niche to the central cornea, there is a transition to a stiffer ECM with cytoplasmic localization of YAP1. The nuclear localization of YAP1 has been shown to maintain the stemness of the limbal corneal epithelial cells, whereas cytoplasmic localization results in the differentiation to more mature corneal epithelial cells (Bhattacharya et al., 2023).

However, more recent studies have demonstrated that *nuclear* localization of YAP is critical for maintenance of the limbal stem cells (Bhattacharya et al., 2023). In the human cornea, nuclear YAP expression was identified in keratin 15 positive (K15^+^) limbal stem cells, closely resembling the keratinocyte molecular phenotype in epidermal stem cells (Bhattacharya et al., 2023). The limbal stem cells were cultured in conditions to induce maturation, leading to a redistribution of YAP to the cytoplasm and upregulation of corneal epithelial cell differentiation markers K3 and K12 (Bhattacharya et al., 2023). In the mouse, differentiated corneal epithelial cells demonstrated activation of kinases in the Hippo pathway, specifically LATS1/2, that phosphorylate YAP and prevent its ability to translocate to the nucleus (Bhattacharya et al., 2023). Similarly, when mice were treated with verteportin, a YAP inhibitor, there were reduced proliferation rates and overall numbers of the limbal stem cells (labeled with CD63 and K15), highlighting a critical role of YAP in maintaining homeostasis of the limbal niche. To determine the role of tissue stiffness on limbal stem cell molecular phenotype, Bhattacharya and colleagues utilized a murine model that overexpresses the lysyl oxidase (Lox^OE^) enzyme to increase covalent crosslinking (CXL) between collagen and elastin in the corneal stroma, thereby increasing stiffness. They found that the 2-fold increase in limbal ECM stiffness of Lox^OE^ mice led to cytoplasmic localization of YAP and corneal opacification, conjunctivalization of the cornea and influx of inflammatory cells, clinical features resembling human limbal stem cell deficiency (Bhattacharya et al., 2023). Lastly, they determined that activation of the TGFβ/SMAD2/3 pathway, a process identified in cells grown on stiff substrates, prevented nuclear localization YAP, and led to loss of the limbal stem cell phenotype. In other cell types, including mouse mammary gland stromal cells (Lerche et al., 2020), murine incisor stem cells (Otsu et al., 2021), and muscle stem cells (Eliazer et al., 2019). YAP does indeed translocate into the nucleus on soft substrates, and therefore, it has been proposed that YAP mechanotransduction is modified by upstream signals, particularly the Rho-A pathway, to maintain stem cell populations. In total, these results suggest that limbal stem cells sense tissue stiffness yet maintain their phenotype by modulating traditional mechanotransduction pathways to promote homeostasis (Fig. 9).

#### 3.4.2. Corneal epithelial regeneration

It is estimated that the corneal epithelium has a complete turnover of cells every 2 weeks in both humans and mice, and the limbal stem cells serve to replenish this continual loss (Buck, 1985; Cenedella and Fleschner, 1990; Cotsarelis et al., 1989). While many limbal stem cell molecular markers have been identified, the advent of single cell sequencing has enabled a more in-depth analysis of this population. Results suggest that a heterogeneous population of limbal stem cells exists, with inner limbal stem cells serving a primary role in homeostasis, while the outer stem cells are directed towards injury repair and regeneration (Altshuler et al., 2021; Farrelly et al., 2021; Ishii et al., 2020). A recent review by Lee and Rompolas highlights the heterogeneity found in the limbal stem cells and describes implications for the clinical impact of these findings (Lee and Rompolas, 2022).

YAP has been shown to be critical in the regeneration of the corneal epithelium. We have previously demonstrated mice deficient in YAP (*Yap1*^+/−^) have extremely dystrophic corneas with thinning and keratinization of the corneal epithelium (Kim et al., 2019). Interestingly, loss of the other major mechanotransducer, TAZ, had no impact on the corneal epithelium (Leonard et al., 2023). An inducible *Yap1* knockout mouse was used to determine the role of YAP in corneal regeneration. Deletion of *Yap1* led to reduced numbers of differentiated corneal epithelial cells (K12^+^), demonstrating the importance of YAP to epithelial cells proliferation in both the limbus and central cornea (Kasetti et al., 2016). In corneal epithelial wound healing, there are different courses of regeneration that are dependent on the size of the wound, with limbal epithelial stem cell-dependent (large wounds) and -independent pathways (small wounds). Interestingly, YAP plays a critical role in both forms of corneal epithelial regeneration that led to the formation of cell junctions and assembly of F-actin cytoskeleton, to facilitate a rapid epithelial wound closure (Li et al., 2021b). These data demonstrate the importance of YAP in proliferation and regeneration of corneal epithelial cells, however, the impact of wound biomechanics on YAP signaling and corneal epithelial wound healing have yet to be explored.

### 3.5. Therapeutic strategies that recreate native corneal biomechanics

Multiple questions surround the role of mechanotransduction in ocular surface disease, particularly in limbal stem cell deficiency. It is unclear if there are alterations in the biomechanical properties of the basement membrane, Bowman’s layer or the anterior stroma that affect limbal stem cell homeostasis. One of the major challenges in the development of *in vitro* culture-based limbal stem cell transplants is the loss of self-renewal and long-term proliferative capacity of these cells when transferred *in vivo*. This limitation has markedly limited the potential of regenerative therapies for limbal stem cell deficiency (Altshuler et al., 2023; McKee and Chaudhry, 2017). Typically, limbal stem cells are expanded on amniotic membrane with a tissue stiffness that ranges from 2 to 20 MPa, depending on storage conditions prior to culture (Benson-Martin et al., 2006), a value much greater than the normal anterior basement membrane of the cornea. However, if the donor limbal stem cells for transplantation were cultured on substrates mimicking healthy ECM stiffness and topography, and portions of the healthy ECM were included in the limbal stem cell transplant, that might improve clinical outcomes with decreased failure of the transplanted cells to maintain their stemness. Equally as important, if the recipient ECM has both an abnormal composition and/or exhibits altered tissue stiffness, a limbal stem cell transplant cultured on substrates with normal physiologic stiffness may lose its stemness and terminally differentiate, causing the transplant failure. These are some of the important factors that need to be evaluated and considered as we continue to develop our corneal epithelial transplants for limbal stem cell deficiency.

Recurrent corneal erosion (RCEs) syndrome is a common ophthalmic disorder characterized by poor attachment of corneal epithelial cells to the anterior basement membrane. RCEs are typically associated with a traumatic event or a corneal epithelial basement membrane dystrophy, other corneal dystrophies or degenerations, or prior ocular refractive surgery (Miller et al., 2019). Specifically, multiple proteins, including hemidesmosomes, type IV, VII and XVII collagen and MMPs regulate the adhesion complexes between corneal epithelial cells and their basement membrane (Martin et al., 2021). Upregulation of MMP2 and MMP9 in patients with RCEs results in the cleavage of adhesion complex proteins, including fibronectin and laminin (Martin et al., 2021). However, while treatments for RCE include the use of topical antibiotics, matrix metalloproteinase inhibitors, blood- and amniotic membrane-derived ophthalmic treatments, and corticosteroids, many patients require additional therapies (Miller et al., 2019). Surgical intervention with diamond burr debridement and anterior stromal puncture techniques can improve healing rates in patients with RCE. These surgical interventions likely alter the keratocyte activation state, anterior stromal biochemical composition, and tissue biomechanics, however, very little is known about the effects of these treatments on mechanobiology of the anterior cornea. To explain how these interventions may improve wound healing, a few recent studies have highlighted the impact of tissue stiffness on epithelial cell migration. Mechanosensitive Piezo1 channels of epithelial cells are critical in the polymerization the actin cytoskeleton and cell motility (Jetta et al., 2023). Piezo1 channels became activated when epithelial cells are cultured on stiffer substrates, resulting in thickening and enlargement of F-actin fibers, increasing cell contractility and migration (Jetta et al., 2023). These results suggest that corneal epithelial cell migration via the actin cytoskeleton is dependent on Piezo1 channel signaling, and impairment of Piezo1 signaling could lead to altered epithelial cell migration. The cell-cell and cell-substrate interactions of epithelial cells can also be modulated by tissue stiffness. Importantly, alterations tissue biomechanics can impact epithelial cell polarity, leading to dysfunction in cell migration and attachment to the underlying basement membrane (Martin et al., 2021). Additional studies have identified increased epithelial cell migration on stiffer substrates, directly related to myosin-II activation and establishment of cell polarity (Ng et al., 2012). However, a recent study demonstrated that increased substrate stiffness can decrease epithelial cell motility, potentially delaying wound healing (Choi et al., 2022). One could predict that activation of keratocytes to fibroblasts may lead to increased deposition of type III collagen and upregulating contractile elements to increase tissue stiffness. By inferring from other organ systems, an increase in tissue stiffness may promote increased cellular adhesion to the ECM through integrin clustering and focal adhesion activation (Handorf et al., 2015; Payne et al., 2005). Therefore, an effective treatment for RCEs may require modulation of tissue stiffness, in addition to altering the biochemical composition, to enhance cell attachment to the ECM and facilitate wound healing. However, there is a need to develop a broader understanding of the effects of tissue stiffness on adhesion complex formation between corneal epithelial cells and their basement membrane as means for developing better therapeutic interventions. This guiding strategy may direct additional medical and surgical approaches that alter corneal biomechanics to promote wound healing. Dogs have an analogous condition to RCEs, termed spontaneous chronic corneal epithelial defects (Bentley, 2005; Bentley et al., 2001, 2002; Carter et al., 2007; Murphy et al., 2001), and can thus be utilized as a preclinical model to evaluate therapeutics that alter stromal biomechanics such as corneal CXL.

## 4. Stroma

### 4.1. Anatomy and physiology of the stroma

The composition of stroma serves as a rich environment complete with the biomechanical and topographic variability to support all its resident cells. Like most tissues, water dominates its composition with stability provided by a highly ordered network of collagens, GAGs, and glycoproteins. Type I collagen is most abundant protein, arranged in structural, banded fibrils with a relatively uniform diameter of 25–35 and 50 nm in the central and perilimbal cornea, respectively, depending on the species (Meek and Leonard, 1993). Interfibrillar spacing is relatively constant at 39–67 nm (center to center) in the axial cornea (Meek and Leonard, 1993), then dramatically increases in the perilimbal cornea (Boote et al., 2003; Meek and Knupp, 2015). The collagen fibrils arrange into parallel lamellae running at oblique angles to one another which results in a highly ordered, lattice-like arrangement. The GAGs maintain regular spacing between fibrils. At the limbus, the fibrils develop a concentric, interwoven configuration which strengthens the cornea and maintains its curvature.

Keratocytes reside between collagenous lamellae and are responsible for generating and remodeling their surrounding matrix. These stellate cells extend multiple, extensive dendritic processes to interact with other keratocytes and form an intricate syncytium connected via gap junctions (Nishida et al., 1988). Abundant corneal crystallins (~25–30% of the intracellular soluble protein) including aldehyde dehydrogenase (ALDH1A1) and transketolase (TKT) maintain transparency of the keratocyte cytoplasm. In addition, the dendritic cellular processes of normal keratocytes demonstrate negligible backscatter of light (Jester et al., 1999). The even spacing of these thin keratocytes in a clock-wise circle throughout the stroma further limits light scatter.

### 4.2. Vertebrates demonstrate incredible stromal diversity

**Fig. 10.**
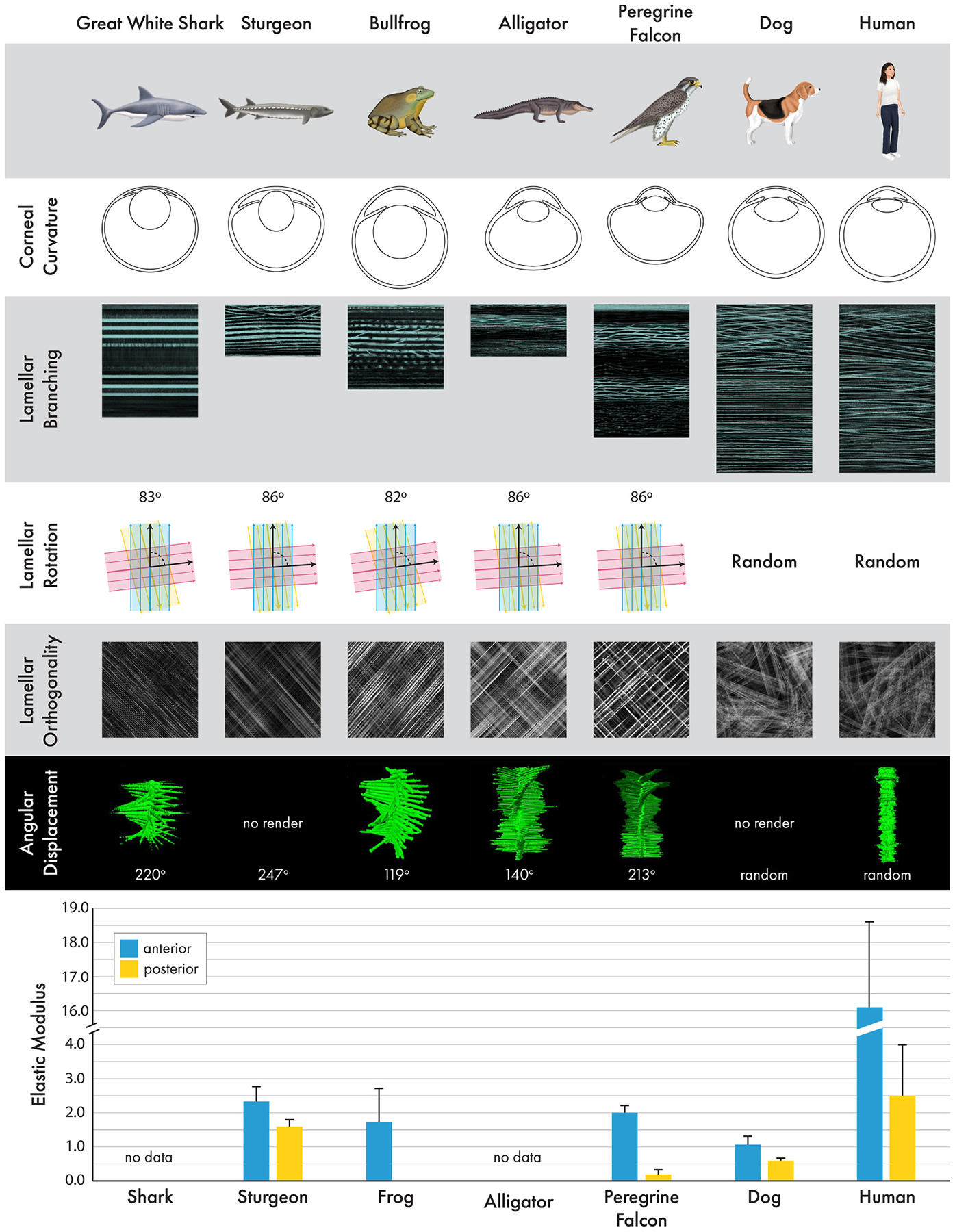
Vertebrates demonstrate diverse corneal shape, lamellar collagen branching and orientation as well as stromal stiffness. Aquatic species such as shark and sturgeon have flat corneas with no refractive power while semi-terrestrial and terrestrial species utilize a curved cornea as a refractive lens. Cross-sectional high-resolution macroscopic images of corneas demonstrate nearly absent branching in the shark and sturgeon, intermediate branching in bullfrog and alligator, and extensive branching in the peregrine falcon. In non-mammalian corneas, nearly perpendicular orthogonal layering of collagen sheets (fish, amphibians and reptiles or ribbons) or ribbons (birds) is observed, which leads to a clockwise chiral-nematic arrangement reminiscent of a cholesteric liquid crystal. By contrast, mammalian corneas demonstrate a random orientation of collagen fiber bundling with diverse interweaving ranging from just anterior (dog) to all but the most posterior cornea (human). In all species, the anterior stroma is stiffer than the posterior stroma. Adapted from Winkler, M., Shoa, G., Tran, S.T., Xie, Y., Thomasy, S., Raghunathan, V. K., Murphy, C., Brown, D.J., Jester, J.V., 2015. A Comparative Study of Vertebrate Corneal Structure: The Evolution of a Refractive Lens. Invest Ophthalmol Vis Sci 56, 2764–2772, copyright Association for Research in Vision and Ophthalmology.

The evolution of the cornea as a refractive lens demonstrates divergence between the mammalian and non-mammalian species as well as changes in complexity between anamniotes and amniotic non-mammalian vertebrates (Fig. 10) (Koudouna et al., 2018; Winkler et al., 2015). In non-mammalian corneas, the stromal collagen is organized in an orthogonal/rotational paradigm similar to chiral-nematic liquid crystals. Fish demonstrate the simplest organization with a structure analogous to plywood. The cornea of the fish is flat with collagen organized in lamellar sheets in a distinct orthogonal/rotational pattern with collagen lamellae interconnected by rare lamellar branches with thin “sutural” fibers present in some species. Amphibians and reptiles also display rotated sheets of collagen, but fiber branching is much more frequent and prominent. In birds, parallel intertwined ribbons of collagen are layered orthogonally with the greatest amount of branching and anastomoses of the non-mammalian corneas. In the posterior cornea of birds and some reptiles, the rotation terminates. By contrast, mammals demonstrate a completely different structural model of highly interconnected collagen bundles and transverse fibers that are randomly oriented.

**Fig. 11.**
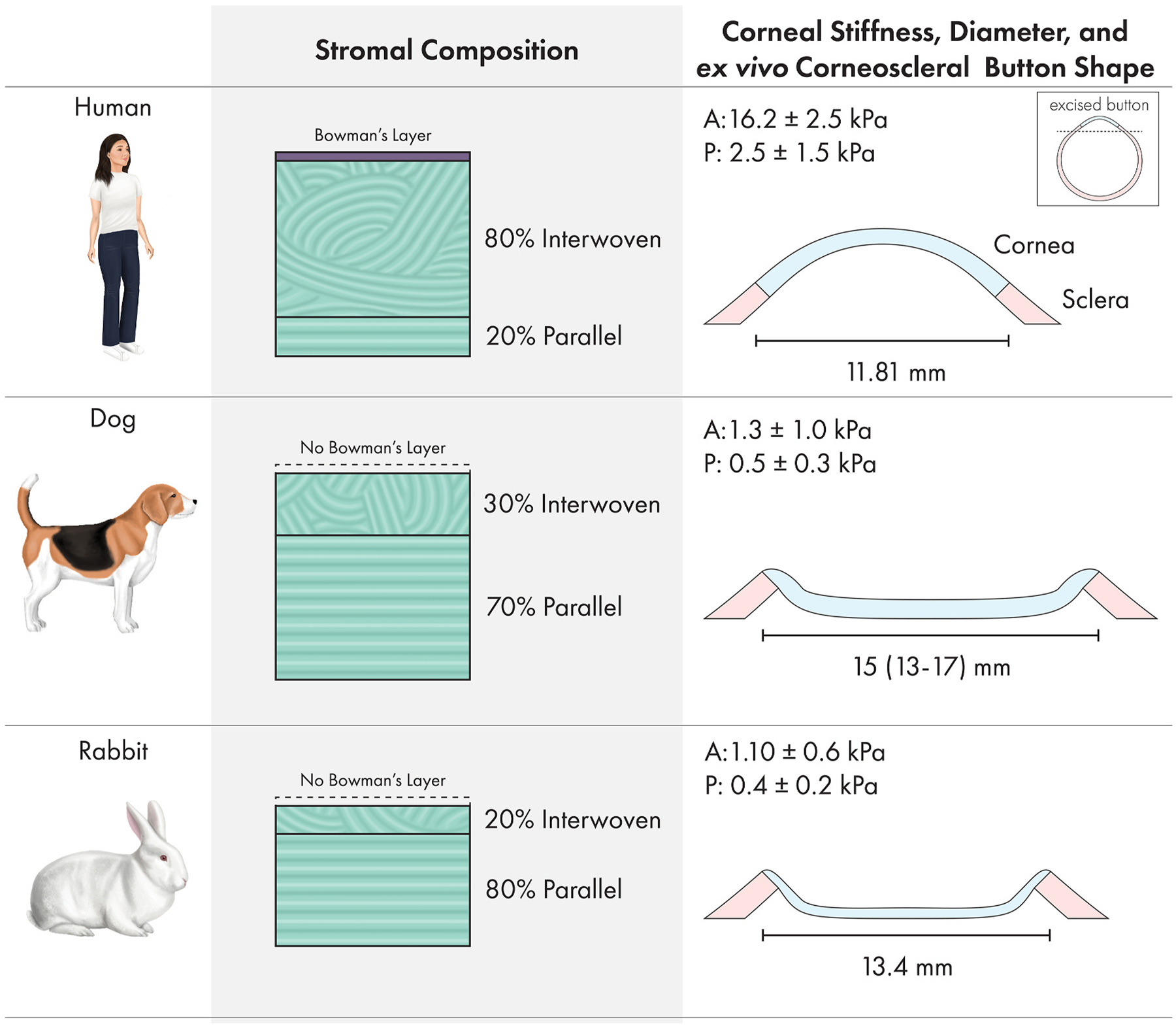
Mammalian corneas exhibit dramatic differences in their stromal collagen intertwining and stiffness. Note the dramatic differences in collagen fiber structure between the human, dog, and rabbit. A Bowman’s layer is present in humans and absent in domestic mammals like dogs and rabbits. These differences are likely responsible for the differences in elastic modulus observed between these three species in the anterior (A) and posterior (P) stroma and likely the reason why an *ex vivo* corneoscleral button harvested from a human maintains its shape while the cornea in a rabbit or dog donor button collapses. Data from (Leonard et al., 2019a; Thomasy et al., 2014, 2016b).

Collagen fiber intertwining in mammalian corneas is most pronounced in the anterior cornea and varies between species. For example, collagen fiber intertwining is present in the anterior 80% of human corneas versus only 10–20% in rabbits and dogs (Leonard et al., 2019a; Thomasy et al., 2014; Winkler et al., 2011). Furthermore, Bowman’s layer provides additional rigidity to the human cornea via collagen fibers that form bow spring-like structures that act like crossbeams in a bridge. These differences in rigidity can be observed in corneoscleral buttons from these three species whereby the human cornea retains its shape while corneal collapse is observed in the other two species (Fig. 11). These biomechanical differences create challenges when translating corneal surgical techniques from human to veterinary patients (Boo et al., 2019).

Herein, we present novel data regarding stromal biomechanics of 3 species, sturgeon, bullfrog, and peregrine falcon, using atomic force microscopy (AFM), a nanoindentation method for measuring elastic modulus, generated with previously described methods (Fig. 10) (Last et al., 2012; Leonard et al., 2019a; Morgan et al., 2014). Atomic force microscopy offers a number of advantages to other methods of assessing tissue stiffness as detailed in the review by McKee and colleagues (McKee et al., 2011). We highlight that in all vertebrate species, the more intertwined collagen of the anterior stroma is stiffer than the lamellar sheets, ribbons, or bundles present the posterior cornea. The non-mammalian species and mammals without a Bowman’s layer have markedly softer corneas than humans ranging from ~1 to 3 kPa for the anterior stroma and ~0.25–1.5 kPa for the posterior stroma (Figs. 10 and 11). By contrast, humans have a markedly stiffer cornea ranging from ~15 to 250 and ~5–100 kPa in the anterior and posterior stroma, respectively (Dias et al., 2013; Last et al., 2012; Leonard et al., 2019a). The elastic modulus of the corneal stroma in nonhuman primates has not been reported and represents an important opportunity given its close anatomic similarity to the that of humans (Casanova et al., 2022; Meek and Leonard, 1993). While the porcine corneal surface has been measured with AFM (Li et al., 2020a), stromal measurements have not been performed. The biomechanical properties of the chicken cornea, an important species for the study of corneal development, are also lacking. These biomechanical differences must be considered when generating biologically relevant substrates to study the impact of stiffness on keratocyte behavior in various species as well as choosing an ideal animal model to study corneal wound healing and novel therapeutics to optimize it.

### 4.3. Stromal wound healing

**Fig. 12.**
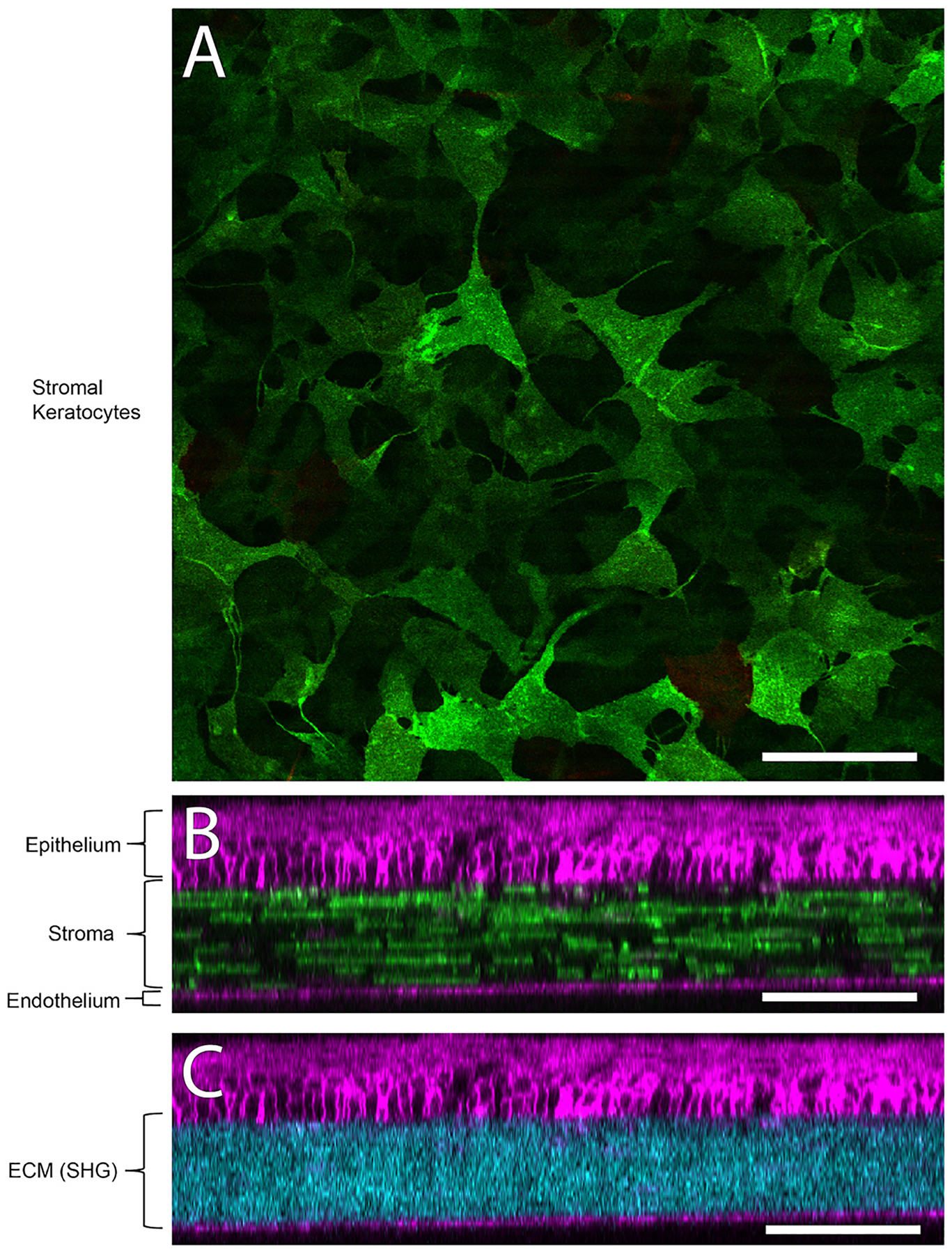
Two-photon and second-harmonic imaging facilitates monitoring of corneal cells and extracellular matrix *in vivo*. High magnification en face image of keratocytes in a normal mouse cornea at 80 μm depth **(A)**. A cross-sectional view of the epithelium, stroma and endothelium are shown with keratocytes visible in green **(B)** or stromal extracellular matrix in cyan **(C)**. A globally expressed, membrane-localized fluorescent reporter (*Rosa2*6-mTmG) was used to visualize cell morphology *in vivo*. The stromal ECM is visualized by Second-Harmonic Generation and depicted in cyan. Scale bar represents 100 μm. Image courtesy of Dr. Panteleimon Rompolas, Departments of Dermatology and Ophthalmology, University of Pennsylvania Perelman School of Medicine.

Corneal wound healing is a complex process integrating soluble cytoactive factors with biophysical stimuli within the matrix of the injury. Epithelial and stromal wound healing are intimately connected with injured epithelial cells secreting TGFβ, platelet-derived growth factor (PDGF), and fibroblast growth factor (FGF) that initiate the transition of quiescent keratocytes to activated fibroblasts and myofibroblasts (Wilson et al., 2017). Thus, keratocyte-fibroblast-myofibroblast (KFM) transformation and the interactions between these activated stromal cells and the ECM that they produce and remodel are central to corneal stromal repair (Myrna et al., 2009). Following corneal injury, fibronectin is initially assembled by platelets or fibroblasts and myofibroblasts. Contraction of stromal cells stretches this initial fibronectin matrix thus inducing fibronectin fibrillogenesis and subsequent collagen fibril deposition (Bhattacharyya et al., 2017; Pot et al., 2023; Suda et al., 1981; Zhong et al., 1998). Recent advances in two-photon microscopy and second harmonic imaging by the Rompolas laboratory permit both identification of keratocytes and ECM in a murine model which can be used to investigate stromal wound healing (Fig. 12). Mohan and coauthors recently summarized the known literature regarding corneal stromal repair emphasizing the role of cytoactive factors in the process and the therapeutics targeting them (Mohan et al., 2022).

**Fig. 13.**
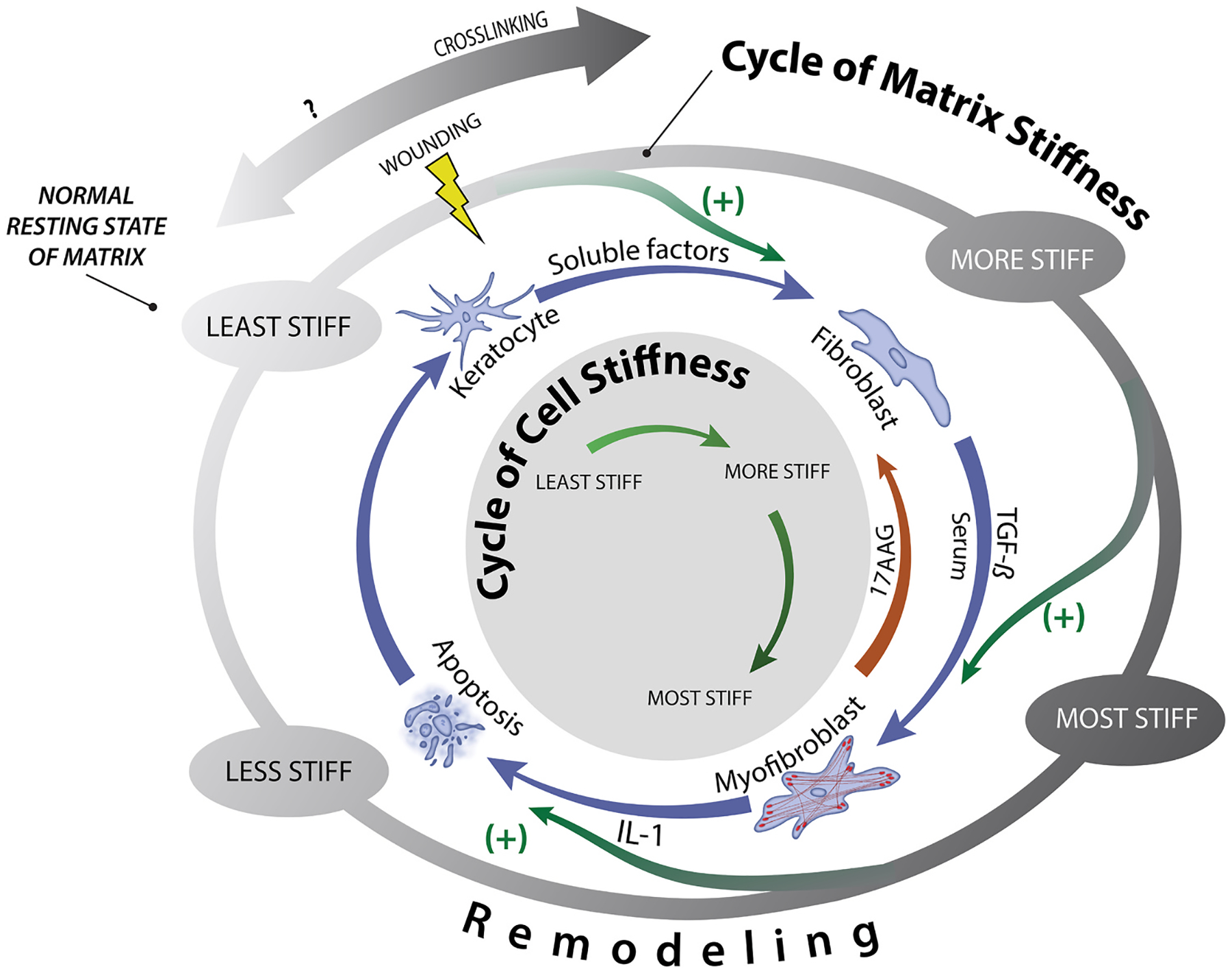
Dynamic interactions between the matrix and cellular stiffness during corneal wound healing. We have demonstrated that the intrinsic stiffness of the corneal stroma undergoes dynamic changes in stiffness throughout wound healing in a rabbit PTK model and that transformation of quiescent keratocytes to contractile myofibroblasts is facilitated by both soluble factors (e.g. TGFβ) and substratum stiffness *in vitro* (Dreier et al., 2013; Raghunathan et al., 2017). By contrast, 17AAG can revert fibroblasts and myofibroblasts to a keratocyte phenotype *in vitro* (Raghunathan et al., 2021). Furthermore, we have demonstrated that crosslinking stiffens the stroma resulting in myofibroblast persistence and increased stromal haze in a rabbit model *in vivo* (Moore et al., 2023). *Novel therapeutics that target stromal softening may facilitate return to a more homeostatic corneal matrix and cell population.*

Biomechanical forces and cell-ECM interactions are also critical to corneal stromal wound healing. We demonstrated dynamic changes in elastic modulus in the anterior corneal stroma following phototherapeutic keratectomy (PTK) in rabbits initiated by edema and inflammation, and subsequently followed by the formation of stromal haze and myofibroblast entry in the wound (Raghunathan et al., 2017). Importantly, stromal stiffening preceded the appearance of myofibroblasts, suggesting that alterations in stromal biomechanics may be an initiating factor in KFM transformation (Fig. 13) (Dreier et al., 2013). Using multiphoton fluorescence and second harmonic generation imaging, Kivanany and colleagues characterized cell-ECM interactions within a stromal wound using a rabbit PTK model (Kivanany et al., 2018a). Initially, fibroblasts co-aligned within the stromal lamellae to facilitate the migration below the ablation site. Next, myofibroblasts on top of the ablation site deposited fibronectin in a random orientation, a feature that was associated with marked stromal haze. Within this region, fibroblasts then deposited collagen fibers in alignment with their intracellular stress fibers and subsequently remodeled this collagen into more transparent stromal lamellae with restoration of keratan sulfate expression. Together, these studies provide a detailed temporal, biomechanical, and spatial assessment of stromal wound healing following PTK that likely translate to similar injuries such as lamellar keratectomy (Kivanany et al., 2016). These studies also have important implications for corneal wound healing in patients receiving corneal CXL with riboflavin, a treatment that results in long-term stiffening of the anterior stroma, which we will discuss in 4.10 Therapeutics that may alter stromal biomechanics.

By contrast, alkali burns are a much more severe corneal injury that results in damage to all three corneal cell types, tissue necrosis, neutrophil infiltration, activation of resident macrophages, and thus altered stromal biomechanics (Sprogyte et al., 2023). Using optical coherence elastography (OCE), Young’s modulus using a modified Rayleigh-Lamb wave model in mice significantly increased from a baseline of ~1–~2 kPa on days 7 and 14 following alkali-induced injury (Mekonnen et al., 2022). The changes at 7 days post-injury are most likely due to destruction of proteoglycans resulting in increased collagen fibril density while at 14 days post-injury there are additional contributions from inflammatory infiltrate, neovascularization and fibrosis from the healing process (Mekonnen et al., 2022). These studies demonstrate that regardless of the type of injury to the cornea, alterations in the biomechanical properties of the corneal stroma are fundamental attributes modulated by injury and may have implication on cell behavior. Nevertheless, since the mechanism of injury between a refractive surgery and chemical burn differ significantly, the changes to the biochemical composition of the cornea may also be different. As such, the receptors that may be involved in sensing alterations within the microenvironment may differ and thus elicit a differential response. Thus, context dependent investigations may be essential to critically assess the utility and values of moduli obtained in these studies (Yeganegi et al., 2023).

### 4.4. Keratocytes respond to changes in stiffness

**Fig. 14.**
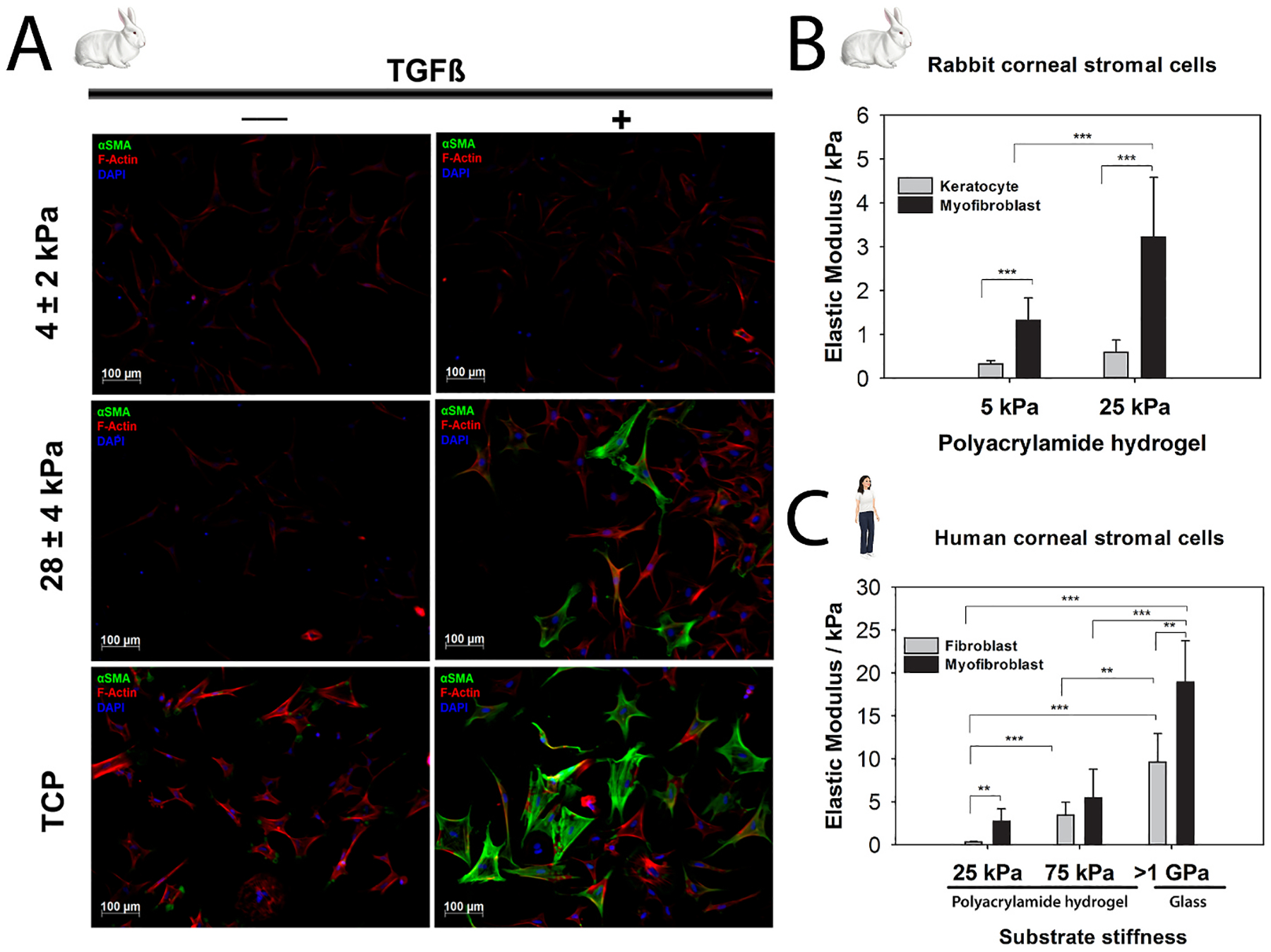
Compliant substrates limit the transformation to the myofibroblast phenotype and modulate stromal cell stiffness. Rabbit corneal fibroblasts were cultured in the absence or presence of TGFβ1 on substrates with varying compliance then fixed and immunohistochemical stains performed for α-smooth muscle actin (αSMA, green), F-actin (red), and 40,6-diamidino-2-phenylindole (DAPI; blue). Most cells on TCP displayed strong staining for αSMA when exposed to TGFβ1, consistent with a myofibroblast phenotype. By contrast, few cells (on 28-kPa gels) or no cells (on 4-kPa gels) stained positive for αSMA, even with TGFβ1 treatment; F-actin demonstrated increased stress fiber formation on TCP versus the compliant substrates **(A)**. Rabbit **(B)** and human **(C)** corneal stromal cells were stiffer on substrates mimicking the elastic modulus of native stroma in health and disease; TGFβ1 treatment further increased cell stiffness. Adapted from Dreier, B., Thomasy, S.M., Mendonsa, R., Raghunathan, V.K., Russell, P., Murphy, C.J., 2013. Substratum compliance modulates corneal fibroblast to myofibroblast transformation. Invest Ophthalmol Vis Sci 54, 5901–5907, copyright Association for Research in Vision and Ophthalmology and Raghunathan, V.K., Thomasy, S.M., Strøm, P., Yañez-Soto, B., Garland, S.P., Sermeno, J., Reilly, C.M., Murphy, C.J., 2017. Tissue and cellular biomechanics during corneal wound injury and repair. Acta Biomater 58, 291–301.

Stromal stiffness markedly impacts keratocyte behavior in both health and disease, particularly during corneal injury and repair (Yang et al., 2022). Fabricating two-dimensional (2D) substrates of varying stiffness facilitates investigations of substratum stiffness independent of topography and composition (Dreier et al., 2013; Thomasy et al., 2018). Utilizing polyacrylamide hydrogels, we were the first to identify that substratum stiffness modulated TGFβ1-induced KFM transformation with softer substrates (4 kPa) stabilizing the keratocyte phenotype even in the presence of TGFβ1 (Fig. 14) (Dreier et al., 2013). Maruri and colleagues further demonstrated that a stiff microenvironment alone was insufficient to stimulate KFM transformation (Maruri et al., 2020). However, in the presence of TGFβ1, stromal cells on a stiff substrate (11 kPa) exhibited broader morphologies, abundant stress fibers, increased α-smooth muscle actin (αSMA) expression, and exerted larger tractional forces versus those on a soft substrate (1 kPa) (Maruri et al., 2020). Using PDMS substrates, Chen and co-authors examined the interplay between inflammation and stiffness on maintenance of a keratocyte phenotype (Chen et al., 2023b). They demonstrated that the inflammatory factor, interleukin-1β (IL-1β), promoted keratocyte markers of a transformed keratocyte cell line on softer substrates (~130 kPa) but not stiffer PDMS (~2 MPa) or tissue culture plastic (TCP, ~1 GPa). Furthermore, macrophages on stiff substrates expressed greater amounts of IL-1β and tumor necrosis factor-α (TNFα) (Chen et al., 2023b). We demonstrated that substratum stiffness modulates stromal cellular response to the cytoskeletal disruptor, latrunculin B, and the antifibrotic, hepatocyte growth factor (Miyagi et al., 2018; Thomasy et al., 2013). Others have demonstrated similar stiffness-dependent responses of corneal stromal cells to therapeutic agents including hydroxycamptothecin and PDGF-BB (Chen et al., 2023a; Lam et al., 2023). These responses can be unpredictable as observed with the antifibrotic, hydroxycamptothecin (Chen et al., 2023a). This compound increased mRNA and protein expression of keratocyte markers but did not prevent the upregulation of αSMA expression when stimulated with TGFβ1 on both soft and stiff substrates. It is thus critical to consider corneal stromal cell responses to cytoactive factors and therapeutic agents within the context of biologically relevant substratum cues, including stiffness.

Substratum stiffness can also modulate cellular physical reprogramming of corneal keratocytes into stem-like cells and adherent 3D spheroid cultures without transcription factors (Li et al., 2021a). Li and co-authors demonstrated that PDMS substrates with hydrophobic and soft properties were more effective at increasing the expression of stem cell markers in keratocytes and initiating reprogramming than ultralow attachment plates, a common method to form suspended 3D spheroids (Li et al., 2021a). This observation was corroborated in another study where by human induced pluripotent stem cells (h-iPSCs) were more likely to retain their phenotype on soft matrigel-coated PDMS (4 kPa) while differentiation to corneal stromal cells occurred on stiffer matrigel-coated TCP (Zhang et al., 2023a). The h-iPSCs grown on the PDMS substrate where successfully seeded onto a multi-layered biomimetic collagen substrate that remained transparent with high cell survival (Zhang et al., 2023a). Cell-derived matrices are a compelling alternative to conventional biomimetic scaffolds as they more closely recapitulate the native ECM. Human pluripotent stem cells cultured on decellularized fibroblast-derived matrices (FDMs) retained epithelial and pluripotent features on crosslinked FDMs while exhibiting adhesive, proliferative and EMT-inducing characteristics on the uncrosslinked FDMs (Kim et al., 2018). Thus, cell-derived matrices may be useful in further tuning the transformation of keratocytes to stem cells for producing tissue-engineered implants for clinical use. Further work is indicated to determine the underlying mechanotransduction pathways initiating these changes in stem cell fate.

As animal models of corneal diseases are generated with stromal pathology, it is critical to assess the biomechanical changes to the stroma in addition to characterizing morphological alterations. Knockout of collagen XII in mice resulted in decreased interfibrillar space, disorganized lamellae, and abnormal keratocyte organization within the corneal stroma demonstrating the importance of this understudied collagen in maintaining normal stromal structure and function (Sun et al., 2020). Another study demonstrated that these collagen XII-deficient mice had a decrease in stiffness using three different methods for assessing corneal mechanics – Brillouin microscopy, air-coupled OCE, and heartbeat OCE (Nair et al., 2022). Mice homozygous for a Zfp469 mutation, a model for brittle cornea syndrome, demonstrated marked central and peripheral corneal stromal thinning, smaller diameter collagen fibrils, and decreased Col1a1 and Col1a2 expression suggesting reduced biomechanical strength although elastic modulus was not directly measured (Stanton et al., 2021). Finally, murine *Col5a1* heterozygotes, a common model for Ehlers-Danlos syndrome, demonstrated thin corneas, abnormal collagen fibrils, an increased elastic modulus with OCE, and stronger relaxation under stress testing (Kling et al., 2021). Changes in the viscoelastic properties permit these thin corneas to maintain a normal shape. In summary, these studies demonstrate the importance of assessing the impact of altered stromal composition on the biomechanical properties of cornea in animal models of stromal diseases.

### 4.5. Topographic cues modulate keratocyte behavior

**Fig. 15.**
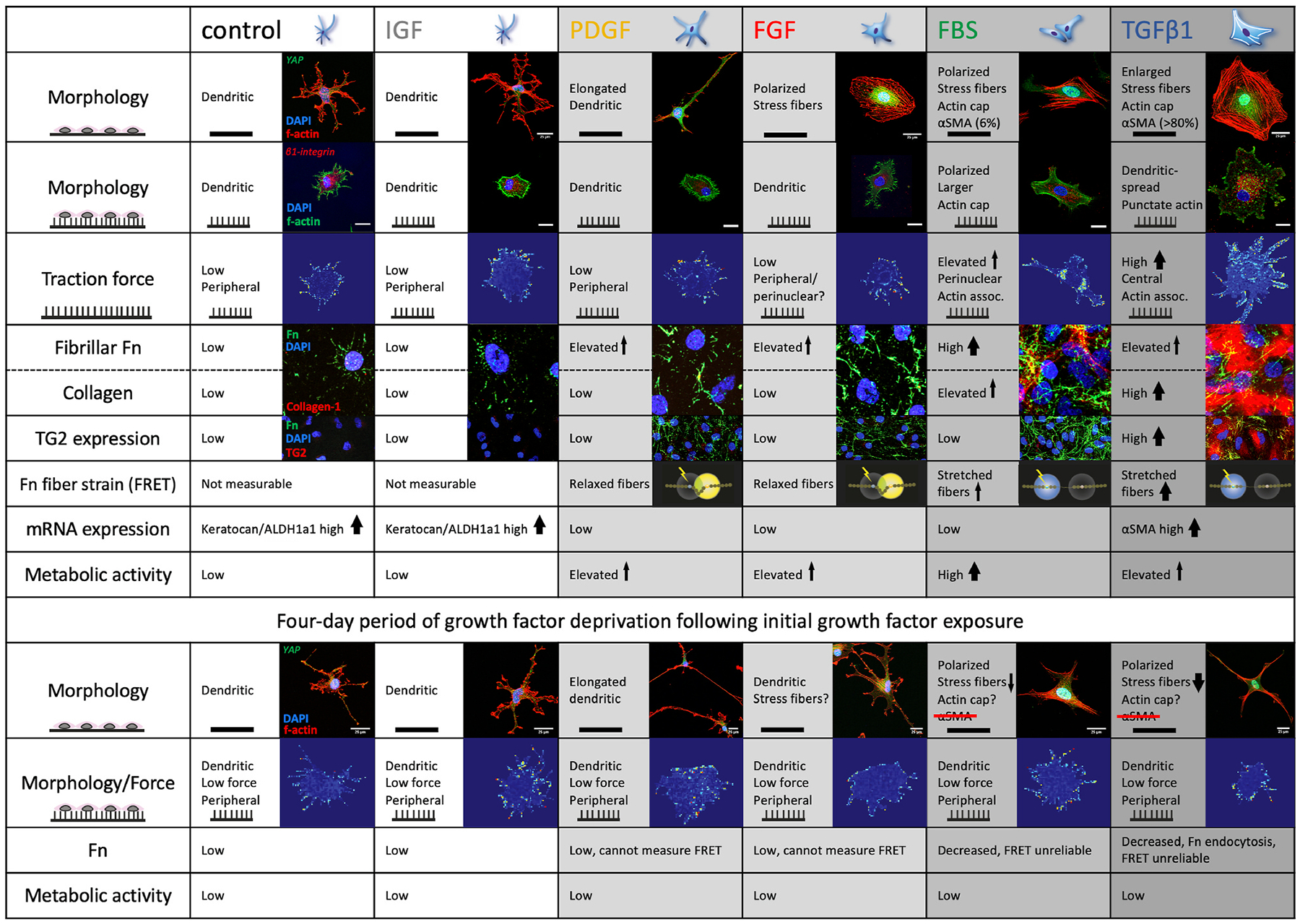
Growth factors and their subsequent deprivation initiate distinct cell morphologies, behaviors, and matrix secretion in keratocytes. Cells treated with IGF demonstrated similar morphology to control keratocytes with low, peripheral traction force, low fibrillar fibronectin (Fn) and collagen deposition, absent Fn fiber strain, low expression of transglutaminase 2 (TG2), high expression of keratocan and ALDH1a1, and low metabolic activity. Withdrawal of IGF did not alter keratocyte morphology, force, fibronectin fiber strain and metabolic activity. By contrast, keratocytes treated with PDGF or FGF demonstrated a proliferative, metabolically active, low contractility phenotype with contact-guided migration and formation of a fibrillar fibronectin matrix. The FBS- or TGFβ1-treated keratocytes displayed extensive stress fibers, an actin cap, and high traction force consistent with a (myo)fibroblast phenotype and deposited the densest Fn and collagen matrix with marked contraction and extensive reorganization of their surrounding ECM. Withdrawal of PDGF, FGF, FBS or TGFβ1 returned the corneal cells to a keratocyte-like phenotype with low, peripheral traction force, low to decreased fibrillar Fn and collagen deposition, and absent to low Fn fiber strain, and low metabolic activity. Reprinted with permission from Pot, S.A., Lin, Z., Shiu, J., Benn, M.C., Vogel, V., 2023. Growth factors and mechano-regulated reciprocal crosstalk with extracellular matrix tune the keratocyte-fibroblast/myofibroblast transition. Sci Rep 13, 11350, CC04.0.

Matrix topography in the local microenvironment of keratocytes has a similarly profound impact on cellular behavior as stiffness under physiological and pathological conditions (Flemming et al., 1999; Petroll et al., 2020). Fabricated substrates with anisotropic nano- and microscale features with variable dimensions and features have been used to investigate the biological responses of corneal stromal cells (Karamichos et al., 2014; Liliensiek et al., 2006; Myrna et al., 2012; Pot et al., 2010; Teixeira et al., 2004; Vrana et al., 2008). Indeed, corneal fibroblast morphology (Fraser et al., 2008; Pot et al., 2010), proliferation (Liliensiek et al., 2006), alignment (Fraser et al., 2008; Pot et al., 2010; Teixeira et al., 2004), migration (Pot et al., 2010), differentiation (Karamichos et al., 2014; Myrna et al., 2012), and ECM secretion (Karamichos et al., 2014; Vrana et al., 2008) all dramatically differ on ridged and grooved versus planar surfaces. Strikingly, corneal stromal cells align parallel to the ridges and grooves with a dramatically elongated phenotype akin to that observed in corneal lamellae *in vivo* (Pot et al., 2010; Teixeira et al., 2004). Fibroblasts and myofibroblasts migrated parallel to surface ridges >1000 nm but lacked directional guidance on ridges <1000 nm and planar substrates while keratocytes were immobile on all substrates (Pot et al., 2010). Pot and colleagues recently demonstrated that keratocytes exposed to FGF maintained a dendritic phenotype when grown on nanopillar arrays but differentiated to a fibroblast-like phenotype with polarization and stress fibers on glass revealing that topographic substrates also modulate responsivity to cytoactive factors (Fig. 15) (Pot et al., 2023). For a comprehensive summary of methods to generate substrates that mimic corneal ECM topography and studies of their effects, we refer the reader to the review by Petroll and colleagues (Petroll et al., 2020).

The organization of type I collagen into parallel fibrils is a key feature of transparency within the corneal stroma. As such, the impact of aligned collagen fibrils and key cytoactive factors on corneal stromal cell behavior has been an important subject of recent study (Kivanany et al., 2018b; Lam et al., 2023). Substrates of aligned collagen can be generated by spin coating (Saeidi et al., 2011), microfluidic deposition (Lee et al., 2006), molecular crowding (Saeidi et al., 2012), or magnetic field (Torbet et al., 2007) approaches. Kivany and colleagues demonstrated orientation of keratocytes parallel to aligned collagen fibrils increased co-alignment in the presence of PDGF or TGFβ (Kivanany et al., 2018b). Simultaneous exposure of keratocytes to fibronectin-coated aligned collagen fibrils and PDGF-BB resulted in an elongated cell morphology, reduced stress fiber formation and αSMA expression indicating retention of the keratocyte phenotype (Lam et al., 2023). Lam and co-authors further demonstrated that <10% of cells expressed αSMA on fibronectin-coated aligned collagen matrices while adsorbed fibronectin on glass stimulated KFM transformation in ~30% of keratocytes (Lam et al., 2023). These results provide further evidence that keratocyte behavior is influenced by the complex biophysical cues within its environment and are thus important to include when assessing responses not only to cytoactive factors but also to therapeutic agents.

### 4.6. Keratocytes respond to mechanical stretch

The response of corneal fibroblasts to cyclic mechanical stretch has been most extensively studied within the context of keratoconus. Akoto and colleagues examined the transcriptome of healthy and keratoconic corneal fibroblasts under pathophysiological cyclic mechanical stretch (15%) and treatment with TGFβ1 (Akoto et al., 2023). Pathways enriched in the keratoconic cells included ECM degradation, collagen fibril organization, cytoskeletal structure organization, inflammatory response, apoptosis, and Wnt signaling suggesting that these cells retained a gene expression profile *in vitro* relevant to the pathogenesis of keratoconus. Genes induced by cyclic mechanical stretch in the keratoconic fibroblasts were *OBSCN*, *HDAC5*, *AK4*, and *ITGA10* while *CLU* and *F2RL1* were upregulated by cyclic stretch and TGFβ1. Experiments suggest that corneal fibroblasts exposed to small- (5%) and large-scale (15%) mechanical stretch alter their MMP and tissue inhibitors of metalloproteinase (TIMP) expression to promote ECM synthesis and degradation, respectively. In keratoconic fibroblasts, interleukin-6 (IL-6) mediated induction of MMP expression by cyclic mechanical stretch (10%) (Du et al., 2017). Furthermore, large-scale (12%) mechanical stretch downregulated the lysyl oxidase (LOX) expression in stromal fibroblasts from keratoconic patients and treatment with prostaglandin E_2_ resulted in further inhibition (Lan et al., 2017). Given that LOX cross-links collagen fibrils, this suggests a pathological feedback loop whereby corneal structural instability results in further loss of cohesion between collagen fibrils in keratoconus.

In a more recent clinical study, thickness and curvature were shown as major determinants in corneal stress distribution as keratoconus progresses (Roberts et al., 2023). This study showed that in normal corneas, the greatest stress was in the thinnest regions. However, the region of maximal stress shifted to regions with greatest curvature instead of greatest thinness in keratoconic eyes. This suggests that the severity of keratoconus parallels the biomechanical cycle of decompensation where there is a repeating cycle of increased strain, stress redistribution, and subsequent focal steepening and thinning (Ambrósio et al., 2017, 2023; Roberts and Dupps, 2014). Notwithstanding corneal thickness and curvature, the severity of biomechanical characteristics of keratoconic groups were stratified using stress-strain index measurements through correlations with age, biomechanically corrected intraocular pressure, and central corneal thickness (Padmanabhan et al., 2022). These studies also reveal that an asymmetry exists in biomechanical properties determined clinically, using Pentacam HR and Corvis ST, when comparing the two eyes of keratoconic patients and this asymmetry has been posited to be an early predictor of disease progression (Xian et al., 2023).

In aggregate, these results demonstrate changes in stress and strain within the cornea are dynamic in response to genetic, biochemical, and biophysical stimuli both in homeostasis and disease. Thus, we infer that the complex interactions between mechanical instability and biological wound repair response are intrinsic to the pathogenesis of keratoconus and suggest that investigations of therapeutics that address both are warranted.

### 4.7. Corneal stromal cells interact richly with their matrix

**Fig. 16.**
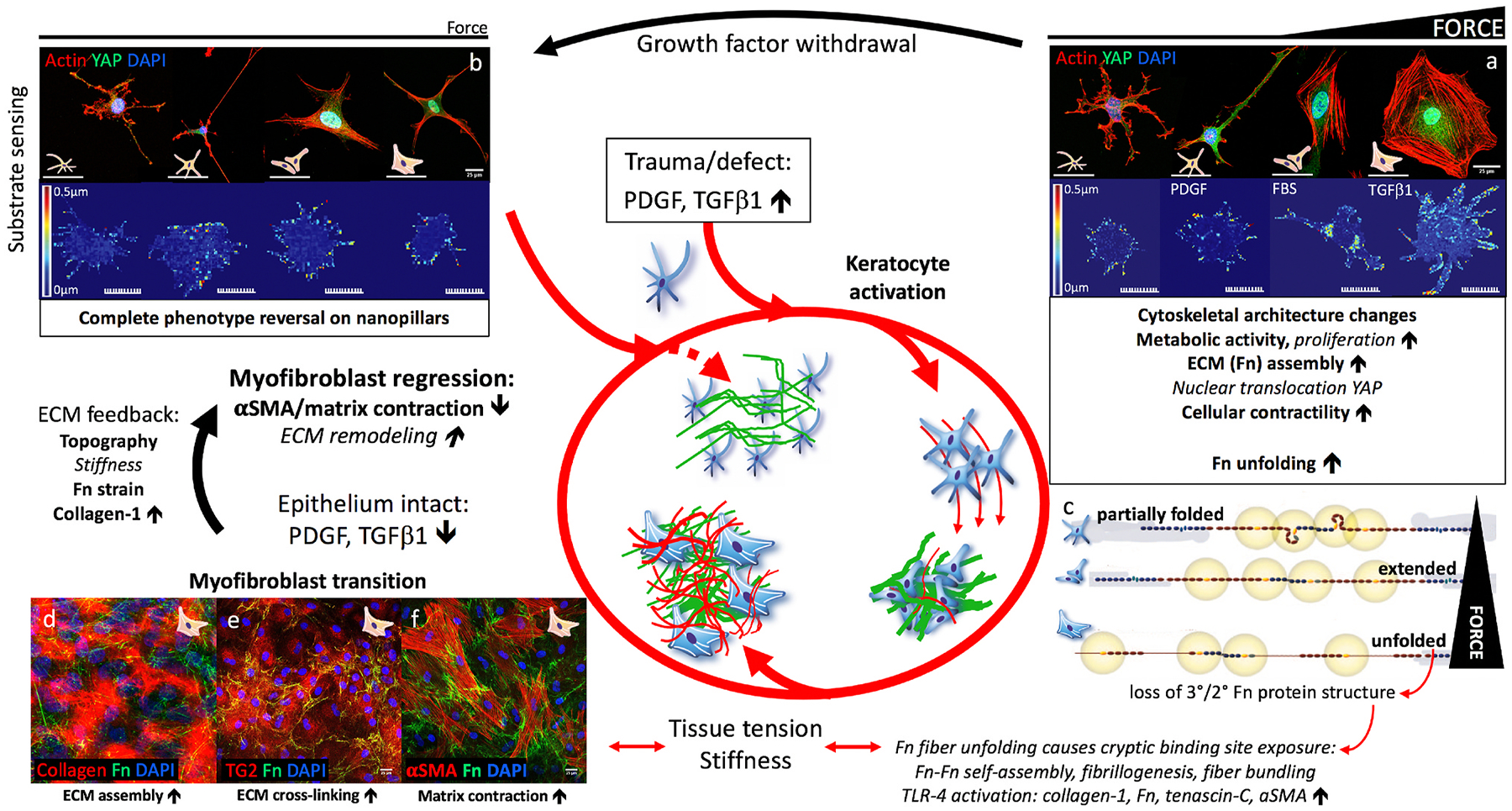
Intimate fibrosis and remodeling feedback loop between corneal stromal cells and their matrix. The damaged corneal epithelium induces release of soluble factors, notably PDGF and TGFβ1, leading to keratocyte activation. **(A)** Activated keratocytes (from left to right: control keratocyte, PDGF-primed fibroblast, FBS-primed fibroblast, TGFβ1-primed myofibroblast) show changes in cell morphology, increased metabolic activity, ECM assembly, contractility, and fibronectin (Fn) fiber unfolding. **(B)** Growth factor removal from cell culture was used to mimic decreasing stromal growth factor amounts following epithelial and basement membrane healing. Substrate type contributed to the completeness of cell phenotype reversal (compare **B** to **A**), such that substrate sensing by cells facilitates their phenotype development. **(C)** Fluorescence Resonance Energy Transfer (FRET) experiments demonstrated that forces generated by cells translate into changes in mechanical matrix strain (Fn unfolding). The most contractile phenotypes, TGFβ1-and FBS and (myo)fibroblasts, stretched Fn fibers within the ECM such that partial secondary/tertiary Fn protein structure loss occurred. This Fn fiber unfolding exposes cryptic Fn-Fn self-assembly sites, accelerating Fn fibrillogenesis, cross-linking and fiber bundling, and stabilization of the early Fn matrix. This Fn fiber unfolding exposes a cryptic Toll-like receptor (TLR) 4 activating site on Fn’s ED-A domain, resulting in TLR activation, and subsequent TGFβ, tenascin-C (TNC), Fn, and collagen-1 gene expression. Cell generated force induced Fn fiber stretching promotes Fn fibrillogenesis with subsequent assembly of a collagen matrix. Increased tissue stresses and tension from cell proliferation, contractility, and matrix assembly drives profibrotic gene expression (incl. αSMA, collagen, TNC), and thus myofibroblast transition and the deposition of a contracted, dense, crosslinked collagen-1-rich matrix **(D**–**F)**, in a self-amplifying manner. Finally, decreasing PDGF and TGFβ1 concentrations following epithelial and basement membrane healing, together with normalizing ECM properties, including tissue specific matrix topography, stiffness, and collagen fiber composition, regulates the disappearance of myofibroblasts from wound sites. Thus, an ECM niche supportive of homeostasis and regenerative remodeling is created. Experimental results (bold) from Pot et al., (2023) with published literature (italics). Reprinted with permission from Pot, S.A., Lin, Z., Shiu, J., Benn, M.C., Vogel, V., 2023. Growth factors and mechano-regulated reciprocal crosstalk with extracellular matrix tune the keratocyte-fibroblast/myofibroblast transition. Sci Rep 13, 11350, CC-BY-NC-ND 4.0.

Cell-derived matrices offer the opportunity to determine how variables alter the cellular production of ECM as well as how cells deposited on this ECM interact with it. Brittle cornea syndrome is a rare disease defined by extreme thinning of the cornea and sclera due to loss-of-function mutations in *ZNF469* or *PRDM5* (Dhooge et al., 2021). The ECM produced by primary keratocytes from mice homozygous for a *Zfn469* mutation had significantly less collagen I deposition than matrix from wildtype keratocytes and could thus serve as an *in vitro* model to further study this condition (Stanton et al., 2021). Pot and colleagues examined cellular morphology, force generation and ECM assembly in keratocytes exposed to and withdrawn from critical growth factors in corneal maintenance and wound healing using 2D cell culture, nanopillar cellular force mapping and a FRET-labeled fibronectin tension probe (Pot et al., 2023). Keratocytes treated with insulin growth factor-1 (IGF-1) retained a dendritic phenotype, exerted low traction force, and produced little collagen or fibronectin matrix consistent with its role in maintenance of normal keratocyte networks. The PDGF-treated keratocytes displayed a proliferative, metabolically active, low contractility phenotype with contact-guided migration and formation of a fibrillar fibronectin matrix in order to repopulate the stromal wound and initiate repair following epithelial damage. By contrast, the FBS- and TGFβ1-treated keratocytes displayed extensive stress fibers, an actin cap, high traction force consistent with a myofibroblast phenotype and deposited the densest fibronectin and collagen matrix with marked contraction and extensive reorganization of their surrounding ECM critical for wound closure. Following a four-day withdrawal of each growth factor after the initial exposure, all cells exhibited a dendritic morphology with low peripheral traction force when grown on the nanopillars, low metabolic activity, and low or decreased fibronectin deposition. The effects of each of these growth factors and their withdrawal are summarized in Fig. 15. The authors also examined how a dense collagen matrix with tense, stretched fibronectin fibers deposited by myofibroblasts impacted the behavior of keratocytes seeded onto this fibrotic ECM. Strikingly, a decrease in KFM transformation was observed on these scaffolds mimicking scarred cornea even in the presence of TGFβ1, suggesting a matrix-induced mechanosensory feedback loop towards the keratocyte phenotype. In summary, this study advances our understanding of corneal wound healing by demonstrating that ECM composition dominated soluble factor signaling in driving KFM transformation and scar tissue ECM promotes regeneration rather than fibrosis using biologically relevant cell culture models (Fig. 16) (Pot et al., 2023).

Engineering three-dimensional (3D) corneal stromal substrates that recapitulate the structure and function of native tissue is critical for *in vitro* modeling as well as *in vivo* regeneration. Mukhey and co-authors exploited corneal stromal stem cells seeded in tethered collagen gels to create Real Architecture for 3D Tissues (RAFT) tissue equivalents with tunable structural properties (Mukhey et al., 2018). Importantly, highly organized collagen-initiated differentiation of stromal stem cells into keratocytes, as indicated by loss of *PAX6* expression. Chen and colleagues describe a novel electro-compacted collagen method to construct an orthogonal 3D corneal stromal model whereby the corneal fibroblasts aligned to the collagen fibrils and increased their expression of keratocyte markers (Chen et al., 2020a). These corneal models have the potential not only to serve as stromal replacements *in vivo* but also are an exciting option for studying corneal stromal cell behavior within a biologically relevant 3D matrix. They also demonstrate many of the most critical features of bioengineered stromal equivalents - oriented collagen I fibrils, a change in fibril orientation between two planes, high transparency, an elastic modulus similar to that of the native tissue, and the ability of corneal stromal cells to colonize it (Tidu et al., 2020). For an in-depth discussion of collagen-based implants for tissue bioengineering of the corneal stroma, we direct the reader to the review by Tidu and co-authors (Tidu et al., 2020).

Corneal organoids further offer a superb 3D model to study how mutations, deficiency or overexpression of key corneal genes impact cell-matrix interactions (Foster et al., 2017). Foster and coauthors optimized corneal organoid production from iPSCs using initial neural and retinal induction and maturation programs followed by corneal selection and maturation (Foster et al., 2017). The mature corneal organoids demonstrated collagen lamellae with three cell subtypes that expressed specific epithelial, stromal, and endothelial markers. Maiti and colleagues compared the transcriptome of human donor corneas with 4-month-old human corneal organoids demonstrating that stromal cells dominated the adult corneas while epithelial and endothelial-like cells were the predominant cell type in the organoids (Maiti et al., 2022). In aggregate, the paucity of studies using corneal organoids represents an opportunity to further define the roles of critical keratocyte genes within a biologically relevant matrix.

### 4.8. Keratocyte mechanotransduction

Keratocytes communicate through an interconnected syncytium and transient plasma membrane disruptions (TPMDs) play a key role. Physiological mechanical loads on cells create micro-tears in the cell membrane, termed TPMDs, which facilitate molecular flux across cell membranes and initiate mechanotransduction (Hagan et al., 2021). Chen and co-authors demonstrated that mechanical manipulation of single keratocytes initiated TPMD-induced calcium waves in adjacent keratocytes both *in vitro* as well as in *ex vivo* human corneas (Chen et al., 2020b). Species-dependent signaling responses occurred following TPMD with ATP and/or gap junctions acting as critical mediators. Thus, keratocyte TPMD is likely a common response to eye rubbing and a key mechanism by which these cells coordinate wound healing. Two recent meta-analyses of keratoconus studies identified eye rubbing as significantly associated with keratoconus with odds ratios of 5.22 and 6.46, respectively (Sahebjada et al., 2021; Seth et al., 2023). Balasubramanian and colleagues further demonstrated that eye rubbing for 1 min in normal individuals increased expression of IL-6, MMP13, and TNFα in tears (Balasubramanian et al., 2013). Thus, this local inflammation coupled with direct trauma to keratocytes and fluctuations in intraocular pressure incited by eye rubbing, particularly with greater force and frequency, likely plays a direct role in the pathogenesis of keratoconus. In summary, this data suggests that prospective studies of eye rubbing in keratoconic patients as well as investigations of TPMDs in this disease are warranted.

The mechanosensing role of focal adhesions was demonstrated by Maruri and colleagues whereby inhibition of focal adhesion kinase disrupted stiffness-dependent differences in keratocyte morphology and contractility as well as subcellular focal adhesion patterning (Maruri et al., 2022). In aggregate, this data suggests that downstream signaling of focal adhesions is critical during stiffness-dependent differentiation of corneal keratocytes. Indeed, the mechanotransducers, YAP and TAZ, form complexes with Smad 2/3 to regulate the TGFβ pathway (Fig. 3) and act as distinct modulators of KFM transformation (Morgan et al., 2013; Muppala et al., 2019). We demonstrated that simultaneous YAP and TAZ knockdown resulted in death of corneal stromal fibroblasts (Muppala et al., 2019). Furthermore, TAZ limits YAP’s ability to mediate KFM transformation via Smad proteins and loss of TAZ potentiates myofibroblast transformation even in the absence of TGFβ1. These data suggest that the YAP-TAZ signaling pathway can be exploited for therapeutic purposes. Indeed, celastrol, a pentacylic triterpenoid, inhibited corneal stromal fibrosis in a rabbit Descemet’s stripping endothelial keratoplasty (DSEK) model by modulating YAP/TAZ signaling and its downstream targets, Smad 2–3 and TGFβ (Liu et al., 2023). However, this approach needs careful context dependent evaluation. We previously showed that Hsp90 inhibition with 17AAG promoted reversion of the myofibroblast to keratocyte phenotype (Fig. 13), in the presence or absence of TGFβ1, on rigid substrates *in vitro* (Raghunathan et al., 2021). However, rabbits treated with 17AAG developed greater stromal haze formation and αSMA positive myofibroblasts compared with controls, irrespective of frequency of administration. Since Hsp90 inhibition affects both YAP/TAZ and the stability of TGFβ pathways, how mechanotransducers are targeted as therapeutics requires careful consideration.

**Fig. 17.**
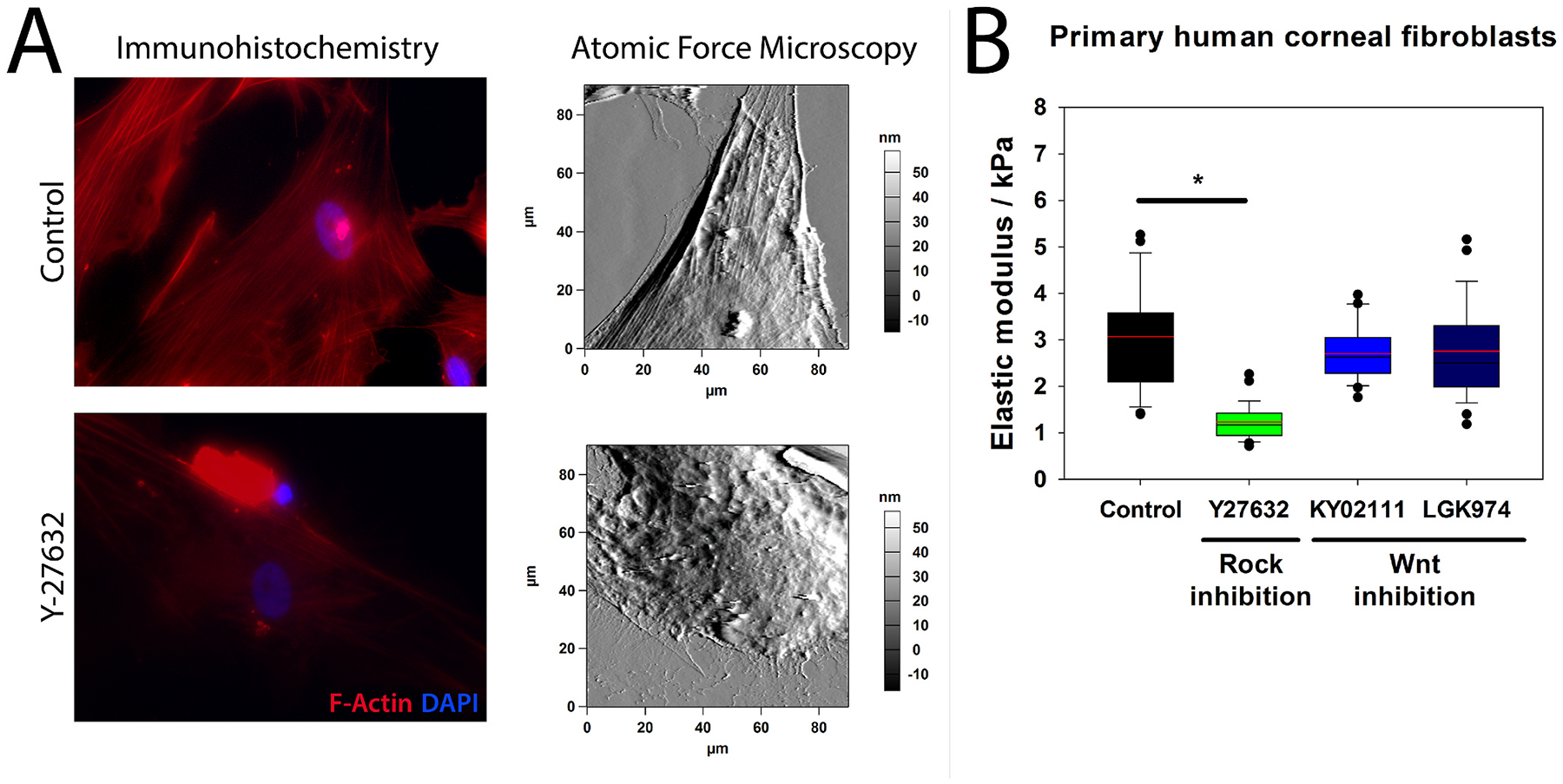
Differential responses of primary human corneal stromal fibroblasts to exogenous drug treatments. Primary human corneal stromal fibroblasts were cultured either untreated or with 10 μM ROCK inhibitor (Y27632), or 10 μM Wnt inhibitors (KY20111 or LGK974) for 3 days in growth media. **(A)** Cytoskeletal structure imaged by AFM demonstrates presence of stress fibers in control cells, but this was inconspicuously absent in Y-27632 treated cells. Fluorescent labelling with phalloidin (F-actin) and DAPI (nucleus) corroborates these findings. **(B)** Elastic moduli of cells were measured at the tallest region (over the nucleus) by AFM. While ROCK inhibition softened the cells, Wnt inhibition had no significant effects on moduli compared with untreated control cells. *p < 0.05, ANOVA followed by multiple comparison test against control.

**Fig. 18.**
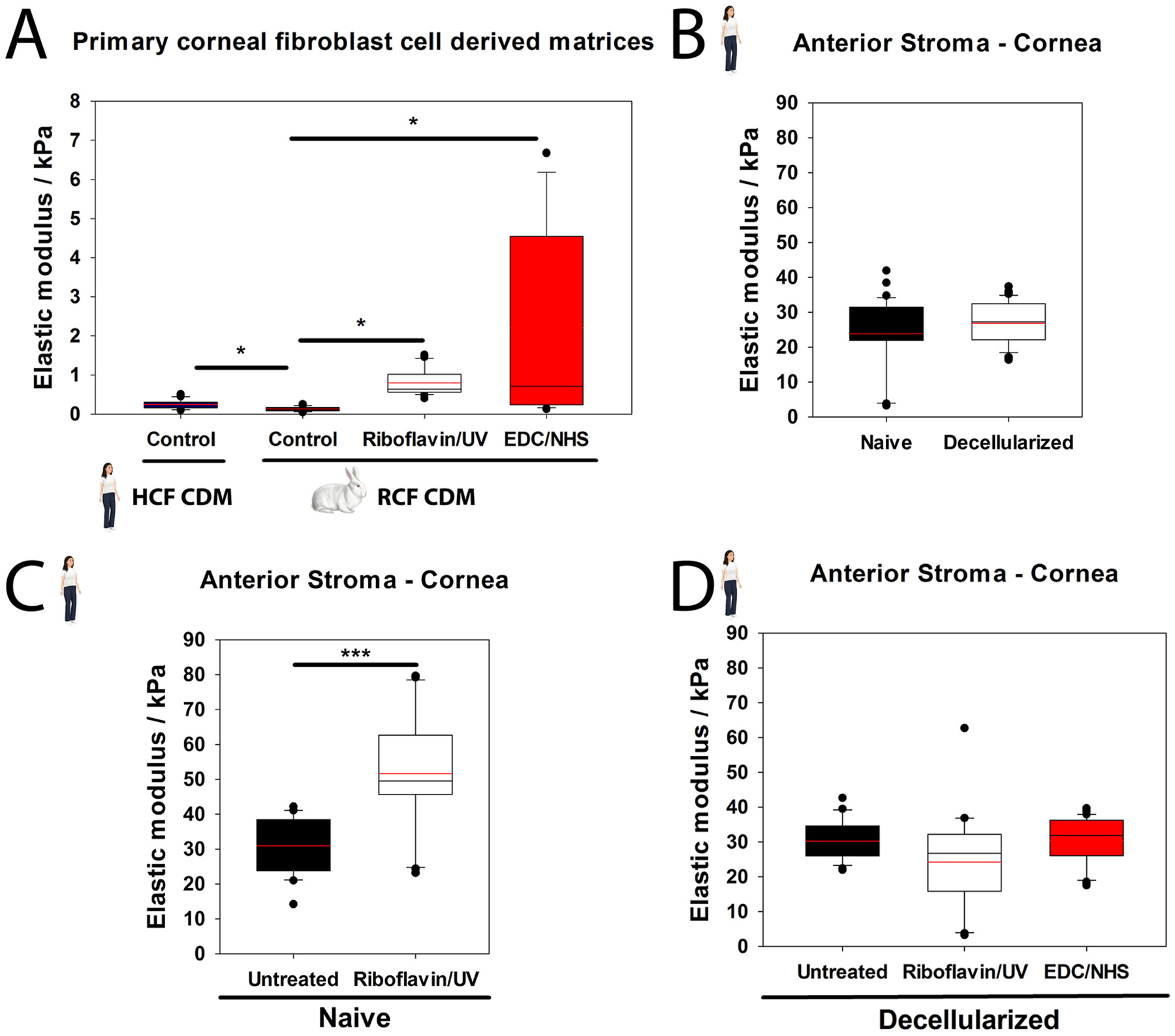
**(A) Modulation of mechanical properties through crosslinking of cell-derived extracellular matrices**. Primary human and/or rabbit corneal stromal fibroblasts (HCFs or RCFs, respectively) were cultured for 4 weeks in DMEM media supplemented with 10% FBS, penn/strep and 50 mg/ml ascorbic acid, with media changes performed twice weekly. At the end of the 4 weeks, cells were removed by successive incubation with a buffer containing 20 mM NH_4_OH, 0.05% Triton X-100 in water. Decellularization was confirmed by detecting the presence/absence of fluorescent labelling for F-actin and nucleus *(data not shown)*. Elastic moduli of decellularized cell-derived matrices were then either measured with no subsequent treatment or after crosslinking with either 0.05% riboflavin/UV-A for 15 min, or with EDC/NHS for 90 min. Both Riboflavin/UV-A and EDC/NHS crosslinking treatments potently increased matrix stiffness. **(B**–**D) Impact of decellularization on potency of crosslinking in human cornea. (B)** Elastic moduli of human cornea prior and after decellularization was comparable with no significant differences noted. Decellularization was achieved by using an antigen removal method without the use of sodium dodecyl sulfate (SDS). Decellularization was confirmed by H&E staining (*data not shown*) **(C)** Elastic moduli of the anterior stroma of naïve human corneas with cells intact were measured with no treatment or after treatment with Riboflavin/UV-A following epi-Off protocol. Significant stiffening of the stroma was observed as expected. ***P < 0.001, Mann-Whitney *U* test. **(D)** Human corneas were decellularized using the antigen retrieval method referenced above. Elastic moduli of decellularized corneas were measured with either no subsequent treatment or after crosslinking with riboflavin/UV-A or EDC/NHS. No significant differences in elastic moduli were observed between the groups by ANOVA.

It is important to note that the YAP/TAZ pathway orchestrates with multiple signaling pathways such as the TGFβ and Wnt pathways to partake in mechanotransduction events (Fig. 3). In corneal and non-corneal cells (such as adjacent trabecular meshwork cells), we demonstrated that treatment of primary stromal keratocytes or fibroblasts with TGFβ1 results in KFM transformation accompanied by cell stiffening and that this was at least partially dependent on substratum stiffness (Raghunathan et al., 2017). Further, Wnt inhibition in primary human trabecular meshwork cells resulted in substantial changes in cytoskeletal morphology accompanied by cell stiffening (Morgan et al., 2015), which was reversed when Wnt was activated (Dhamodaran et al., 2020). However, such stiffening after Wnt inhibition is not apparent in primary human corneal fibroblasts (Fig. 17). This suggests that modulation of different molecular signaling pathways may elicit different biomechanical responses in a cell-type dependent manner within the same organ i.e., the eye. Nevertheless, it is often thought that KFM transformation and mechanotransduction outcomes often result in substantial changes in the ECM. *In vivo*, discriminating the specific continuation of cells *vs* the ECM to biomechanical properties is challenging. As such, cell-derived matrices represent an excellent model to separately study the role of ECM in mechanics and to study the biology of cell-matrix interactions (Guo et al., 2007; McKay et al., 2019; Yemanyi et al., 2020). Herein, we document that the elastic moduli of ECM deposited by ascorbate-treated primary rabbit stromal fibroblasts is softer than those secreted by ascorbate-treated primary human stromal fibroblasts (Fig. 18) consistent with what is observed in stroma *in vivo* (Thomasy et al., 2014). We also show that the mechanical properties of these matrices may be altered by ultraviolet (UV) or chemical CXL methods to generate tunable biologically relevant matrices (Fig. 18) that have potential utility for investigating bidirectional cell-matrix interactions focused on mechanotransduction.

In the corneal stroma, YAP/TAZ also regulate development and lymphangiogenesis. Both YAP and TAZ are key drivers in cranial neural crest diversification and development by regulating Fox transcription factors, particularly *Foxc1* (Wang et al., 2016). YAP is particularly critical for corneal stromal development as YAP deficient (*Yap1*^+/−^) mice exhibited thin corneas, extensive fibrosis, abnormally arranged collagen fibrils, and increased keratocyte density in comparison to wildtype controls (Kim et al., 2019). In addition, an eye-specific isoform of inositol 1,4,5-triphosphate receptor 1 (ITPR1) regulates actin fiber directionality and focal adhesion expression through nuclear entry of YAP in neural-crest derived anterior ocular tissues (Kinoshita et al., 2021). Mice with a 7 base pair deletion in the last *ITPR1* exon demonstrate corneal stromal disruption with shredding actin fibers (Kinoshita et al., 2021). YAP/TAZ also plays a key role in balancing corneal prolymphangiogenic and antilymphangiogenic activities with lymphatic YAP/TAZ depletion increasing pathological lymphangiogenesis and YAP/TAZ overexpression markedly attenuating it (Cho et al., 2019).

### 4.9. Keratoconus – a disease with pathological biomechanics

Keratoconus is an exemplary stromal disease with pathological ECM, biomechanics and altered mechanobiology. It is a bilateral, progressive condition that results in vision impairment from abnormal thinning and protrusion of the cornea due to biomechanical instability within the stroma. Transcriptomic analyses of cultured corneal stromal cells from keratoconus patients demonstrated enrichment of genes involved in ECM, adherens junction, cell migration and MAPK signaling (Sharif et al., 2019). Within the corneas of keratoconus patients, RNAseq studies demonstrate disruption of collagen synthesis and maturation as well as downregulation of the mechanotransduction pathway, Hippo, and downstream signaling cascades including Wnt and TGFβ (Kabza et al., 2017; You et al., 2018). Epithelial-stromal interactions are critical in this disease with histopathological and *in vivo* confocal microscopy studies demonstrating changes in basal epithelial density and abnormalities in superficial epithelial cell differentiation early in the disease process (Efron and Hollingsworth, 2008). In the epithelium, β-catenin acts as a mechanotransducer that responds to changes in substrate stiffness delocalizing from the membrane to the cytoplasm resulting in cytoskeletal disruption, loss of barrier function as well as polarity in keratoconus (Amit et al., 2020). Interestingly, decreased and increased YAP expression has been reported in basal and superficial epithelial cells of keratoconic patients suggesting that further study is needed (Amit et al., 2020; Dou et al., 2022). In stromal cells, *YAP1* and *TEAD1* were upregulated in keratoconic patients as were their downstream proteases *MMP1, MMP3, CTSD* and *CTSK* implicating the role of mechanical stretch in disease progression (Dou et al., 2022). In aggregate, these studies suggest that modulating mechanotransduction, inhibiting ECM degradation and accelerating ECM production are pathways of interest for therapeutic intervention for keratoconus. For example, McKay and colleagues recently discovered that corneal fibroblasts from keratoconus patients and cultured in a 3D model had lower cytoplasmic arginine and spermidine concentrations in comparison to healthy controls (McKay et al., 2021). Furthermore, arginine supplementation resulted in markedly increased cytoplasmic arginine and its related metabolites as well as collagen type I production in the keratoconus-derived constructs suggesting a novel mechanism to treat this disease by increasing ECM deposition.

A key unmet need in the study of keratoconus is a biologically relevant animal model with thoroughly characterized stromal biomechanics. Wang and co-authors characterized a conditional TGFβ2 receptor deletion in the corneal stroma of mice as a putative model for corneal ectasia (Wang et al., 2023). These mice demonstrated stromal thinning, reduced collagen l1a1 expression, decreased collagen fibril density and corneal hydrops and edema following mechanical eye-rubbing consistent with what is observed in keratoconus. However, measurement of stromal elastic modulus was not performed. Enzymes that digest collagens such as amylase and collagenase contribute to the pathology of keratoconus and keratomalacia and it is thus important to quantify the biomechanical changes resulting from them (Collier et al., 2000; Conway et al., 2016; Donovan et al., 2021; Kazaili et al., 2021; Kimmitt et al., 2018; Mackiewicz et al., 2006). Unsurprisingly, elastic modulus and collagen fibril diameter as measured with AFM reduced in a dose-dependent manner when *ex vivo* porcine corneas were incubated with amylase or collagenase; cryosectioned tissue was used for AFM measurement thus impacting the absolute elastic modulus values (Kazaili et al., 2021). Intrastromal injection of collagenase II in rabbit corneas results in degradation and disorganization of stromal collagen, increased keratocyte density and a mild inflammatory infiltrate (Cano-Gómez et al., 2023). However, greater expression of type I collagen fibers with increased thickness was observed suggesting that normal rabbits are capable of rapid collagen regeneration and thus may not appropriately mimic the pathology of keratoconus (Cano-Gómez et al., 2023).

### 4.10. Therapeutics that may alter stromal biomechanics

The most evident therapeutic measure to fundamentally alter corneal biomechanics is in the form of collagen CXL that is performed to arrest or slow the progression of ectatic conditions, particularly keratoconus. In the last decade, numerous studies have documented the effects of (i) irradiance strength, duration, and intensity (Hammer et al., 2014; Kling and Hafezi, 2017), (ii) choice of epithelial debridement (Hafezi, 2022; Stojanovic et al., 2014; Wollensak and Iomdina, 2009), (iii) presence of basement membrane prior to the procedure, (iv) severity of the disease (Vinciguerra et al., 2017), (v) utility of riboflavin (Schumacher et al., 2011), (vi) age (Alenezi et al., 2022), and (vii) oxygen enrichment (Wang et al., 2020) on the efficiency of CXL and subsequent *in vivo* characterization. For a comprehensive review of biomechanics after corneal CXL, we direct the audience to the article by Blackburn and colleagues (Blackburn et al., 2021). Though UV-riboflavin CXL is widely performed, the mechanisms by which this happens is often debated. The most strongly supported hypothesis is that CXL occurs via a free-radical mediated reaction when riboflavin is exposed to UV light (Santhiago and Randleman, 2021). The source of the free radical is particularly unclear although it may be from the cells, and/or proteins in the extracellular milieu. Thus, to ascertain if cells are required for functional CXL, we determined stromal modulus prior to and after decellularization by ammonium hydroxide. Our data first demonstrates that decellularization alone is insufficient to alter the elastic modulus. However, when treated with UV-riboflavin CXL following a standard protocol, we noted that the process did not have any significant impact in decellularized corneas thus demonstrating the importance of the presence of stromal cells for effective CXL (Fig. 18).

**Fig. 19.**
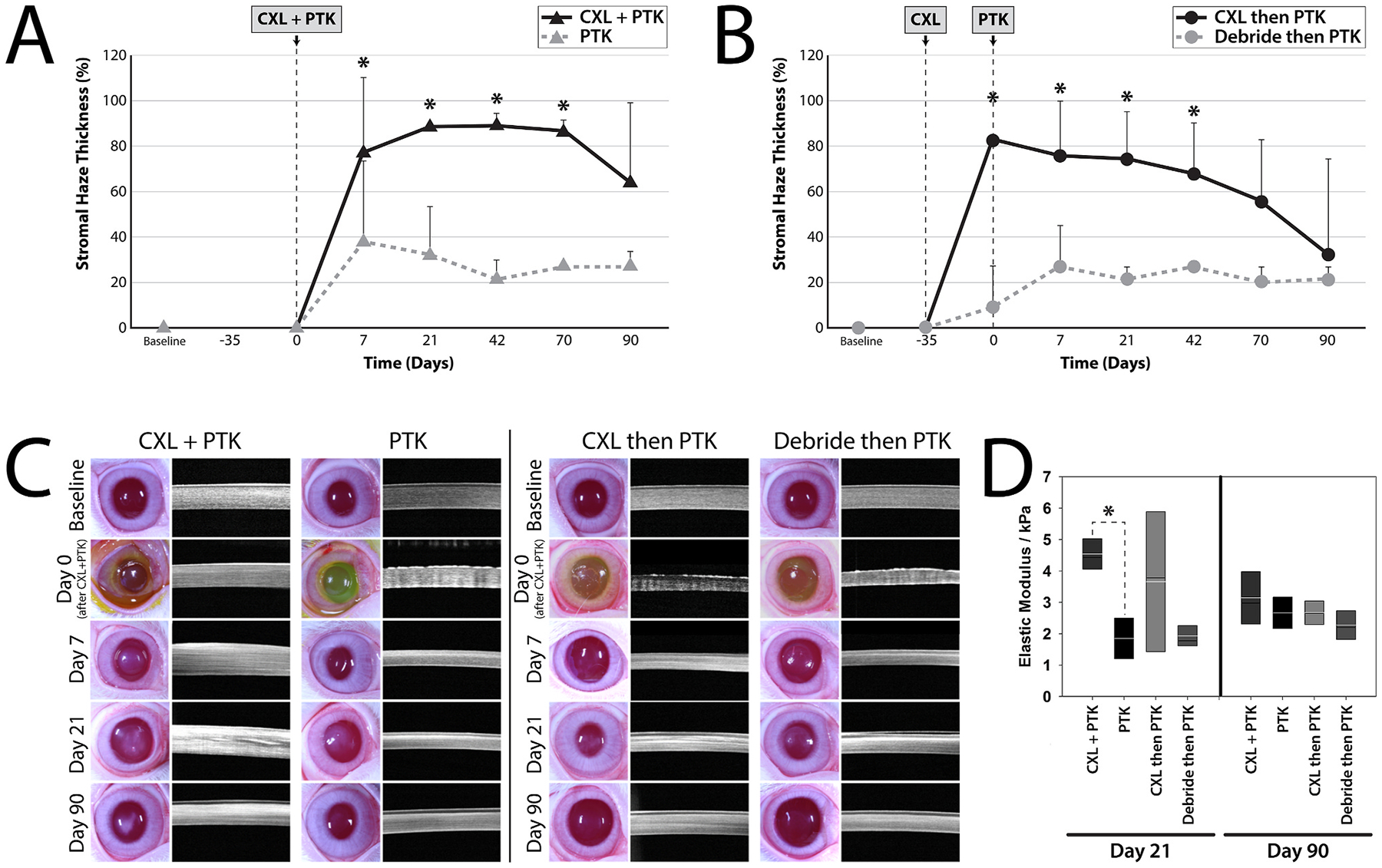
Collagen crosslinking (CXL) prior to or at the same time as corneal wounding with a phototherapeutic keratectomy (PTK) increased stromal haze relative to PTK alone in a rabbit model. **(A)** Stromal haze thickness (%) of the central cornea was significantly higher in the CXL groups than controls; CXL + PTK and CXL then PTK groups did not significantly differ **(B)** Representative color photographs and OCT images of a single rabbit in each group at baseline and days 0, 7, 21, and 90. Marked stromal haze was observed from day 7 until day 90 in the simultaneous CXL + PTK group. **(C)** The elastic modulus of corneas with simultaneous CXL and PTK was significantly higher than CXL then PTK corneas at day 21, but no significant differences were observed by day 90. In addition, no significant differences were observed between the CXL then PTK and the debride then PTK groups. *P < 0.05, repeated measure two-way ANOVA followed by Tukey’s multiple comparisons test. Reproduced from Moore, B.A., Jalilian, I., Kim, S., Mizutani, M., Mukai, M., Chang, C., Entringer, A.M., Dhamodaran, K., Raghunathan, V.K., Teixeira, L.B., Murphy, C.J., Thomasy, S.M., 2023. Collagen crosslinking impacts stromal wound healing and haze formation in a rabbit phototherapeutic keratectomy model Mol Vis 29, 101–115.

Keratorefractive surgery is increasingly being combined with UV-riboflavin CXL to prevent myopic or hyperopic regression following refractive surgery and decrease the development of corneal ectasia post-operatively (Evangelista and Hatch, 2018; Tomita, 2016; Wacker et al., 2015; Wollensak, 2006; Wollensak et al., 2003a, 2003b). This surgical combination could have increased risk of stromal haze formation particularly given our observation that increased stromal stiffness precedes KFM transformation in a rabbit PTK model (Raghunathan et al., 2017). Importantly, we demonstrated that simultaneous UV-riboflavin CXL and PTK increased corneal thickness, stromal stiffness, and fibrosis (Fig. 19). Taken together, these data suggest that CXL constrains interlamellar corneal stromal cell movement thus disrupting normal fibroblast entry, ECM deposition and remodeling. Indeed, a recent study in keratoconic patients found that PTK versus mechanical epithelial debridement had significantly more stromal haze formation (Cagil et al., 2015). We further demonstrated that pre-treatment with CXL then PTK 35 days later increased corneal fibrosis versus epithelial debridement then PTK suggesting that altered stromal biomechanics can have a persistent impact on corneal wound healing (Fig. 19).

Hyaluronidase, an enzyme that digests GAGs, has been purported to “soften” the stroma for procedures such as deep anterior lamellar keratoplasty (DALK) in order to facilitate separation of the stroma from Descemet’s membrane (Bonci et al., 2010; Connon et al., 2000; Lange et al., 2013). Contrary to this hypothesis, we demonstrated that intrastromal hyaluronidase in normal rabbit corneas transiently increased stromal stiffness with AFM as GAG degradation decreased interfibrillar spacing (Kim et al., 2020). Consistent with this study, keratan sulfate enzymatic digestion, which increases interfibrillar collagen spacing, softened corneal tensile properties in a porcine *ex vivo* model and could be reversed with CXL pre-treatment (Hatami-Marbini and Emu, 2023; Ho et al., 2014). These data and others suggest that interfibrillar collagen spacing is an important determinant of stromal stiffness (Kingsbury et al., 2023), and GAG composition can be therapeutically modulated to alter it. Further work is necessary to determine how these treatments would impact pathologic corneas undergoing DALK, particularly with fibrosis. Finally, hyaluronidase is used in ophthalmic practice to increase the onset of action of periocular local anesthesia (Rüschen et al., 2018) and facilitate sodium hyaluronate breakdown during cataract surgery (Calder and Smith, 1986), suggesting that the biomechanical implications in these tissues also need to be considered.

**Fig. 20.**
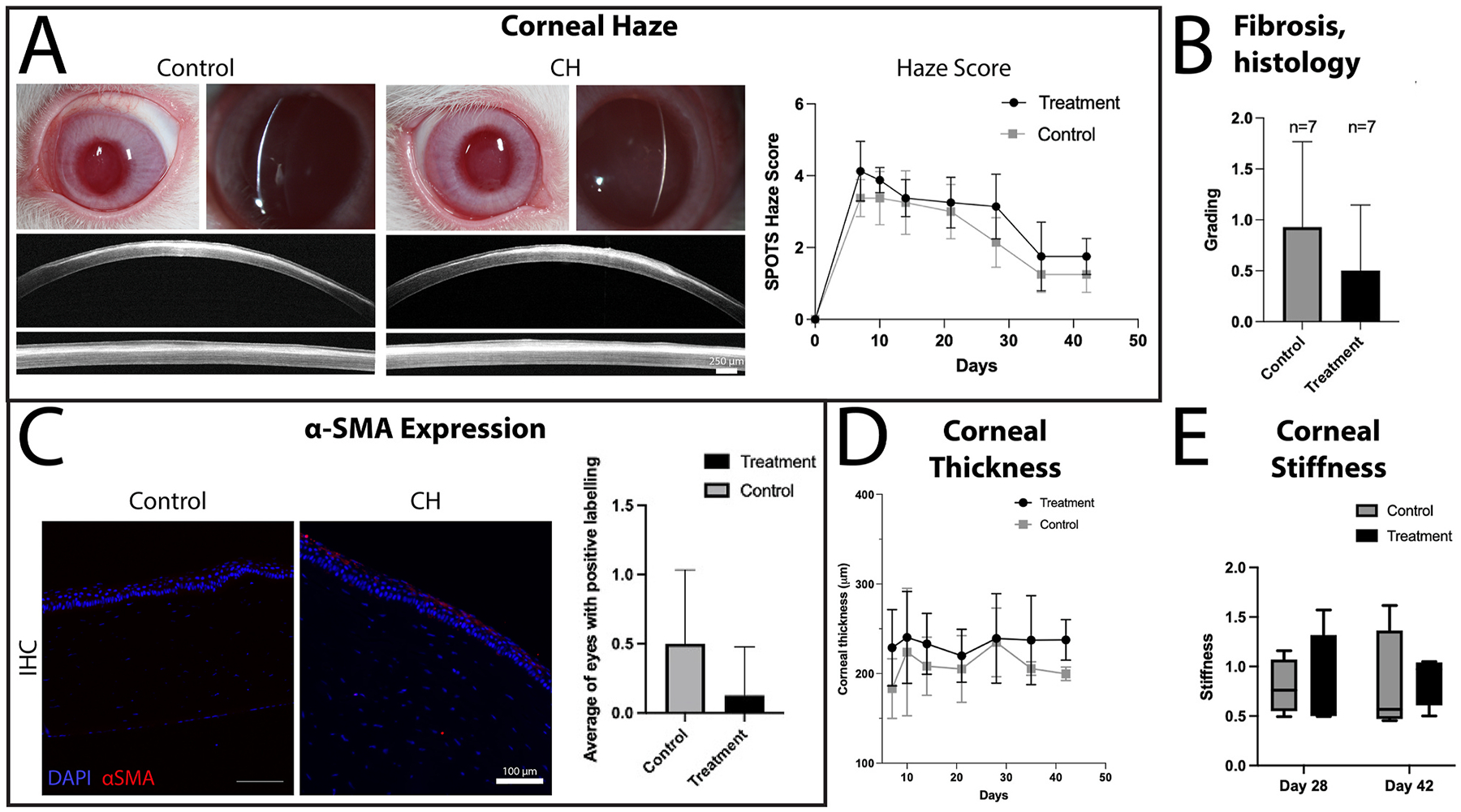
Administration of cysteamine hydrochloride, a TGM2 inhibitor, does not affect corneal scarring or stromal stiffness. Representative clinical, slit beam, and OCT images illustrating the area of corneal haze at final day 42 and graphic representation of clinical haze scores **(A)**. Corneal haze was present but not significantly different between control and CH treated eyes (n = 8 eyes per group to day 28, n = 4 eyes per group to day 42, P > 0.05 for all timepoints). Corneal fibrosis, as scored based on H&E histology sections, was not significantly different between treated and control eyes (n = 7 per group, P = 0.3473, **B**). Immunohistochemical α-SMA expression showed no differences between treated and control eyes, suggesting no appreciable change in KFM transformation (n = 7 per group, *P* = 0.2821, **C**). Corneal thickness as measured by OCT, was not different between control and treated eyes suggesting no difference in amount of scarring (n = 8 eyes per group to day 28, n = 4 eyes per group to day 42, *P* > 0.05 for all timepoints, **D**). Atomic force microscopy of sections from the area of wounded cornea showed no difference in stiffness between control and treated eyes (n = 4 eyes per time point, *P* = 0.99 at Day 28, *P* = 0.96 at Day 42. **E**). Reprinted from Minella, A.L., Casanova, M.I., Chokshi, T.J., Kang, J., Cosert, K., Gragg, M.M., Bowman, M.A., McCorkell, M.E., Daley, N.L., Leonard, B.C., Murphy, C.J., Raghunathan, V.K., Thomasy, S.M., 2023. The TGM2 inhibitor cysteamine hydrochloride does not impact corneal epithelial and stromal wound healing *in vitro* and *in vivo*. Exp Eye Res 226, 109338.

Tissue transglutaminase 2 (TGM2) is an important endogenous crosslinker in numerous tissues including the cornea such that its inhibition could adversely impact corneal wound healing. We tested the safety of cysteamine hydrochloride, a TGM2 inhibitor that is commercially available as Cystaran^®^, to treat the ocular symptoms of cystinosis (Minella et al., 2023). At the FDA-approved dose of cysteamine hydrochloride, corneal epithelial and stromal wound healing and stromal biomechanics were not impacted in a rabbit phototherapeutic keratectomy model suggesting that this medication could be safely used in cystinosis patients with corneal wounds (Fig. 20). Investigations of the efficacy of chemical crosslinkers, including genipin, are rapidly expanding for the treatment of corneal ectasia (Blackburn et al., 2021). Genipin increased stromal resistance to enzymatic digestion with collagenase (Donovan et al., 2021) as well as stiffened the normal rabbit cornea both alone and in combination with UV CXL (Tang et al., 2019). These studies serve as exemplars of strategies to modulate corneal CXL and underscore the importance of determining their impacts on stromal wound healing and biomechanics in relevant animal models.

## 5. Corneal endothelium

### 5.1. Anatomy and physiology of the corneal endothelium

The corneal endothelium is a functional unit lining the inner cornea that is composed of a cell monolayer and its specialized basement membrane, termed Descemet’s membrane (DM). In humans, corneal endothelial cells are terminally differentiated and functionally amitotic, arrested in the G1 phase for the entirety of postnatal life. Derived from neural crest tissue, peak corneal endothelial cell density occurs prior to birth, at ~5000 cells/mm^2^ (Mishima, 1982). Corneal endothelial cell density (ECD) then declines at a rate of 0.6% cells/mm^2^ per year (Armitage et al., 2003; Gambato et al., 2015).

The chief function of the corneal endothelium is to maintain proper corneal hydration through the active pumping of ions to counteract the passive leakage of fluid into the stroma. Specifically, the “pump-leak hypothesis” describes how Na^+^-K^+^-ATPase active transport actively pump ions and passively drives fluid into the anterior chamber to counterbalance the passive leakage of aqueous humor into the stroma through intercellular junctions (Maurice, 1972). Overall, monolayer integrity is required to control and counteract imbibition of aqueous fluid into the stroma. The barrier function of the corneal endothelium is maintained by tight junctions formed by claudins and occludens, including the key membrane-bound protein zonula occludens (ZO-1), as well as adherens junctions formed by cadherins and catenins. The pump function of the corneal endothelium is driven by Na^+^-K^+^-ATPase pumps that control hydration by establishing and maintaining an ion gradient; the number of Na^+^-K^+^-ATPase pump sites remains relatively constant despite declining ECD (Geroski and Edelhauser, 1984; Geroski et al., 1985). Other ions relevant to fluid transport and maintenance of appropriate corneal hydration have been proposed and include lactate and bicarbonate (Hodson and Miller, 1976; Hull et al., 1977; Li et al., 2020b, 2020c; Nguyen and Bonanno, 2011). For an extensive summary of the pump and barrier functions of the corneal endothelium, readers are directed to Klyce’s review (Klyce, 2020). Of note, an ECD lower than ~500 cells/mm^2^ results in the accumulation of fluid in the corneal stroma and loss of transparency due to swelling.

The corneal endothelium secretes DM, which consists of a fetal anterior banded layer that is formed in utero (total thickness of 3 μM at birth) and a posterior non-banded layer that grows in thickness throughout postnatal life (Lesueur et al., 1994; Murphy et al., 1984). The total thickness of healthy DM is 10–12 μM in adulthood (de Oliveira and Wilson, 2020; Johnson et al., 1982). The ECM of DM is comprised of collagen IV, collagen VIII, laminin, fibronectin, heparan sulfate and nidogens (de Oliveira and Wilson, 2020; Medeiros et al., 2018; Saikia et al., 2018). Collagen VIII forms the primary scaffolding structure in DM and connects to the posterior stroma via an interwoven collagen fibril network, with an interface between these two layers termed the interfacial matrix (Kabosova et al., 2007; Kapoor et al., 1988; Kingsbury et al., 2023; Schlotzer-Schrehardt et al., 2011).

### 5.2. Corneal endothelial repair varies by species

**Fig. 21.**
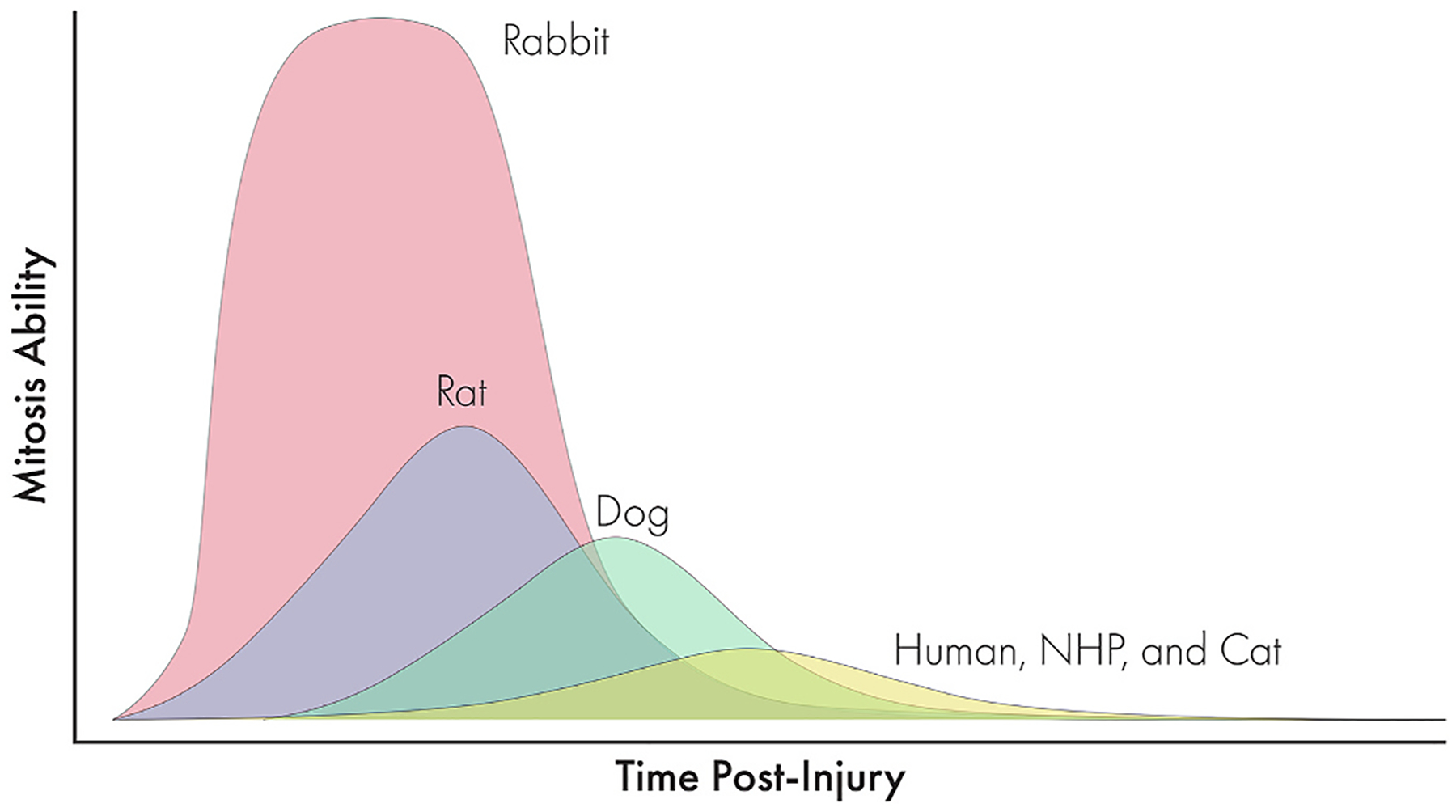
Variation in corneal endothelial proliferative capacity among species. Following corneal endothelial injury, rabbits demonstrate rapid and robust mitosis. The corneal endothelial cells of rodents also demonstrate some mitotic capacity although it is slower than in rabbits. Dogs, particularly young ones, also exhibit a proliferative corneal endothelium post-injury but it is slower than that of rodents. By contrast, cats, non-human primates (NHPs), and humans demonstrate little mitotic ability consistent with that of humans. Adapted from Park, S., Leonard, B.C., Raghunathan, V.K., Kim, S., Li, J.Y., Mannis, M.J., Murphy, C.J., Thomasy, S.M., 2021. Animal models of corneal endothelial dysfunction to facilitate development of novel therapies. Ann Transl Med 9, 1271, CC BY-NC-ND 4.0.

Because endothelial cell loss results in corneal edema, maintenance of this cell layer is critical to preserving vision. Corneal endothelial regenerative capacity varies dramatically by species with mitotic ability being greatest in rabbits, followed by rodents, then dogs, while cats, NHPs and humans all have limited mitotic ability (Fig. 21) (Park et al., 2021). In humans, corneal endothelial cells generally reside in the G1 phase of the cell cycle and typically spread rather than proliferate following injury to maintain cell-cell contact (Okumura et al., 2013b; Vedana et al., 2016). Human corneal endothelial cells have the capacity for self-renewal and retain the ability to proliferate but the factors inducing them to do so are poorly understood (McGowan et al., 2007; Yam et al., 2019; Zagórski, 1980). Indeed, precursor cells with the capacity to produce endothelial-like cells have been isolated from the endothelium of human donors but these cells are low in number and novel techniques are required to improve their recovery and delivery (Yokoo et al., 2005; Zhu and Joyce, 2004). As such, corneal transplantation with donor tissue has served as the primary means to restore endothelial health (Joyce, 2003; Terry, 2012). Vercammen and colleagues recently reviewed corneal endothelial wound healing for readers that want to delve more deeply into this topic (Vercammen et al., 2022).

### 5.3. The corneal endothelium and DM are intimately connected

As our review of the biomechanical relationships between the endothelium and DM attests (Ali et al., 2016), biomechanical properties (e.g., stiffness and topography) of both DM and the ECM of the posterior cornea profoundly impact endothelial cell morphology and function. There is growing evidence that this dynamic bidirectional signaling between corneal endothelial cells and DM governs a variety of vital functions including contractility, proliferation, migration, adhesion, polarity, and survival (Crowder et al., 2016; Gasiorowski et al., 2013; Gruschwitz et al., 2010; Humphrey et al., 2014; Iskratsch et al., 2014; Leonard et al., 2019b; Wang et al., 2012). Consequently, perturbations in the health and composition of DM can lead to maladaptive signaling and changes in gene expression and cell behavior that result in cell death and failure of the corneal endothelium to maintain corneal homeostasis and proper visual function. The key signaling pathways involved in mechanotransduction in the corneal endothelium are reviewed herein, with a focus on their role in emerging issues surrounding the mechanobiology of the corneal endothelium relating to Hippo, TGBβ, and Wnt pathway signaling. As in other cell types, molecular crosstalk between pathways is an important reason for multiple cellular responses observed in the dynamic reciprocity between DM and corneal endothelial cells (Piersma et al., 2015a).

As detailed in 2.3.2 YAP/TAZ and Hippo signaling, the cellular location of Hippo pathway mediators YAP and TAZ are important in proper functioning of cells, including the corneal endothelium, and their surrounding ECM environment. In constructing and testing scaffolds for tissue-engineered human corneal endothelial cell grafts, Zhao and colleagues utilized YAP translocation to the nucleus as an indicator of proper cell functionality (Zhao et al., 2020, 2022). They determined that corneal endothelial cell function improved with modification of matrix carrier mechanical properties (e.g., crosslinking, coating with laminin and fibronectin) including improved adhesion and proliferation (Zhao et al., 2022), and demonstrated that administration of laminin 511 and cultured primary endothelial cells resulted in a rapid decrease in corneal thickness in a rabbit mechanical injury model (Zhao et al., 2020). Of note, the role of YAP and TAZ in responding to altered DM stiffness in FECD is particularly relevant to understanding disease mechanisms and therapeutics in this common disease (Leonard et al., 2023), and is reviewed in 5.4. Fuchs’ endothelial corneal dystrophy, an exemplar of primary ECM pathology in greater detail.

In the corneal endothelium, activation of the TGFβ pathway results in loss of cell phenotype, redox imbalance and oxidative stress, increased production of ECM proteins, matrix remodeling and inhibition of cell growth, with the TGFβ1 and TGFβ3 isoforms regarded as stimulants of EnMT (Liu and Desai, 2015; Liu and Gaston Pravia, 2010; Okumura et al., 2013a, 2017). Prolonged TGFβ exposure leads to accumulation of ECM products and triggers unfolded protein response, endoplasmic reticular stress, and eventually apoptosis (Okumura et al., 2013a, 2017; Pozzer et al., 2017). Indeed, TGFβ plays a crucial role in corneal endothelial cell senescence and loss in chronic corneal graft failure (Li et al., 2018). Importantly, blockade of TGFβ1 preserves corneal endothelial cell hexagonal shape and downregulate EnMT markers (Wang et al., 2022b). By contrast, the TGFβ2 isoform strengthens cell-cell and cell-substrate adhesion, accelerate monolayer barrier integrity establishment, and thus enhance corneal endothelial functionality (Santerre et al., 2022). Continued investigations into the contextual effects of TGFβ isoforms will likely be of growing importance to efforts to develop engineered corneal endothelial cell grafts.

Similarly, the Wnt pathway has been implicated in regulating EnMT. In a targeted study demonstrating the ability of a human amniotic epithelial cell culture microenvironment to stimulate corneal endothelial cell proliferation, Liu and colleagues determined that such corneal endothelial cell proliferation is related to regulating telomerase activity and EnMT via the Wnt/β-catenin pathway (Liu et al., 2021). Leucine-rich repeat-containing G protein-coupled receptor 5 (LGR5), a marker of stem/progenitor cell populations in self-renewing tissues, is enriched in peripheral human corneal endothelial cells, supports proliferation and endothelial phenotype, and inhibits EnMT via Wnt and hedgehog pathway signaling (Hirata-Tominaga et al., 2013). Additionally, Roof plate-specific spondin 1 (R-spondin1), a LGR5 ligand, accelerates corneal endothelial cell proliferation and inhibits EnMT through the Wnt pathway (Okumura et al., 2014a) By contrast, siRNA knockdown of *COL8A2*, a gene that is mutated in early-onset FECD, resulted in Wnt pathway signaling disruption reduced proliferation and fibroblastic transformation indicating both the importance of COL8A2 for corneal endothelial cell function as well as an opportunity to target Wnt pathway mediators in this disease (Hwang et al., 2020). In aggregate, Wnt signaling represents an important modulatory pathway for corneal endothelial cell fate, proliferation and function that will be relevant in the development of alternative therapeutics to keratoplasty.

**Fig. 22.**
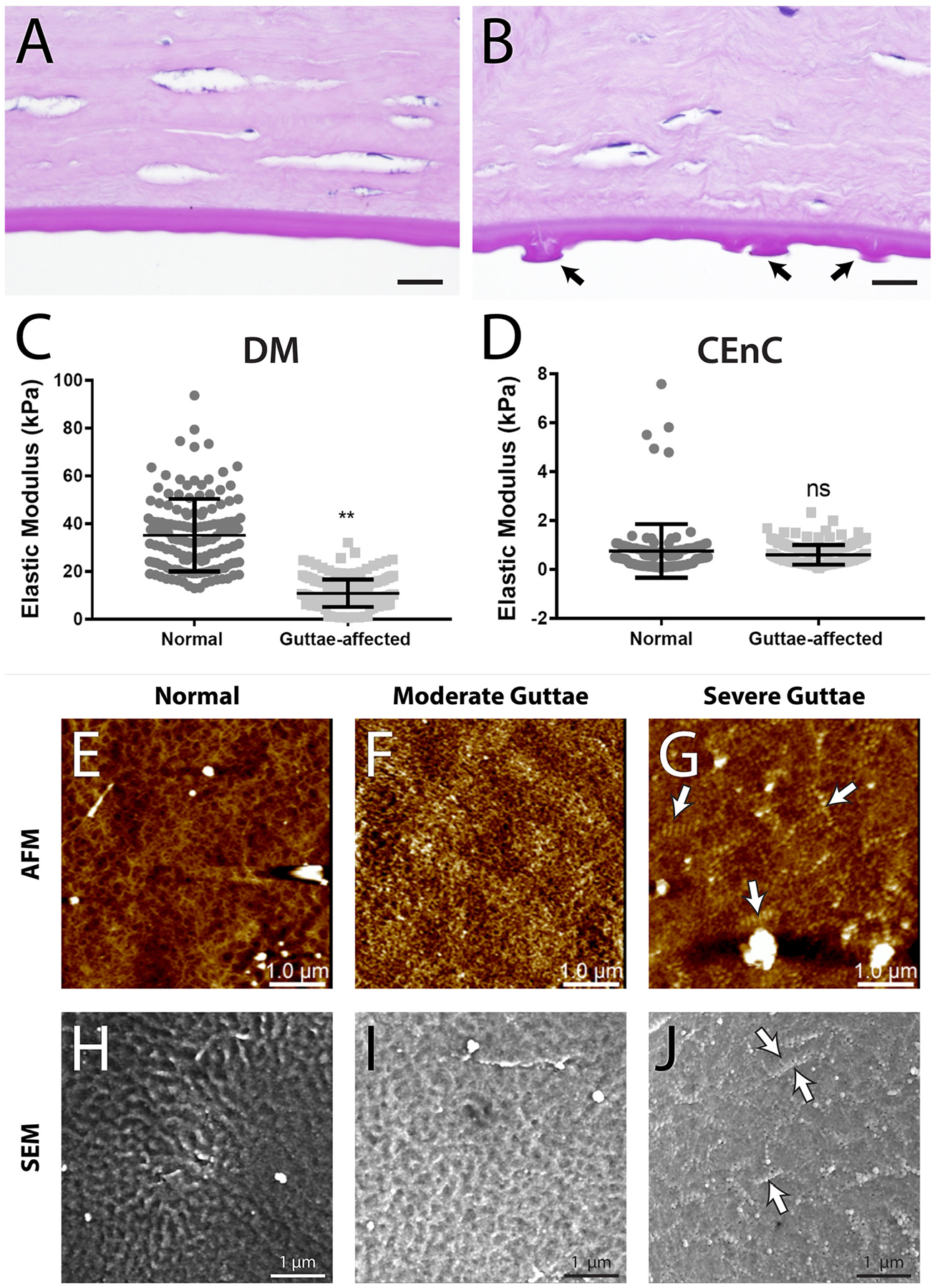
Stiffness and topography of corneal Descemet’s membranes (DM) differed between normal and guttae-affected donors. Elastic moduli of DM were significantly higher in normal versus guttae-affected donors whereas elastic moduli of the endothelium were similar. Representative histologic images of a decellularized healthy DM from a 65-year-old female **(A)** and a decellularized guttae-affected DM from a 69-year-old male **(B)** with three large guttae (excrescences of extracellular matrix on DM, arrows) observed. Scale bar equivalent to 20 μm. Elastic modulus of DM **(C)** and corneal endothelial cells **(D)** in healthy donors (n = 10) and guttae-affected donors (n = 6). Data points represents single atomic force microscopy (AFM) measurements from all individuals within the healthy or guttae-affected patients. Horizontal lines indicate mean and error bars represent SD. The P values were determined by a Mann-Whitney rank sum test (DM) and Student’s t-test (endothelium), **P < 0.01. ns: not significant. The DM of normal corneas and those with guttae were critical point dried and imaged by AFM **(E**–**G)**. Tissues were glued to AFM compatible dishes on their epithelial side and were then scanned at a rate of 0.1 μm/s under PeakForce Tapping^™^ mode using a AC-240TS cantilever. Each image was taken in 1024 × 1024 pixel and the height channel was used to show the details of the surface of normal and guttae-affected DMs. The DMs severely affected with guttae demonstrate collagen structures on their surface (**G, blue arrows**) which were not observed in controls **(E)** and corneas moderately affected with guttae **(F)**. Scale bar, 1 μm. Subsequent to imaging by AFM, samples were sputter coated with gold and imaged using a scanning electron microscope **(H**–**J)** which validated dysregulated collagen banding in samples severely affected with guttae **(J)** which was absent in control **(H)** and corneas moderately affected with guttae **(I)**. Figure and legend adapted from Leonard, B.C., Park, S., Kim, S., Young, L.J., Jalilian, I., Cosert, K., Zhang, X., Skeie, J.M., Shevalye, H., Echeverria, N., Rozo, V., Gong, X., Xing, C., Murphy, C.J., Greiner, M.A., Mootha, V.V., Raghunathan, V.K., Thomasy, S.M., 2023. Mice Deficient in TAZ (Wwtr1) Demonstrate Clinical Features of Late-Onset Fuchs’ Endothelial Corneal Dystrophy. Invest Ophthalmol Vis Sci 64, 22 with unpublished data.

Currently, corneal transplantation using tissue-targeted surgical techniques is required to treat corneal edema secondary to corneal endothelial dysfunction. Endothelial keratoplasty, which is the surgical resection of the corneal endothelium and DM and its replacement with donor corneal endothelium and DM, is the current standard of care treatment for most disorders of the corneal endothelium resulting in corneal edema. There are two main endothelial keratoplasty surgical techniques that differ based on the preparation and amount of donor corneal tissue: DM endothelial keratoplasty (DMEK), in which the donor tissue is comprised solely of DM and corneal endothelium (e.g., a 1:1 replacement), and Descemet’s stripping automated endothelial keratoplasty (DSAEK), in which the donor tissue is comprised of DM-endothelium and posterior stromal tissue (e.g., tissue is added). However, the limited availability of donor corneal tissue with only ~1 cornea available for 70 needed worldwide and risks of donor tissue failure (immediate risk) and graft rejection (lifetime risk) have spurred the development of alternative non-surgical treatments for endothelial disease (Gain et al., 2016). A promising solution is the expansion of corneal endothelial cells *in vitro* for delivery to patients. In culture conditions, corneal endothelial cells are grown frequently on substrates coated with ECM proteins found in DM including laminin and chondroitin sulfate (Engelmann et al., 1988), laminin-5 (Yamaguchi et al., 2011), bovine secreted ECM from corneal endothelial cells (Blake et al., 1997), or fibronectin plus type I collagen (Joyce and Zhu, 2004). These ECM coatings can be used to control corneal endothelial cell adhesion, proliferation, migration, and/or maintenance of endothelial phenotype. Biomechanical cues including substratum topography and compliance have been instrumental in promoting optimal endothelial cell phenotypes in culture. Surface micro- and nanotopographical cues in combination with various ECM coatings modulate proliferation, morphometry, phenotype and functionality of immortalized and primary cultured corneal endothelial cells (Koo et al., 2014; Muhammad et al., 2015). Consistent with studies demonstrating that DM is soft in patients with FECD (Fig. 22), (Leonard et al., 2023; Xia et al., 2016), Niu and colleagues found that primary cultured human corneal endothelial cells at higher densities achieved increased proliferation and typical cell morphology on stiffer versus softer scaffolds (Niu et al., 2014).

Cultured cells can then be seeded on a variety of substrates (Tsai and Daniels, 2021), ranging from decellularized native tissues (e.g., DM and corneal stroma) (An et al., 2020; Lu et al., 2020) to natural polymers (e. g., collagen I hydrogel and gelatin) (Levis et al., 2012; Niu et al., 2014) and synthetic materials (e.g., poly-ε-lysine hydrogel) (Kennedy et al., 2019), with each iteration having benefits and limitations that necessitate thorough experimental testing *in vivo*. Furthermore, corneal endothelial tissue grafts can be bioengineered to deliver donor corneal cells expanded in culture coupled with a donor cornea or biosynthetic basement membrane substrate that supports cell health and surgical handling. Crouzet and colleagues have described the successful surgical implantation of corneal endothelial cell grafts adhered to human decellularized femtosecond laser-cut ultrathin stromal lamellae in a rabbit model (Crouzet et al., 2022). Additionally, Zhao and colleagues have described the successful surgical implantation of tissue engineered corneal endothelial cell grafts adhered to crosslinked and ECM-coated decellularized amniotic membranes in both cats and nonhuman primates (Zhao et al., 2022). As noted above, investigators in that study utilized YAP translocation to the nucleus as an indicator of proper cell functionality. Indeed, with the noted importance of substrate stiffness on proliferation, phenotype maintenance and functionality, biomechanical testing of engineered corneal endothelial tissue grafts represents an opportunity for validating cell health on such grafts. Guiding principles in the establishment and maintenance of healthy bioengineered corneal endothelial tissue grafts will undoubtedly need to attend to the influence of mechanotransduction and ECM properties on cell health. Tsai and Daniels recently discussed the impact of biomechanics on corneal endothelial tissue engineering for readers who want to delve more deeply into this topic (Tsai and Daniels, 2021).

### 5.4. Fuchs’ endothelial corneal dystrophy, an exemplar of primary ECM pathology

In the United States, FECD affects ~6 million adults and is the most common indication for corneal transplantation (Afshari et al., 2017; Stamler et al., 2013). Corneal endothelial cell loss and guttae are defining features of this disease. Furthermore, FECD alters the composition of DM with increased deposition of collagen I, collagen VIII, fibronectin, and laminin contributing to an overall thickening of this basement membrane (Gottsch et al., 2005; Levy et al., 1996). It is thus unsurprising that missense mutations in collagen VIII α2 (*COL8A2*) are responsible for a severe, early onset form of FECD (Biswas et al., 2001). In this type of FECD, abnormal deposition of COL8A2 protein is present throughout DM with ultrastructural changes that include large amounts of disorganized collagen VIII and the excessive growth of the fetal anterior banded layer of DM (Gottsch et al., 2005). Biomechanical abnormalities in the early onset form of FECD including matrix stiffness alterations that critically impact cell health (Leonard et al., 2019b). By contrast, abnormal trinucleotide repeat (TNR) expansions of the transcription factor 4 (*TCF4*) gene accounts for >70% of FECD cases in older adults (Afshari et al., 2006). In this late onset form, mis-splicing of RNAs encoding for cytoskeletal, cell adhesion, and ECM organizational proteins occurs (Fautsch et al., 2021; Mootha et al., 2015; Wieben et al., 2017). In ungenotyped samples from patients with late onset FECD, Weller and colleagues found increased mRNA and protein expression of ECM components collagen types III and XVI, TGFβ-induced (TGFBI), agrin, and clusterin (Weller et al., 2014). While the clinical characteristics of FECD patients with *COL8A2* mutations or TNR expansions of *TCF4* have been described (Igo et al., 2012), the biomechanical properties and proteome of DM in FECD patients with genotype-confirmed TNR expansions of *TCF4* have not been characterized thus representing an important gap in our knowledge of this disease.

Nonhuman primates demonstrate similar changes in corneal thickness and endothelial density with age as in humans but FECD-like disease appears to be rare (Casanova et al., 2022). In dogs, an analogous condition to FECD exists, termed corneal endothelial dystrophy (CED). A retrospective review of canine CED identified 10 predisposed breeds and demonstrated that dogs >11 years of age were overrepresented (Leonard et al., 2021). Median time to initiation of edema in an at-risk eye and progression from focal to diffuse edema in CED-affected dogs was ~1 year. Further characterization of this condition in three at-risk breeds, Boston Terriers, German Shorthaired and Wirehaired Pointers, revealed reduced ECD, endothelial polymegathism and pleomorphism, progressive corneal edema with increased thickness, a thickened DM-endothelial complex and guttae-like lesions (Shull et al., 2018; Thomasy et al., 2016a). The model has been utilized to investigate a novel surgical procedure (Horikawa et al., 2016) and the ROCK inhibitor ripasudil (Michalak et al., 2022) over a 1-year period since CED progresses more quickly in canine patients than FECD does in humans. In aggregate, the striking similarities between FECD and CED combined with the similar environments that dogs and humans reside in suggest that successful therapies for CED may be more likely to translate successfully to FECD patients than those tested solely in healthy animal models with induced corneal endothelial injury.

The softening of DM is a key feature of FECD and its animal models and contrasts with corneal fibrosis whereby matrix stiffening occurs. We and others demonstrated that patients with guttae and FECD have a markedly softer DM (Fig. 22) (Leonard et al., 2023; Xia et al., 2016). Concomitant with this observation, ultrastructural changes to DM in FECD include wide-spaced and fibrillar collagen deposited on the posterior DM (Xia et al., 2016). As these ECM changes modulate endothelial cell behavior and function (Ali et al., 2016; Muhammad et al., 2015; Öztürk-Öncel et al., 2021), it is critical for animal models to recapitulate these changes. Importantly, we demonstrated that *Col8a2*^*Q455K/Q455K*^ and *Col8a2*^*L450W/L450W*^ mice, which recapitulate features of early-onset FECD including guttae and endothelial pleomorphism (Jun et al., 2012; Meng et al., 2013), had softer DMs that preceded endothelial cell loss (Leonard et al., 2023). Furthermore, this model demonstrates endoplasmic reticulum stress, unfolded protein response and altered autophagy critical in the pathogenesis of FECD (Martínez-Chacón et al., 2020; Meng et al., 2013). Mechanotransduction and autophagy are highly interconnected and regulate each other in a process known as mechanoautophagy (Ravasio et al., 2022). Thus, investigating mechanoautophagy in this model will provide key insights regarding how ECM changes modulate endothelial pathology in FECD.

**Fig. 23.**
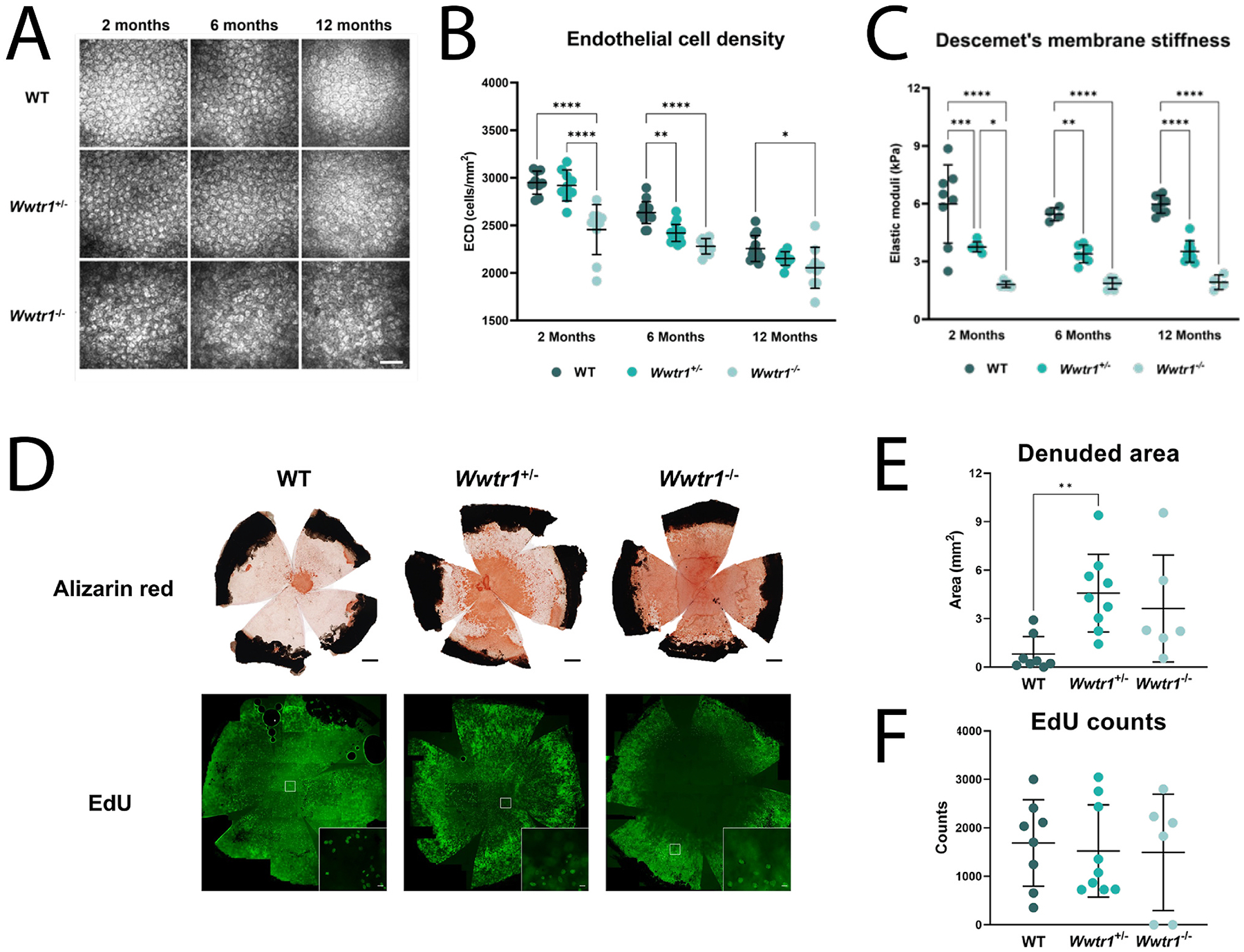
TAZ-deficient mice exhibit reduced corneal endothelial cell density (ECD), abnormal morphology, softer DM, and delayed endothelial wound healing when compared with WT controls. **(A**–**B)** *In vivo* confocal microscopy revealed reduced ECD in both *Wwtr1*^+*/*−^ and *Wwtr1*^−/−^mice at 2 months compared with WT mice. Despite age-related corneal endothelial cell loss, TAZ-deficient mice continued to have further declines in ECD at 6 and 12 months of age. Additionally, endothelial cells from TAZ-deficient mice demonstrated abnormal morphology (lower percentage of hexagonal cells, higher shape variability, altered reflectivity) when compared with WT mice. Scale bar equivalent to 50 μm. **(B)** Each dot represents a single eye (n ≥ 9 eyes in each group); horizonal line represents mean of the group and error bars reflect standard deviation. **(C)** Atomic force microscopy (AFM) performed on DM revealed decreased elastic modulus (softer) in TAZ-deficient mice, with *Wwtr1*^−*/*−^ mice having the lowest measurement, followed by *Wwtr1*^+*/*−^ mice and finally WT mice (stiffest). Each dot represents a single eye (n ≥ 9 eyes in each group), horizonal line represents mean of the group and error bars reflect standard deviation. A two-factor ANOVA was performed to detect statistical differences, *, **, ***, **** represent *P* < 0.05, *P* < 0.01, *P* < 0.001, *P* < 0.0001, respectively. *Wwtr1* deficient mice demonstrated impaired corneal endothelial cell regeneration after corneal cryoinjury **(D**–**F)**. A cryoinjury wound was created with a 2 mm diameter steel probe immersed in liquid nitrogen for 3 min (−196 °C) and subsequently applied to the cornea for 10 s in WT (n = 8), *Wwtr1*^+*/*−^ (n = 8), and *Wwrt1*^−*/*−^ (n = 5) mice. On day 2, animals were euthanized, and the right eye was stained with Alizarin red to calculate total denuded area **(D)** and the left eye was stained with EdU to assess cell proliferation **(E**–**F)**. On day 2, there was a significantly larger denuded area in *Wwtr1*^+*/*−^ mice compared with WT mice **(E)**. Additionally, *Wwtr1*^−*/*−^ mice had a trend towards larger denuded areas compared with WT mice **(E)**. There were no differences in EdU staining, indicating that the proliferative capacity of the corneal endothelial cells was equal across groups **(F)**. Both Alizarin red and EdU staining were analyzed using Kruskal-Wallis tests, **P < 0.01. Alizarin red scale bars equivalent to 500 μm and EdU staining scale bars equivalent to 20 μm (inset). Figure and legend adapted from Leonard, B.C., Park, S., Kim, S., Young, L.J., Jalilian, I., Cosert, K., Zhang, X., Skeie, J.M., Shevalye, H., Echeverria, N., Rozo, V., Gong, X., Xing, C., Murphy, C.J., Greiner, M.A., Mootha, V.V., Raghunathan, V.K., Thomasy, S.M., 2023. Mice Deficient in TAZ (Wwtr1) Demonstrate Clinical Features of Late-Onset Fuchs’ Endothelial Corneal Dystrophy. Invest Ophthalmol Vis Sci 64, 22.

There is increasing evidence indicating that ECM abnormalities and cell death may be linked mechanistically in FECD via the mechanotransducers, YAP and TAZ. Hsueh and co-authors documented that immortalized human corneal endothelial cells transfected with YAP or treated with lysophosphatidic acid to induce YAP nuclear translocation promoted cell proliferation while preventing EnMT (Hsueh et al., 2015). We have conducted studies using transgenic mice to further investigate the role of these Hippo pathway effector molecules on ocular morphology with an emphasis on corneal endothelial cell health (Kim et al., 2019; Leonard et al., 2019b). We generated mice deficient in YAP (*Yap1*^+*/*−^) since homozygous deletion is embryonically lethal and demonstrated severe changes in ocular morphology including a thin DM and absent corneal endothelium (Kim et al., 2019). Furthermore, mice with a deletion in *ITPR1*, which reduces YAP nuclear entry, have changes to all corneal layers including a thin, disrupted corneal endothelium (Kinoshita et al., 2021). By contrast, TAZ-deficient mice (*Wwtr1*^+*/*−^ and *Wwtr1*^−*/*−^) had normal appearing eyes but a soft DM with abnormal endothelial morphology and reduced ECD (Fig. 23) (Leonard et al., 2023). We further demonstrated that delayed endothelial repair occurred in TAZ-deficient mice following cryoinjury as well as phototherapeutic keratectomy (Fig. 23). In aggregate, these mice recapitulate a number of features of late-onset FECD. Thus, TAZ-deficient mice are an important disease model for studying how aberrant corneal endothelial mechanotransduction results in DM softening and endothelial cell loss as well as investigating novel therapeutic strategies for FECD.

**Fig. 24.**
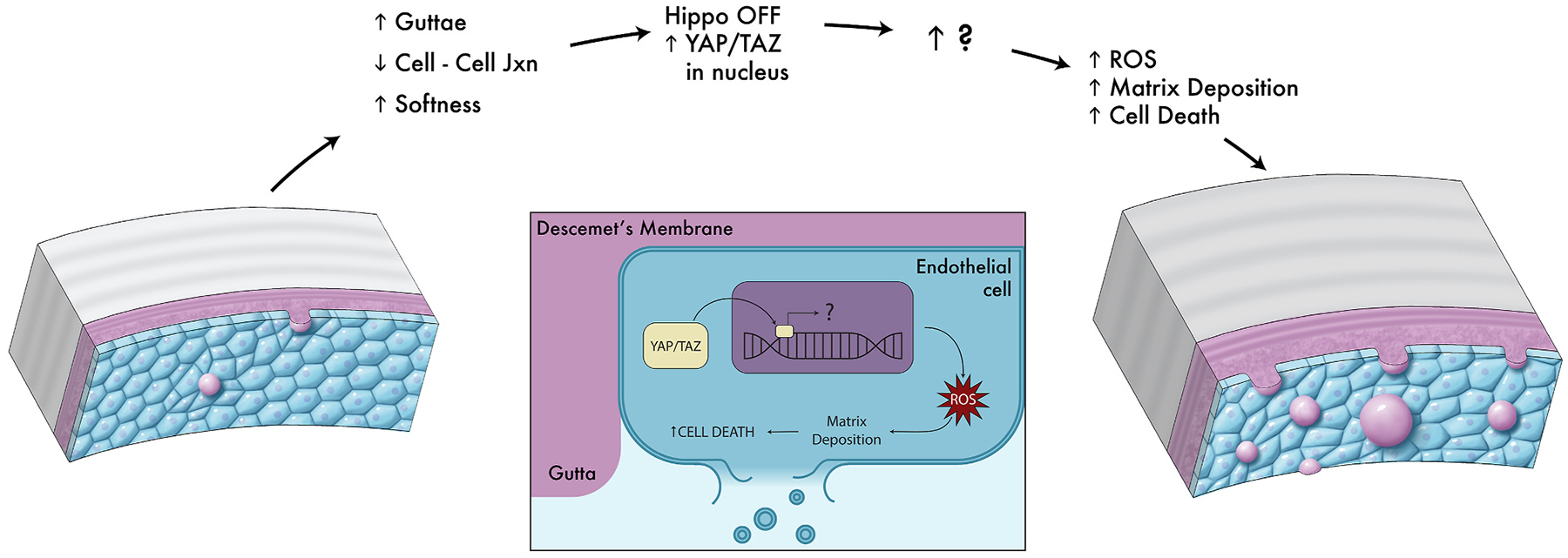
ECM pathology in DM impairs corneal endothelial cell function in FECD. We hypothesize that abnormal ECM deposition leads to YAP/TAZ upregulation and nuclear translocation (Hippo pathway inactivation) and thus contributes to corneal endothelial cell death. Cell death, in turn, drives further dysfunction and abnormal ECM deposition of surrounding corneal endothelial cells that leads to an escalating cycle of pathology in the posterior cornea in FECD.

The formation of guttae and the softening of DM define fundamental features of ECM mediated pathology that modulate key corneal endothelial behaviors in FECD (Fig. 24). Raphael and coauthors found that endothelial cells formed a confluent monolayer on normal and FECD-affected DMs but that the healthy polygonal cell pattern was disrupted by guttae with only cell processes covering them (Raphael et al., 1992). Kocaba and colleagues further demonstrated that small and medium sized guttae remained buried under an intact corneal endothelial cell monolayer, while large guttae disrupted monolayer confluence and induced apoptosis in corneal endothelial cells surrounding guttae *in vitro* (Kocaba et al., 2018). Importantly, upregulation of EnMT, senescence, and apoptosis gene expression were noted in cells surrounding large guttae suggesting that these were more pathological than smaller guttae. Consistent with this study, Rizwan et al. documented that increased guttae density and height prevented corneal endothelial cell monolayer reformation using a synthetic guttae *in vitro* model (Rizwan et al., 2016). In aggregate, these data suggest that guttae dimensions, density, and spacing all impact corneal endothelial cell migration and monolayer reformation and provide additional evidence that DM topography modulates corneal endothelial cell pathology in FECD.

**Fig. 25.**
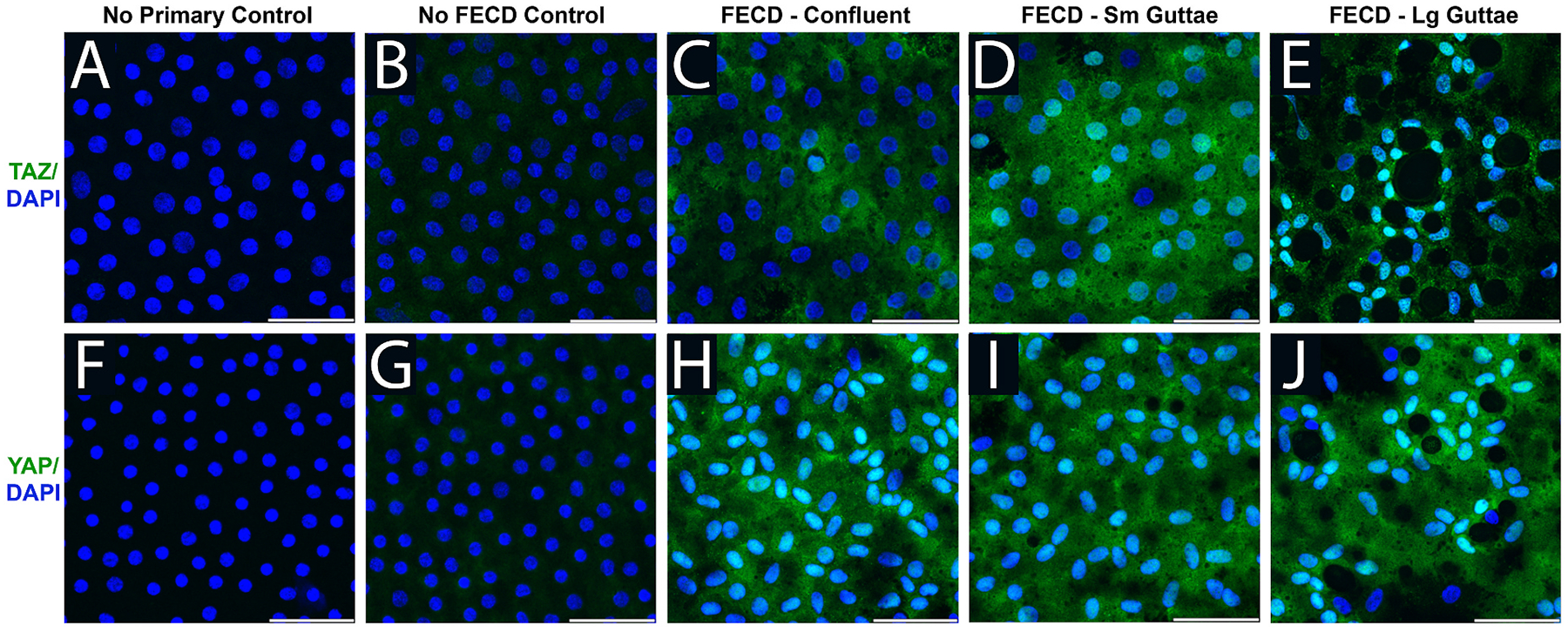
TAZ and YAP localize to the nucleus of corneal endothelial cells surrounding large guttae in FECD surgical explants compared to non-FECD healthy controls. Compared to healthy donor corneal endothelial tissues **(A, B, F, G)** isolated <18h after donor death and prior to storage in media, protein expression of both TAZ (green) and YAP (green) in age-matched FECD surgical samples (N = 7 and 3, respectively; **C-E, H-J**) is greater in all surgical tissues compared to controls. Expression of TAZ is greatest in the nucleus of corneal endothelial cells of FECD tissues in the central cornea adjacent to large guttae **(E)** compared to cells in the midperipheral cornea adjacent to smaller guttae **(D)**; expression of TAZ is greatest in the cytoplasm in the peripheral cornea where there are fewer guttae **(C)**. Similarly, expression of YAP is greatest in the nucleus of corneal endothelial cells in the central cornea adjacent to large guttae **(J)**, but in contrast, YAP nuclear expression is also increased in the midperiphery **(I)** and periphery **(H)** of FECD tissues. In aggregate, this pattern indicates translocation of TAZ and YAP from the cytosol to the nucleus (e.g., Hippo pathway is “off”) when corneal endothelial cells lose confluency or grow on top of or next to guttae, consistent with aberrant Hippo pathway mediated mechanotransduction in FECD progression. Images are representative samples. Nuclear counter stain = DAPI (blue). Scale bars are 50 μm. Figure and legend adapted from Leonard, B.C., Park, S., Kim, S., Young, L.J., Jalilian, I., Cosert, K., Zhang, X., Skeie, J.M., Shevalye, H., Echeverria, N., Rozo, V., Gong, X., Xing, C., Murphy, C.J., Greiner, M.A., Mootha, V.V., Raghunathan, V.K., Thomasy, S.M., 2023. Mice Deficient in TAZ (Wwtr1) Demonstrate Clinical Features of Late-Onset Fuchs’ Endothelial Corneal Dystrophy. Invest Ophthalmol Vis Sci 64, 22 with unpublished data.

The precise signaling events by which guttae induce alterations in corneal endothelial cell morphology and function remain understudied. Importantly, we recently investigated *WWTR1*/TAZ expression in ungenotyped *ex vivo* surgical explants from patients with FECD that underwent endothelial keratoplasty (Leonard et al., 2023). Western blotting quantitative analysis demonstrated a marked increase in total TAZ protein from FECD patients compared to age-matched donor tissue controls. Immunohistochemical staining showed an intense nuclear localization of TAZ in corneal endothelial cells, particularly in those located at the base of large guttae, indicating translocation of from the cytosol to the nucleus (e.g., Hippo pathway is “off”) when corneal endothelial cells lose confluency or are adjacent to guttae (Fig. 25A–E). We have demonstrated similar changes in YAP localization within corneal endothelial cells from similar surgical FECD explants (Fig. 25F–J). These findings strongly implicate aberrant Hippo pathway mediated mechanotransduction in FECD pathology.

### 5.5. Diabetes mellitus, an exemplar of secondary ECM pathology

Diabetes mellitus type II adversely impacts the health of the corneal endothelium as well as corneal transplant outcomes. The impact of this disease is expected to grow over the next several decades as the prevalence and incidence increase and the population ages. More than one-third of the donor cornea pool is already comprised of diabetic donor tissue (Liaboe et al., 2017), representing a disproportionately higher number compared to the ~11% of individuals living with diabetes. Diabetic donor tissue is adversely impacted by the disease process in a variety of ways including reduced ECD compared to age-matched controls (Liaboe et al., 2017), and animal models demonstrate similar findings (Meyer et al., 1988; Yee et al., 1985). Thus, donor corneal tissue can serve as a useful model of the impact of diabetes on the corneal endothelium. Specifically, it is associated with greater endothelial cell loss and higher rates of graft failure 3 years postoperatively after DSAEK (Lass et al., 2019). Prospective clinical trial data from the Diabetes Endothelial Keratoplasty Study (ClinicalTrials.gov identifier NCT05134480) will further clarify the role of diabetes on outcomes after endothelial keratoplasty. Additionally, the mitochondria of corneal endothelial cells from donors with advanced diabetic disease (insulin dependence with diabetic end-organ damage) demonstrate structural and functional impairment, thus reducing ATP production and pump function in the endothelium (Aldrich et al., 2017). Readers wishing to explore this topic further are directed to our extensive review of the impact of diabetes on the corneal endothelium (Goldstein et al., 2020). Specifically relevant to the relationship between corneal endothelial cells and their ECM, diabetic donor corneas prepared for use in DMEK, by peeling away the endothelium-DM tissue from the posterior stroma, have a significantly higher odds of tearing during preparation (odds ratio vs. age-matched non-diabetic controls, 9.2) (Greiner et al., 2014). Increased force is required to start and sustain DMEK peels, suggesting that increased adhesion between DM and posterior stroma contributes to graft preparation failure particularly in tissues from donors with advanced diabetes (Schwarz et al., 2016). In clinical practice, fragmentation of DM and endothelium also occurs during the resection of diseased tissue during endothelial keratoplasty in diabetic patients. Thus, diabetes mellitus can impact the functional health of the endothelium, particularly the ECM interface between DM and posterior stroma.

**Fig. 26.**
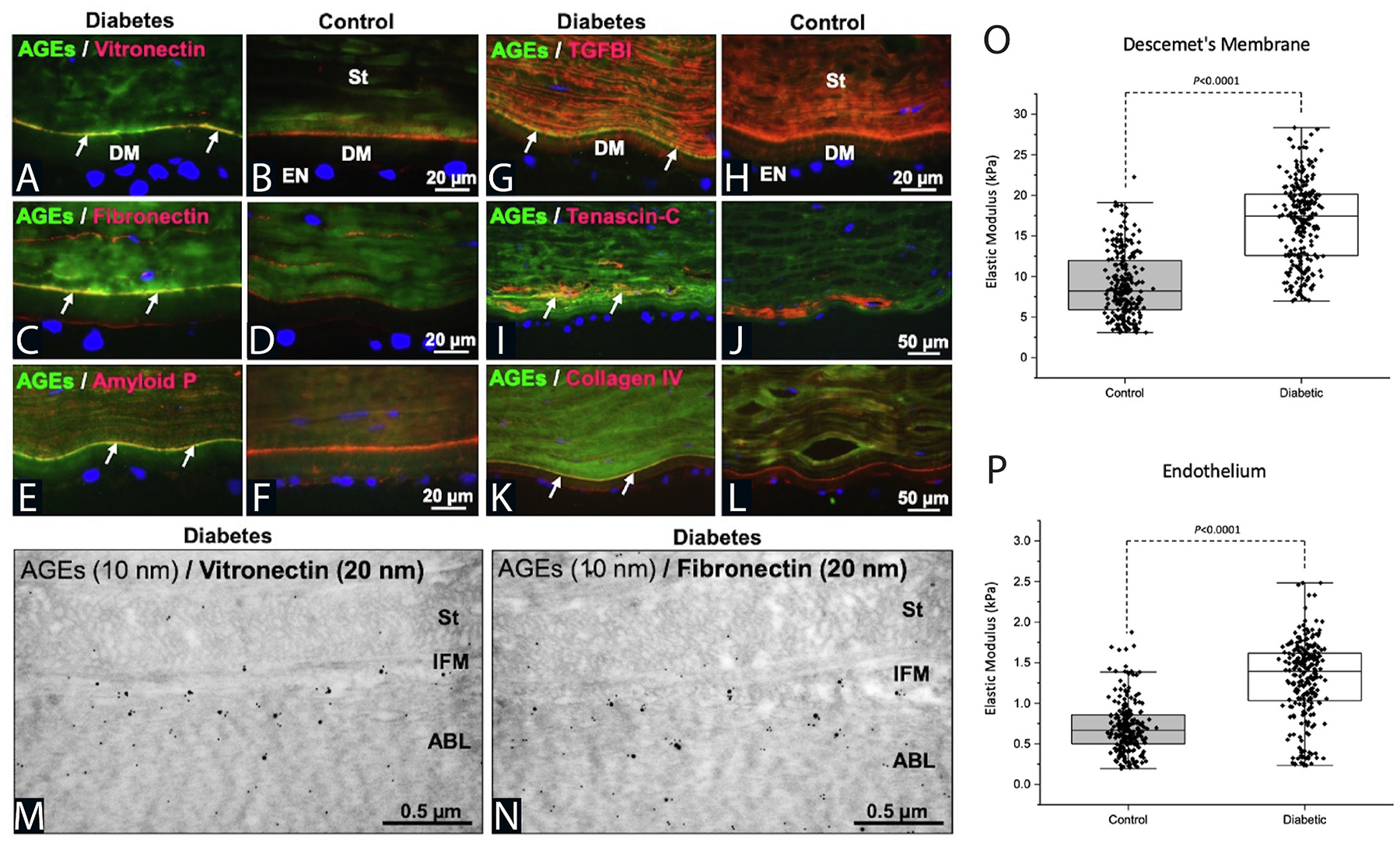
Extracellular matrix protein alterations are prevalent in diabetic donor corneas that mechanically alter DM and the corneal endothelium. Colocalization of adhesive glycoproteins with advanced glycation end products (AGEs) at the DM–stroma (St) interface in diabetic and normal control corneas. (A–L) Immunohistochemical double-labeling experiments showing differential staining patterns of the DM–St interface region (arrows) for AGEs and vitronectin (A, B), fibronectin (C, D), amyloid P (E, F), TGFBI (G, H), tenascin-C (**I**, J), and collagen IV (K, L). Nuclei are counterstained with DAPI (blue). EN, endothelium. (M, N) Immunogold double-labeling for AGEs (10-nm gold particles) and vitronectin (M) or fibronectin (N) (20-nm gold particles) in the IFM of diabetic corneas. ABL, anterior banded layer of the DM. These results indicate that AGE residues are localized specifically to the adhesive matrix proteins in the interfacial matrix of diabetic tissues. **(O, P)** The DM and endothelium were markedly stiffer in diabetic versus control corneas as determined by atomic force microscopy. The elastic modulus was measured in six diabetic and six age-matched control corneas and was significantly greater at 16.77 ± 5.01 and 8.95 ± 4.07 kPa for the DM and 1.29 ± 0.48 and 0.70 ± 0.31 kPa for the endothelium, respectively (P < 0.0001). Box plots depict the median (solid line) and 25th and 75th percentiles, and whiskers show the 10th and 90th percentiles. Black circles indicate individual measurements. Figure and legend adapted from Kingsbury, K.D., Skeie, J.M., Cosert, K., Schmidt, G.A., Aldrich, B. T., Sales, C.S., Weller, J., Kruse, F., Thomasy, S.M., Schlötzer-Schrehardt, U., Greiner, M.A., 2023. Type II Diabetes Mellitus Causes Extracellular Matrix Alterations in the Posterior Cornea That Increase Graft Thickness and Rigidity. Invest Ophthalmol Vis Sci 64, 26.

This interfacial matrix is a unique potential space that becomes manifest only with the application of surgical techniques and thus can yield important information about ECM disease processes. We recently investigated the impact of diabetes mellitus on the interfacial matrix cleavage plane given its high clinical relevance to DMEK graft preparation and resection of diseased tissue (Kingsbury et al., 2023). We demonstrated that diabetic hyperglycemia resulted in structural alterations to ECM proteins normally detectable in this region including vitronectin, fibronectin, TGFBI, collagen type IV, amyloid P and tenascin-C) through the formation of advanced glycation end products (AGEs) that result from nonenzymatic glycation and oxidation (Twarda-Clapa et al., 2022). Using transmission electron microscopy, DM was thicker in diabetic versus control corneas and increased with disease severity. Diabetic corneas had abnormal collagen type III inclusions in the posterior nonbanded layer adjacent to the endothelium with abnormal vacuolar inclusions that were frequently observed at the DM-stroma interface which increased its thickness. Increased staining intensity of adhesive glycoproteins were detected in advanced diabetic corneas compared to controls, indicating disease-related increased ECM protein expression. Importantly and only in diabetic corneas, increased AGE immunoreactivity was detected at the interfacial matrix with most adhesive glycoproteins co-localized with AGEs in this interfacial region. Furthermore, elastic modulus of DM and endothelium were significantly increased in diabetic corneas, consistent with the expected cross-linking effects that accompany AGE formation (Fig. 26). Lastly, using a controlled cell culture model of hyperglycemia, a dose-dependent relationship of ECM protein expression was noted along with AGE colocalization, supporting a direct relationship between diabetic hyperglycemia and ECM pathology in the posterior cornea. In aggregate, these data document marked changes in the corneal interfacial matrix in diabetic corneas with important biomechanical implications for both donor tissues and recipient patients.

### 5.6. Therapeutics that modulate endothelial-DM interactions

While ROCK inhibitors were originally developed to treat glaucoma by decreasing resistance to aqueous humor outflow (Karri and Chong, 2023), their impact on corneal endothelial health has been increasingly studied (Syed and Rapuano, 2021). The ROCK inhibitors form a complex with ROCK to block its ATP binding, and most act on ROCK1 and ROCK2 isoforms. In corneal endothelial cells, ROCK inhibitors can modulate cell adhesion, migration, proliferation, cell cycle progression, and viability (Bostan et al., 2016; Kinoshita et al., 2018; Koizumi et al., 2012; Okumura et al., 2012, 2013b, 2014b, 2016a, 2016b, 2016c). The three most widely studied ROCK inhibitors for use in corneal endothelial cells include Y-27632 (widely available as a research reagent but not clinically available), ripasudil (0.4%, Glanatec^®^, Kowa Pharmaceuticals), and netarsudil (0.02%, Rhopressa^®^, Aerie Pharmaceuticals) (Polopalli et al., 2023). The proliferative effects of these ROCK inhibitors lend the potential to treat diseases of the corneal endothelium, and clinical applications in the cornea are being actively investigated and will be discussed.

In a variety of corneal endothelial cell culture models, ROCK inhibition increases cell migration, proliferation, and adhesion while inhibiting apoptosis *in vitro* (Jung et al., 2021; Kim et al., 2022; Lee et al., 2016; Miron et al., 2023; Okumura et al., 2009; Peh et al., 2015; Toda et al., 2017). For example, Okumura and colleagues demonstrated Y27632 increased cell adhesion and proliferation and decreased apoptosis in cultured cynomolgus macaque endothelial cells (Okumura et al., 2009). ROCK inhibitors stimulate G1/S cell cycle progression by activating PI 3-kinase, upregulating cyclin D, and downregulating p27 thus initiating mitosis (Okumura et al., 2014b). Y27632 also mitigated TNFα stimulated monocyte chemoattractant protein-1 (MCP-1) and VEGF expression in porcine corneal endothelial cells suggesting that it also has an anti-inflammatory effect (Lee et al., 2016) These studies demonstrate a rapidly expanding literature on the effects of ROCK inhibition on corneal endothelial cell behavior and function *in vitro* but a knowledge gap remains regarding how they impact their ECM deposition. We recently described a model system using bovine corneal endothelial cells to produce cell-derived matrices (Jalilian et al., 2023) that could be utilized to study how ROCK inhibitors modulate cell-ECM interactions.

*In vivo* studies in animal models provide further evidence for the use of ROCK inhibitors as a therapy for corneal endothelial dysfunction. Y27632 and ripasudil accelerated corneal endothelial wound healing in healthy mice (Wang et al., 2022a), rabbits (Okumura et al., 2015, 2016b), dogs (Miyagi et al., 2019), and cynomolgus macaques (Okumura et al., 2013b), with injury induced using ultraviolet light, mechanical scraping, or transcorneal freezing. Furthermore, Y27632 also promoted adhesion of injected corneal endothelial cells in rabbits (Okumura et al., 2012), cats (Bostan et al., 2016), and NHPs (Okumura et al., 2012) with mechanical endothelial injury. Based on successes *in vitro* and *in vivo* using animal models, Okumura and colleagues utilized cryoinjury and topical Y27632 in 8 patients with corneal endothelial dysfunction and demonstrated variable efficacy (Okumura et al., 2013b). This group performed an additional pilot study with topical Y27632 only in 3 humans with traumatic endothelial injury and all improved (Okumura et al., 2015). These observations led the Kinoshita group to investigate whether corneal endothelial cells supplemented with Y-27632 and injected into patients could adhere to the posterior cornea and clear corneal edema (Kinoshita et al., 2018). Of 11 consecutive patients with bullous keratopathy and no detectable corneal endothelial cells treated with this therapy, all achieved the primary endpoint (>500 cells/mm^2^), 10 achieved a reduction in corneal thickness, and 9 improved best-corrected visual acuity by ≥ 2 lines at 24 weeks post injection. Of note, a silicone needle was used to remove abnormal ECM matrix and/or degenerated corneal endothelial cells on the patient’s DM in the central 8 mm of the cornea. The ability to achieve cell adhesion without complete resection of diseased DM using this approach (in contrast to conventional keratoplasty techniques such as DMEK) may be attributable to the impact of ROCK inhibition. However, additional investigations into optimal patient selection and surgical technique are required and we highlight that ROCK inhibitors are currently only approved for use in glaucoma.

Reports of spontaneous edema clearance after inadvertent resection of corneal endothelium during intraocular surgery or failed endothelial keratoplasty has led to the development of surgical techniques that resect diseased DM-endothelium while relying on endothelial migration and possibly proliferation to reform a monolayer (Van den Bogerd et al., 2018). In 2016, Borkar and coauthors reported successful Descemetorhexis without endothelial keratoplasty (DWEK), more recently termed Descemet’s stripping only (DSO), to treat early cases of FECD in which damage is limited to the central cornea (Borkar et al., 2016). In DSO, the central 4–5 mm of the endothelium-DM is removed, no donor tissue is transplanted, and healing occurs by migration and possibly proliferation of peripheral endothelial cells from the patient (He et al., 2012). This surgery addresses central guttae that are responsible for decreased vision in early FECD and facilitates migration of more normal peripheral endothelial cells over bare stroma than can produce a new DM with less pathology. During the healing phase, significant corneal edema appears overlying the area of bare stroma centrally with associated poor central vision with an average time to corneal clearance of 3 months with post-operative topical corticosteroids and 5% sodium chloride solution (Borkar et al., 2016; Davies et al., 2018). Failure rates of 9–40% are reported for DSO with patients requiring subsequent endothelial keratoplasty (Din et al., 2022; Iovieno et al., 2022; Vieira et al., 2023). Furthermore, return of guttae with reduced ECD and endothelial dysfunction can occur post-operatively (Kaufman et al., 2023) suggesting that treatments that can promote a healthier DM are warranted.

**Fig. 27.**
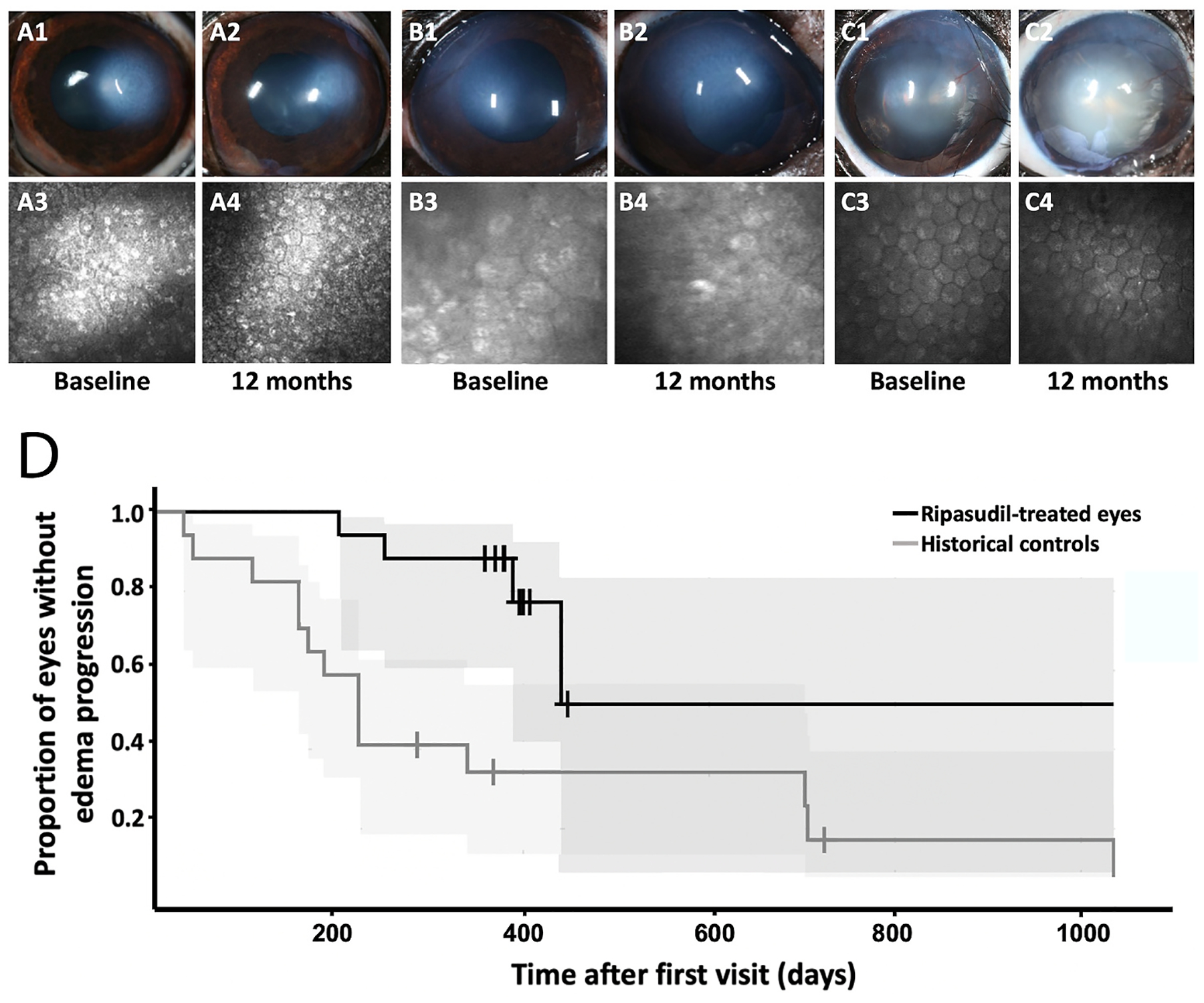
Response to topical ripasudil four times daily was variable in CED-affected dogs with 62% demonstrating stable disease or improvement while 38% had disease progression. Representative digital photographs and IVCM images of CED-affected eyes at baseline and 12-months post treatment **(A)** An 8-year-old male castrated Boston terrier demonstrated improved corneal edema **(A1 to A2)** and improved endothelial cell density (ECD, **A3 to A4**), increasing from 1565 ± 150 cells/mm^2^ at baseline to 1733 ± 224 cells/mm^2^ at 12 months. **(B)** A 5-year-old male castrated chihuahua mix demonstrated progressive corneal edema **(B1 to B2)** and decreased ECD **(B3 to B4)** from 1324 ± 138 cells/mm^2^ at baseline to 881 ± 17 cells/mm^2^ at 12 months. **(C)** A 12-year-old male castrated shih tzu demonstrated stable corneal edema **(C1 to C2)** and stable ECD **(C3 to C4)** from 1674 ± 199 cells/mm^2^ at baseline to 1588 ± 30 cells/mm^2^ at 12 months. The ECD is reported as mean ± SD. **Kaplan-Meier curves demonstrated that CED-affected corneas treated with topical ripasudil progressed slower than historical controls (D)**. Tick marks indicate censored subjects, and the shaded area indicates 95% confidence intervals. Median time from the initial visit to meeting any of the progression criteria in historical controls was 223 days. Median time to progression was not reached in ripasudil-treated eyes. Log-rank test identified a significant difference in distribution of time to progression between eyes receiving topical ripasudil and untreated historical controls (*P* = 0.023). Seventeen ripasudil-treated eyes and 17 untreated, age- and breed/size-matched historical controls were included in the analysis. Adapted from Michalak, S.R., Kim, S., Park, S., Casanova, M.I., Bowman, M.A.W., Ferneding, M., Leonard, B.C., Good, K.L., Li, J.Y., Thomasy, S.M., 2022. Topical Ripasudil for the Treatment of Primary Corneal Endothelial Degeneration in Dogs. Transl Vis Sci Technol 11, 2.

To improve corneal clearance and vision recovery after DSO, the adjunctive use of topical ROCK inhibitors has been investigated with initial success reported by Moloney using ripasudil (Moloney et al., 2017). Since then, other groups have reported on ripasudil and netarsudil as potential adjuvants or rescue treatments in DSO due to their effects on cell cycle activation, cell adhesion, and migration in the corneal endothelium (Davies, 2021; Davies et al., 2021; Goldstein et al., 2018; Moloney et al., 2021; Ploysangam and Patel, 2019; Schlötzer-Schrehardt et al., 2021). In a recent meta-analysis by Din and colleagues reporting on 68 eyes from 11 studies (mean follow-up, 12.4 ± 11 months), treatment with ripasudil after DSO did not improve surgical success but did shorten the time to corneal clearance (4.9 ± 1.8 vs. 10.1 ± 5.9 weeks) (Din et al., 2022). Additionally, this analysis noted both increased central and decreased peripheral ECD, consistent with wound closure achieved by peripheral corneal endothelial cell migration rather than proliferation. Early clinical reports also suggest that netarsudil may be effective in achieving clearance of corneal edema after DSO (Davies et al., 2021). However, clinical trials comparing their efficacy have not been conducted and it is unknown whether both can be used interchangeably. The optimal dosing frequency and duration for ROCK inhibitors after DSO in FECD patients also have not been determined (reported in a meta-analysis by Francheschino and colleagues to be 4–6 times a day for 2–8 weeks for primary treatments, and 2 times a day in the event of relapse edema) (Franceschino et al., 2022). Given the impact of ROCK inhibitors on ECM deposition, remodeling and tension in corneal epithelial and stromal cells (Kim et al., 2006; Kim and Matthew Petroll, 2007; Petroll et al., 2004, 2008a, 2008b; Svoboda et al., 2004), it is interesting to speculate about how their use modulates DM reformation following DSO. Indeed, Y27632 treatment of corneal stromal cells *in vitro* markedly altered collagen matrix remodeling with decreased fibril density and alignment (Kim et al., 2006). In a rabbit superficial keratectomy model, bundles of aligned and uniformly spaced collagen fibrils consistent with embryonic connective tissue were more prevalent in keratocytes in Y27632-treated corneas (Yamamoto et al., 2012). These data suggest that there may be a role for long-term treatment with ROCK inhibitors in promoting a healthier endothelium and DM in FECD patients with and without DSO. This premise is further supported by our recent study demonstrated that ripasudil administration for 1 year in dogs with primary corneal endothelial degeneration improved or stabilized disease in 62% of patients (Fig. 27) (Michalak et al., 2022). In aggregate, there is a pressing need to study how ROCK inhibitors modulate ECM deposition and remodeling in corneal endothelial cells from FECD patients *in vitro* as well as in relevant animal models and clinical trials.

## 6. Conclusions and future studies

As is evident, we and others have made substantial progress in understanding the mechanobiological regulation of the ECM contributions to corneal disorders from epithelium to endothelium. Nevertheless, as this comprehensive review demonstrates much work remains in order to translate these findings into a clear clinical strategy to restore vision and optimize visual performance in patients with corneal disease. As discussed in detail for each cell type, the forces encountered by the relevant cell type differ, as does the biochemical composition of the microenvironment, and consequently the signaling events that govern cell fate decisions in response to homeostasis and pathologic stimuli. The application of these concepts in a cell- and disease-context is of particular interest as is the use of preclinical models that better recapitulate the disorder of interest. While this complexity highlights the challenges, rapid advances in technology, conceptual innovation, molecular tools, development of complex cell culture models, and use of relevant animal models converge to highlight the critical need for an interdisciplinary approach to identifying and testing new therapies for corneal disorders. Of particular importance, it is imperative for the vision science community to take note that individual signaling pathways do not act in isolation and multiple pathways crosstalk at various levels to compensate or adversely affect cell fate decisions. Further, the ability of cells to sense and respond to the various stimuli is highly dependent on the bidirectional dynamic alterations and interactions between cells and their microenvironment. This is clearly manifest from the challenges we’ve observed in translating *in vitro* findings to preclinical *in vivo* models. As such, the continued utility of singular cell types in a non-physiological cell culture platform needs critical introspection and reevaluation. The body of evidence presented here demonstrates that biomechanical and biochemical alterations within the microenvironment tightly regulate corneal epithelial barrier integrity, stromal organization, and endothelial pump function in homeostasis. Thus, perturbances in this well-orchestrated homeostasis in any of these corneal cell types potentially can have permeating and devastating consequences on the others to further compromise corneal function. Thus, a one-size-fits-all approach to understanding the molecular underpinnings will need to be replaced with better-engineered complex models and biologically relevant animal models which integrate mechanical and chemical cues in a cell type dependent manner to further our quest in identifying a cure for corneal diseases.

## Data Availability

Data will be made available on request.
